# Global well-posedness of the 2D nonlinear Schrödinger equation with multiplicative spatial white noise on the full space

**DOI:** 10.1007/s00440-024-01288-y

**Published:** 2024-06-22

**Authors:** Arnaud Debussche, Ruoyuan Liu, Nikolay Tzvetkov, Nicola Visciglia

**Affiliations:** 1 CNRS, IRMAR - UMR 6625, Univ Rennes, 35000 Rennes, France; 2 School of Mathematics, The University of Edinburgh and The Maxwell Institute for the Mathematical Sciences, James Clerk Maxwell Building, The King’s Buildings, Peter Guthrie Tait Road, Edinburgh, EH9 3FD UK; 3 UMPA, UMR CNRS-ENSL 5669, Ecole Normale Supérieure de Lyon, 46, allée d’Italie, 69364 Lyon Cedex 07, France; 4 Dipartimento di Matematica, Università di Pisa, Largo Bruno Pontecorvo, 5, 56100 Pisa, Italy

**Keywords:** Nonlinear Schrödinger equation, Multiplicative white noise, Global well-posedness, 35Q55, 60H15

## Abstract

We consider the nonlinear Schrödinger equation with multiplicative spatial white noise and an arbitrary polynomial nonlinearity on the two-dimensional full space domain. We prove global well-posedness by using a gauge-transform introduced by Hairer and Labbé (Electron Commun Probab 20(43):11, 2015) and constructing the solution as a limit of solutions to a family of approximating equations. This paper extends a previous result by Debussche and Martin (Nonlinearity 32(4):1147–1174, 2019) with a sub-quadratic nonlinearity.

## Introduction

### NLS with multiplicative space white noise

We study the following Cauchy problem on $\mathbb R^2$ for the stochastic defocusing nonlinear Schrödinger equation (NLS)

1.1 $$\begin{aligned} \imath \partial _t u =\Delta u+ u\xi -\lambda u|u|^{p}, \quad u(0)=u_0, \end{aligned}$$

where $p>0,\,\lambda >0$ are parameters and $\xi \in \mathcal {S}'(\mathbb R^2)$ stands for white noise in space. The unknown *u* is a complex valued process and $u_0$ is a randomized initial datum (more precise assumptions are given below).

One can view the NLS (1.1) as a stochastic version of the deterministic nonlinear Schrödinger equation with a power type nonlinearity, which has been studied extensively in recent decades. See [6, 8, 9, 25] and the references therein. On the other hand, if we ignore the nonlinearity, (1.1) can be viewed as the dispersive Anderson model, which is the dispersive counterpart of the well-studied parabolic Anderson model (see, for example, [20, 21]), i.e. with $\imath \partial _t u$ replaced by $\partial _t u$.

The NLS (1.1) was first considered by the first author and Weber in [13] on a periodic domain $\mathbb T^2 = (\mathbb R/ \mathbb Z)^2$. To deal with the ill-defined nature of the term $u \xi$, they used the gauge transform $v = e^{Y} u$ where *Y* solves $\Delta Y = \xi$. This gauge transform, which resembles the so-called Doss-Sussmann transformation in [14, 38], was first introduced by Hairer and Labbé in the context of the parabolic Anderson model on $\mathbb R^2$ in [20] (the definition for *Y* is slightly different on $\mathbb R^2$). The equation for *v* now formally reads as

$$\begin{aligned} \imath \partial _t v = \Delta v - 2 \nabla v \cdot \nabla Y + v \nabla Y^2 - \lambda e^{-p Y} v |v|^p, \quad v(0) = e^Y u_0 , \end{aligned}$$

which is easier to solve since the most singular term is canceled. Still, the term $\nabla Y$ is merely a distribution, so that $\nabla Y^2$ is replaced by a Wick product $\mathbf {:}\nabla Y^2\mathbf {:}$ in [13, 20]. See the next subsection for more detailed explanations.

In [13], the first author and Weber showed global well-posedness of the following gauge-transformed NLS on $\mathbb T^2$ with a (sub-)cubic nonlinearity (i.e. with $p \le 2$):

1.2 $$\begin{aligned} \imath \partial _t v = \Delta v - 2 \nabla v \cdot \nabla Y + v \, \mathbf {:}\nabla Y^2\mathbf {:}- \lambda e^{-p Y} v |v|^p, \quad v(0) = e^Y u_0. \end{aligned}$$

The main strategy is to consider a mollified noise $\xi _\varepsilon$ and a smoothed process $Y_\varepsilon = \Delta ^{-1} \xi _\varepsilon$, and then construct the solution *v* as a limit of $v_\varepsilon$ in probability, which is the solution of the following smoothed equation:

$$\begin{aligned} \imath \partial _t v_\varepsilon = \Delta v_\varepsilon - 2 \nabla v_\varepsilon \cdot \nabla Y_\varepsilon + v_\varepsilon \, \mathbf {:}\nabla Y^2_\varepsilon \mathbf {:}- \lambda e^{-p Y_\varepsilon } v_\varepsilon |v_\varepsilon |^p, \quad v_\varepsilon (0) = e^Y u_0. \end{aligned}$$

The key ingredient for the convergence of $v_\varepsilon$ is a suitable $H^2$ a-priori bound for $v_\varepsilon$ with a logarithmic loss in $\varepsilon$.

Later on, the third author and the fourth author [41] improved the result in [13] by extending global well-posedness of the gauge-transformed NLS (1.2) on $\mathbb T^2$ to the larger range $p \le 3$. Specifically, they introduced modified energies that allow them to control the $H^2$ a-priori bound of $v_\varepsilon$ for a larger range of *p*. In a subsequent paper [42], the third author and the fourth author improved their global well-posedness result by covering all $p > 0$. In particular, they exploited the time averaging effect for dispersive equations and established Strichartz estimates to obtain the $H^2$ a-priori bound of $v_\varepsilon$ for the whole range $p > 0$. Moreover, in [41, 42], the authors improved [13] by proving almost sure convergence of $v_\varepsilon$ to *v* instead of convergence in probability.

We now turn our attention to the NLS (1.1) on the $\mathbb R^2$ setting, which is the main concern in this paper. In this situation, the additional difficulty comes from the logarithmic growth of the white noise. In [12], the first author and Martin showed global well-posedness of a gauge-transformed NLS similar to (1.2) (see (1.7) below) for $0< p < 1$. To conquer the issue of the unboundedness of the noise, they used weighted Sobolev and Besov spaces and obtained a weighted $H^2$ a-priori bound for $v_\varepsilon$ with a logarithmic loss in $\varepsilon$. This approach of using the weighted Besov spaces in the study of stochastic PDEs was also used in [20, 21, 30, 34]. In the case of the NLS (1.1), such an approach requires more assumptions on the regularity of the initial datum than those on the $\mathbb T^2$ setting.

In this paper, we extend the result in [12] by including all $p > 0$ cases using an intricate combination of the methods mentioned above: the weighted Sobolev and Besov spaces estimates, the modified energies as in [41], and the dispersive effect as in [42]. In addition, we are able to prove a stronger convergence result of $v_\varepsilon$ to *v* (almost sure convergence) than that in [12] (convergence in probability). This is obtained thanks to the estimates on the white noise on $\mathbb R^2$ and Wick products which are of independent interest. See the next subsection for a more detailed set-up and the main result of this paper.

### Set-up and the main result

Let us recall that, given a probability space $(\Omega , \mathcal F,\mathbb P)$, a real valued white noise on $\mathbb R^2$ is a random variable $\xi :\; \Omega \rightarrow \mathcal D'(\mathbb R^2)$ such that for each $f\in \mathcal D(\mathbb R^2)$, $(\xi , f)$ is a real valued centered Gaussian random variable such that $\mathbb E((\xi ,f)^2)=\Vert f\Vert _{L^2}^2$. Along the rest of the paper, $(\cdot ,\cdot )$ will denote the duality $\mathcal D(\mathbb R^2)$, $\mathcal D'(\mathbb R^2)$ and hence in the special case of classical functions, this is the usual $L^2$ scalar product. Classically, $f\rightarrow (\xi ,f)$ can be extended uniquely from $\mathcal D(\mathbb R^2)$ to $L^2(\mathbb R^2)$, and $(\xi ,f)$ is a real valued centered Gaussian random variable such that $\mathbb E((\xi ,f)^2)=\Vert f\Vert _{L^2}^2$ for all $f\in L^2(\mathbb R^2)$.

We proceed as in [20] and use a truncated Green’s function $G\in C^\infty (\mathbb R^2\backslash \{0\})$ that satisfies $\textrm{supp}\,G \subseteq B(0,1)$ (the unit ball around 0) and $G(x)=-\frac{1}{2\pi } \log |x|$ for |*x*| small enough, so that $Y:=G*\xi$ solves

1.3 $$\begin{aligned} \Delta Y=\xi +\varphi *\xi \end{aligned}$$

for some $\varphi \in C^\infty _c(\mathbb R^2)$. Hence, we introduce the new variable

1.4 $$\begin{aligned} v=e^{Y} u \end{aligned}$$

which converts (1.1) into the following gauge-transformed NLS for *v*:

$$\begin{aligned} \imath \partial _t v=\Delta v+v(\,\nabla Y^2-\varphi *\xi )-2\nabla v \cdot \nabla Y-\lambda v|v|^{p} e^{-p Y} ,\quad v(0)=v_0:=e^{Y} u_0\,. \end{aligned}$$

The term $\nabla Y^2$ is ill-defined as a square of a distribution, but can be replaced by a meaningful object $\mathbf {:}\nabla Y^2\mathbf {:}$, which is essentially the Wick product of $\nabla Y$ with itself. As in [20], we introduce the random variable

1.5 $$\begin{aligned} \mathbf {:}\nabla Y^2\mathbf {:}= \int _{\mathbb R^2} \int _{\mathbb R^2} \nabla G(\cdot -z_1) \nabla G(\cdot -z_2) \varvec{\xi }(\textrm{d}z_1)\varvec{\xi }(\textrm{d}z_2) \end{aligned}$$

with $\varvec{\xi }$ denoting the Gaussian stochastic measure on $\mathbb R^2$ induced by the white noise $\xi$ (see [24, page 95-99]). The relation (1.5) should be read in the *distributional* sense, i.e. for $\phi \in \mathcal {S}(\mathbb R^2)$ we have

$$\begin{aligned} \mathbf {:}\nabla Y^2\mathbf {:}(\phi ) = \int _{\mathbb R^2} \int _{\mathbb R^2} \Big (\int _{\mathbb R^2} \, \phi (x)\nabla G(x -z_1) \nabla G(x -z_2) \textrm{d}x\Big ) \varvec{\xi }(\textrm{d}z_1)\varvec{\xi }(\textrm{d}z_2), \end{aligned}$$

so that $\mathbf {:}\nabla Y^2\mathbf {:}$ is only defined (almost surely) as a distribution. Recall that for $f_1,\,f_2\in L^2(\mathbb R^2)$ we have the following identity for $X_1:=\int _{\mathbb R^2} f_1(z_1) \varvec{\xi }(\textrm{d}z_1),\,X_2:=\int _{\mathbb R^2} f_2(z_2) \varvec{\xi }(\textrm{d}z_2)$ (see [24, Theorem 7.26])

1.6 $$\begin{aligned} \mathbf {:}X_1\cdot X_2\mathbf {:}=\int _{\mathbb R^2} \int _{\mathbb R^2} f_1(z_1) f_2(z_2)\varvec{\xi }(\textrm{d}z_1) \varvec{\xi }(\textrm{d}z_2), \end{aligned}$$

where $\mathbf {:}X_1\cdot X_2\mathbf {:}$ denotes the Wick product between the Gaussian random variables $X_1,\,X_2$. Note that the above integral is a multiple Wiener-Ito integral (see [33]). From this perspective, the definition $\mathbf {:}\nabla Y^2\mathbf {:}$ can be read as a Wick product of the distribution $\nabla Y$ with itself. For an introduction to Wick calculus let us refer to [22, 24, 33]. Thus, we shall focus on the following equation

1.7 $$\begin{aligned} \imath \partial _t v=\Delta v+v\,\widetilde{\mathbf {:}\nabla Y^2\mathbf {:}}-2\nabla v \cdot \nabla Y-\lambda v|v|^{p} e^{-p Y},\quad v(0)=v_0, \end{aligned}$$

where

1.8 $$\begin{aligned} \widetilde{\mathbf {:}\nabla Y^2\mathbf {:}}= \mathbf {:}\nabla Y^2\mathbf {:}- \varphi *\xi . \end{aligned}$$

In order to construct a solution to (1.7) we consider an approximation $v_\varepsilon$ which solves

1.9 $$\begin{aligned} \imath \partial _t v_\varepsilon =\Delta v_\varepsilon +v_\varepsilon \widetilde{\mathbf {:}\nabla Y^2_\varepsilon \mathbf {:}}-2\nabla v_\varepsilon \cdot \nabla Y_\varepsilon -\lambda v_\varepsilon |v_\varepsilon |^p e^{-pY_\varepsilon }, \quad v_\varepsilon (0)=v_0. \end{aligned}$$

Here, $Y_\varepsilon$ is defined as

$$Y_\varepsilon = \rho _\varepsilon *Y = \rho _\varepsilon *G *\xi = G *\xi _\varepsilon ,$$

where $\rho _\varepsilon (x) = \varepsilon ^{-2} \rho (\varepsilon ^{-1} x)$, $\rho \in C_c^{\infty } (B(0,1))$, $\rho \ge 0$, $\int _{\mathbb R^2} \rho = 1$, and $\xi _\varepsilon =\rho _\varepsilon *\xi$ is a mollification of the considered noise. Also,

1.10 $$\begin{aligned} \widetilde{\mathbf {:}\nabla Y^2_\varepsilon \mathbf {:}}=\mathbf {:}\nabla Y^2_\varepsilon \mathbf {:}-\varphi *\xi _\varepsilon , \end{aligned}$$

where the Wick product $\mathbf {:}\nabla Y^2_\varepsilon \mathbf {:}$ is defined as follows:

1.11 $$\begin{aligned} \mathbf {:}\nabla Y^2_\varepsilon \mathbf {:}(x) = \nabla Y_\varepsilon ^2(x)-c_\varepsilon , \quad c_\varepsilon = \mathbb E\left( |\nabla Y_\varepsilon |^2\right) = \Vert \rho _\varepsilon *\nabla G\Vert _{L^2}^2. \end{aligned}$$

We show in this paper that $Y_\varepsilon$ converges to *Y*, $\nabla Y_\varepsilon$ converges to $\nabla Y$, and $\mathbf {:}\nabla Y^2_\varepsilon \mathbf {:}$ converges to $\mathbf {:}\nabla Y^2\mathbf {:}$ almost surely in certain function spaces (see Proposition 3.8).

In the sequel we shall use weighted Sobolev spaces, where the details are in Sect. 2. For the moment, we can consider the following equivalent norm on the space $H^s_{\delta }(\mathbb R^2)$:

$$\begin{aligned} \Vert \langle x\rangle ^\delta \varphi \Vert _{H^s(\mathbb R^2)}, \quad s\ge 0, \quad \delta \in \mathbb R, \end{aligned}$$

which reduces the weighted Sobolev spaces to the usual unweighted Sobolev spaces once the weight is pulled inside.

We now state the following result regarding global well-posedness of the equation (1.9) for $v_\varepsilon$.

#### Theorem 1.1

There exists a full measure event $\Sigma \subset \Omega$ such that for every $\omega \in \Sigma$ and every $\varepsilon \in (0, \frac{1}{2})$, the following property holds. For any $p \ge 1$, $\delta _0>0$, $s\in (1,2)$, and $\delta > 0$, there exists $\delta _1 > 0$ such that the Cauchy problem (1.9) with $v_0 \in H^2_{\delta _0}(\mathbb R^2)$ admits one unique global solution

$$v_\varepsilon (t,x)\in L_{loc }^\infty ((0, \infty ); H^2_{-\delta }(\mathbb R^2))\cap \mathcal {C}([0, \infty ); H^s_{\delta _1}(\mathbb R^2)).$$

Moreover, for every $T>0$ and $\delta >0$ there exists constants $C, C(\omega ) > 0$ independent of $\varepsilon$ such that the following bound holds:

$$\begin{aligned} \Vert v_{\varepsilon }(t,x)\Vert _{L^\infty ((0,T);H^2_{-\delta }(\mathbb R^2))}\le C(\omega ) |\ln \varepsilon |^{C}. \end{aligned}$$

The existence and uniqueness of solution to (1.9) is not obvious since the coefficients involved in the linear propagator associated with (1.9) are smooth but unbounded. At the best of our knowledge, because of the lack of decay at the spatial infinity of the derivatives of $\xi _\varepsilon$ the classical Strichartz estimates are not available in this framework, and so we cannot apply a classical contraction argument. In fact, along the paper we shall establish some weighted Strichartz estimates that will allow us to perform a compactness argument as in [12] and to deduce the existence of solutions to (1.9) as the limit of solutions $v_{\varepsilon , n}$ to the following further regularized equation at $\varepsilon >0$ fixed:

1.12 $$\begin{aligned}{} & {} \begin{aligned} \imath \partial _t v_{\varepsilon , n}&=\Delta v_{\varepsilon , n}+v_{\varepsilon , n} \theta _n\widetilde{\mathbf {:}\nabla Y^2_\varepsilon \mathbf {:}}-2\nabla v_{\varepsilon , n} \cdot \nabla (\theta _n Y_\varepsilon ) -\lambda v_{\varepsilon , n} |v_{\varepsilon , n}|^p e^{-p\theta _n Y_\varepsilon }, \\ \quad v_{\varepsilon , n}(0)&=v_0, \end{aligned} \end{aligned}$$

where $\theta _n(x)=\theta (\frac{x}{n} )$ and $\theta \in C^\infty _0(\mathbb R^2)$, $\theta \ge 0$, $\theta (x)=1$ when $|x| \le 1$. To see that (1.12) is globally well-posed, we let $u_{\varepsilon , n} = e^{- \theta _n Y_\varepsilon } v_{\varepsilon , n}$ and consider the following equation for $u_{\varepsilon , n}$:

1.13 $$\begin{aligned} \begin{aligned} \imath \partial _t u_{\varepsilon , n}&=\Delta u_{\varepsilon , n}+u_{\varepsilon , n} ( \theta _n\widetilde{\mathbf {:}\nabla Y^2_\varepsilon \mathbf {:}}- \nabla (\theta _n Y_\varepsilon )^2 + \Delta (\theta _n Y_\varepsilon ) ) -\lambda u_{\varepsilon , n} |u_{\varepsilon , n}|^p, \\ u_{\varepsilon , n}(0)&= e^{-\theta _n Y_\varepsilon } v_0. \end{aligned} \end{aligned}$$

Note that $\theta _n Y_\varepsilon$ is a Schwartz function, so that $e^{-\theta _n Y_\varepsilon } v_0 \in H^2 (\mathbb R^2)$ given $v_0 \in H^2 (\mathbb R^2) \subset H^2_{\delta _0} (\mathbb R^2)$. Since the equation (1.13) contains only bounded and smooth terms, by classical results as in [9, 16, 25], there exists a unique solution $u_{\varepsilon , n}$ to (1.13) in $\mathcal {C} ([0, \infty ); H^2 (\mathbb R^2))$. This shows that there exists a unique solution $v_{\varepsilon , n}$ to (1.12) in $\mathcal {C} ([0, \infty ); H^2 (\mathbb R^2))$.

Once we establish a weighted $H^2$ a-priori bound (independent of *n*) for $v_{\varepsilon , n}$, we can use a similar argument in [12] to prove Theorem 1.1. See Sect. 8 for a more detailed explanation.

Next, we describe the behavior of the solutions $v_\varepsilon (t,x)$ in the limit $\varepsilon \rightarrow 0$. It is remarkable that this result can be also interpreted in terms of the convergence, up to a phase shift, of $u_\varepsilon (t,x)$ solutions to the smoothed version of (1.1)

1.14 $$\begin{aligned} \imath \partial _t u_\varepsilon =\Delta u_\varepsilon + u_\varepsilon \xi _\varepsilon -\lambda u_\varepsilon |u_\varepsilon |^{p}, \quad u(0)=u_0. \end{aligned}$$

It is worth mentioning that it is not obvious to prove directly the existence and uniqueness of solutions to the smoothed equation above. Nevertheless, by direct computation, one can show that

1.15 $$\begin{aligned} u_\varepsilon =e^{- \imath c_\varepsilon t} e^{-Y_\varepsilon } v_\varepsilon \end{aligned}$$

solves (1.14) provided that $v_\varepsilon$ is given by Theorem 1.1.

We are now ready to state the main result of this paper.

#### Theorem 1.2

There exists a full measure event $\Sigma \subset \Omega$ such that for every $\omega \in \Sigma$, the following property holds. Let $\delta _0 > 0$ and $v_0 \in H^2_{\delta _0}$. For any $p \ge 1$, $s \in (1, 2)$, and $\delta > 0$, there exists $v \in \mathcal {C} ([0, \infty ); H^s_{\delta _1} (\mathbb R^2))$ for some $\delta _1 > 0,$ such that the following convergence holds:

1.16 $$\begin{aligned} \Vert v_\varepsilon (t,x)-v(t,x)\Vert _{\mathcal {C} ([0,T); H^s_{\delta _1}(\mathbb R^2))}\overset{\varepsilon \rightarrow 0}{\longrightarrow }0, \end{aligned}$$

where $v_\varepsilon$ is given by Theorem 1.1 with $v_\varepsilon (0) = v_0$. In particular, $u_\varepsilon = e^{-\imath c_\varepsilon t} e^{-Y_\varepsilon } v_\varepsilon$ solves (1.14) and

$$\begin{aligned} \Vert e^{\imath c_\varepsilon t} e^{Y_\varepsilon } u_\varepsilon (t,x)-v(t,x)\Vert _{\mathcal {C} ([0,T); H^s_{\delta _1}(\mathbb R^2))}\overset{\varepsilon \rightarrow 0}{\longrightarrow }0, \end{aligned}$$

where $c_\varepsilon \sim |\ln \varepsilon |$ is the constant in (1.11). Moreover, *v* is the unique global solution to (1.7) in $\mathcal {C}([0, \infty ); H^s_{\delta _1}(\mathbb R^2))$.

Theorem 1.2 is an extension of previous result proved in [12] in the case $0< p < 1$. In particular, we cover the relevant case of cubic nonlinearity. Notice also that our convergence occurs almost surely, which is stronger than the result in [12] where the convergence is in probability.

We conclude this subsection by stating several remarks.

#### Remark 1.3

Throughout the whole paper, we restrict our attention to the case $\lambda > 0$ of the NLS (1.1), which refers to the defocusing case. For the focusing case (i.e. $\lambda < 0$) of (1.1), Theorem 1.2 also holds for $0< p < 2$. For $\lambda < 0$ and $p \ge 2$, we need to impose a smallness assumption on the initial data $\Vert v_0 \Vert _{H^1_{\delta _0}}$ in order to obtain Theorem 1.2. Indeed, the only place that requires a different proof for this case is Proposition 5.1. See Remark 5.2 for details.

#### Remark 1.4

For the NLS (1.1) in higher dimensions, it is not clear whether the approach in this paper based on a gauge-transform works in showing global well-posedness. The main challenge lies in the fact that the spatial white noise is too rough for our approach when $d \ge 3$. Another challenge is the weaker smoothing properties of the Schrödinger equation in higher dimensions. One can compare the situation with the parabolic setting in [21], where the authors used the theory of regularity structures due to Hairer [19].

#### Remark 1.5

The authors in [18, 31, 43] introduced another approach to the study of the NLS (1.1) with $p \le 2$. Their method is based on the realization of the (formal) Anderson Hamiltonian $H = \Delta + \xi$ as a self-adjoint operator on the $L^2$ space. Specifically, [18] considered the equation on the torus, [31] considered a compact manifold, and [43] considered the full space. In their settings, the initial data $u_0$ needs to belong to the domain of *H*. One can compare the initial condition in [18, 43] and that in this paper and in [12, 13, 41, 42], where the initial data is chosen to have a specific structure $e^{-Y} v_0$ with $v_0$ belonging to a weighted $H^2$ space. For more discussions on the Anderson Hamiltonian, see [1, 3, 10, 29, 32].

### Notations

We denote by $\mathcal {P}(a,b,\dots )$ a polynomial function depending on the $a,b, \dots$. The polynomial can change from line to line along the estimates. For any positive number $a > 0$, we use $a^+$ to denote $a + \eta$ for $\eta > 0$ arbitrarily small. We denote by *N* dyadic numbers larger than or equal to $\frac{1}{2}$ and by $\Delta _N$ the corresponding Littelwood-Paley partition. All the functional spaces that we shall use are based on $\mathbb R^2$. We denote by $C>0$ a deterministic constant that can change from line to line and $C(\omega )>0$ a stochastic constant which is finite almost surely. We denote by $(\cdot , \cdot )$ the $L^2$ scalar product as well as the duality in $\mathcal {D(\mathbb R^2)}, \mathcal {D}'(\mathbb R^2)$.

### Organization of the paper

This paper is organized as follows. In Sect. 2, we recall the definitions of some useful functional spaces and their properties. In Sect. 3, we discuss properties of the process *Y* and its related stochastic objects. In Sect. 4 and Sect. 5, we establish some useful linear and nonlinear estimates for a generalized equation. In Sect. 6, we recall definitions of modified energies and provide estimates for them. In Sect. 7, we prove a key $H^2$ a-priori bound. Lastly, in Sect. 8, we prove the main result of this paper: Theorem 1.2.

## Functional spaces and preliminary facts

### Functional spaces

Given $p\in [1,\infty ],\,\mu \in \mathbb R$ we introduce respectively the weighted Lebesgue and Sobolev spaces as follows:

$$\begin{aligned} \Vert f\Vert _{L^p_\mu }=\Big (\int _{\mathbb R^2} \langle x\rangle ^{\mu p}|f|^p dx\Big )^\frac{1}{p} , \quad \langle x\rangle =\sqrt{1+|x|^2} \end{aligned}$$

and

$$\begin{aligned} \Vert f\Vert _{W^{1,p}_\mu }=\Vert f\Vert _{L^p_\mu }+\Vert \nabla f\Vert _{L^p_\mu } \end{aligned}$$

with the usual interpretation if $p=\infty$. If $\mu =0$ we simply write $L^p=L^p_0$ and $W^{1,p}=W^{1,p}_0$.

Along the paper we shall make extensively use of the Littlewood-Paley multipliers $\Delta _N$, namely

2.1 $$\begin{aligned} \hbox {Id}=\sum _{{N-\textrm{dyadic}}} \Delta _N \end{aligned}$$

where

$$\begin{aligned} \Delta _N f=N^2\int _{\mathbb R^2} K(N(x-y))f(y) dy, \quad N\ge 1 \end{aligned}$$

with $\hat{K}\in \mathcal S(\mathbb R^2)$ such that $\textrm{supp}\,\hat{K}(\xi )\subset \{\frac{1}{2} \le |\xi |\le 2\}$ and

$$\begin{aligned} \Delta _\frac{1}{2} f=\int _{\mathbb R^2} L(x-y)f(y) dy \end{aligned}$$

with $\hat{L}\in \mathcal S(\mathbb R^2)$ such that $\textrm{supp}\,\hat{L}(\xi )\subset \{|\xi |<1\}$. We also denote $\Delta _M = 0$ if $M < \frac{1}{2}$.

We can then introduce the weighted inhomogeneous Besov spaces $\mathcal {B}^\alpha _{p,q,\mu }$ as follows:

2.2 $$\begin{aligned} \Vert f\Vert _{\mathcal {B}^\alpha _{p,q,\mu }} = \Big (\sum _{{N-\textrm{dyadic}}} N^{\alpha q} \Vert \Delta _N f\Vert _{L^p_\mu }^q\Big )^\frac{1}{q} \end{aligned}$$

for every $\alpha , \mu \in \mathbb R$, $p, q\in [1, \infty ]$. Notice that for $\mu =0$ the space $\mathcal {B}^\alpha _{p,q,0}$ reduces to the usual Besov space $\mathcal {B}^\alpha _{p,q}$. A convenient property of the spaces $\mathcal {B}^\alpha _{p,q,\mu }$ is that the weight can be “pulled in”, namely we have the equivalent norms:

2.3 $$\begin{aligned} c \Vert f \langle x\rangle ^\mu \Vert _{\mathcal {B}^{\alpha }_{p,q}}<\Vert f\Vert _{\mathcal {B}^{\alpha }_{p,q,\mu }} \le C \Vert f \langle x\rangle ^\mu \Vert _{\mathcal {B}^{\alpha }_{p,q}}\,, \end{aligned}$$

for suitable $c, C>0$ that depend on $\alpha ,\,\mu \in \mathbb R$ and $p,q\in [1,\infty ]$ (see [40, Theorem 6.5]). Relation (2.3) can be used to translate results from the unweighted spaces to their weighted analogues.

In the case $(p,q)=(2,2)$, the weighted Besov spaces are generalizations of weighted Sobolev spaces:

2.4 $$\begin{aligned} \mathcal {B}^{\alpha }_{2,2, \mu } =H^\alpha _\mu , \quad \hbox { where } \Vert f\Vert _{H^\alpha _\mu } =\Vert \mathcal {F}^{-1}\langle \cdot \rangle ^\alpha \mathcal {F}f\Vert _{L^2_\mu }. \end{aligned}$$

In the sequel we shall make extensively use of the following obvious continuous embedding

2.5 $$\begin{aligned} H^{s_1}_{\mu _1}\subset H^{s_2}_{\mu _2}, \quad s_1\ge s_2, \quad \mu _1\ge \mu _2. \end{aligned}$$

The embedding (2.5) is compact when $s_1 > s_2$ and $\mu _1 > \mu _2$ (see [15, Section 4.2.3]). We shall also use the notation

2.6 $$\begin{aligned} \mathcal {B}^{\alpha }_{\infty ,\infty , \mu } =\mathcal {C}^{\alpha }_\mu . \end{aligned}$$

In the special case $\alpha \in \mathbb R_{+}\backslash \{0,1,2,\ldots \}$, the space $\mathcal {C}^\alpha _\mu$ is in turn equivalent to the classical weighted Hölder-Zygmund space with the following norm

2.7 $$\begin{aligned} \begin{aligned} \Vert f\Vert _{\mathcal {C}^\alpha _\mu }&\,=\sum _{|k|\le \lfloor \alpha \rfloor }\sup _{x\in \mathbb R^2} \,\langle x\rangle ^{\mu }\,|\partial ^k f(x)| \\&\qquad \quad \,+ \sum _{|k|=\lfloor \alpha \rfloor }\sup _{\begin{array}{c} x,y\in \mathbb R^2\\ 0<|x-y|\le 1 \end{array}}\langle x\rangle ^\mu \frac{|\partial ^k f(x)-\partial ^k f(y)|}{|x-y|^{\alpha -\lfloor \alpha \rfloor }}. \end{aligned} \end{aligned}$$

Setting $\mu = 0$ and restricting (2.7) to $x, y \in D$ for some domain $D \subset \mathbb R^2$ gives rise to the local Hölder space $\mathcal {C}^{\alpha } (D)$. For the other values of $\alpha$ (in particular for $\alpha <0$) we take (2.6) as a definition of $\mathcal {C}^\alpha _\mu$.

### Some properties of the Littlewood-Paley localization

We now gather well–known properties of the weighted Besov spaces that will be used along the paper. We first give an elementary, but useful, property of the Littlewood-Paley decomposition in weighted spaces.

#### Lemma 2.1

Let $\gamma , \delta \ge 0$ and $\gamma _0>0$ be given. Then, there exists $C>0$ such that

2.8 $$\begin{aligned} \sum _{{N-\textrm{dyadic}}} N^\gamma \Vert \Delta _N f\Vert _{L^2_\delta }\le C \Vert f\Vert _{H^{\gamma +\gamma _0}_{\delta }}. \end{aligned}$$

#### Proof

We have by the Cauchy-Schwarz inequality

2.9 $$\begin{aligned} \sum _{{N-\textrm{dyadic}}} N^\gamma \Vert \Delta _N f\Vert _{L^2_\delta }\le C \Big (\sum _{{N-\textrm{dyadic}}} N^{2(\gamma +\gamma _0)} \Vert \Delta _N f\Vert _{L^2_\delta }^2\Big )^\frac{1}{2} \end{aligned}$$

and hence we conclude by recalling (2.2) and (2.4). $\square$

We shall need the following commutator estimates.

#### Lemma 2.2

For every $\delta \in (0,1)$ and $p\in [1,\infty ]$, there exists $C>0$ such that for every *N* dyadic, we have

2.10 $$\begin{aligned}{} & {} \Vert [\Delta _N, \langle x\rangle ^{\delta }] f\Vert _{L^p}\le CN^{-1} \Vert f\Vert _{L^p}, \end{aligned}$$

2.11 $$\begin{aligned}{} & {} \Vert [\nabla , [\Delta _N,\langle x\rangle ^\delta ]]f\Vert _{L^p}\le CN^{-1}\Vert f\Vert _{L^p}. \end{aligned}$$

#### Proof

It is easy to check that

$$\begin{aligned}{}[\Delta _N, \langle x\rangle ^{\delta }] f= N^2 \int _{\mathbb R^2} (\langle y \rangle ^\delta -\langle x\rangle ^\delta ) K(N(x-y))f(y) dy \end{aligned}$$

and hence we easily conclude (2.10) by the Schur test since

$$\begin{aligned} \sup _x&\Big (N^2 \int _{\mathbb R^2} |\langle y \rangle ^\delta -\langle x\rangle ^\delta | |K(N(x-y))|dy\Big )\\&=\sup _y \Big ( N^2 \int _{\mathbb R^2} |\langle y \rangle ^\delta -\langle x\rangle ^\delta | |K(N(x-y))|dx\Big )\\&\le C N^{-1}\Vert |x|K(x)\Vert _{L^1} \end{aligned}$$

where we used that $|\langle y \rangle ^\delta -\langle x\rangle ^\delta |\le C|x-y|$ for $\delta <1$.

Concerning (2.11) we denote by $\tilde{K}_N(x,y)=N^2[\langle y \rangle ^\delta -\langle x\rangle ^\delta ] K(N(x-y))$ the kernel of the operator $[\Delta _N, \langle x\rangle ^{\delta }]$. Hence, we have

$$\begin{aligned}{}[\nabla , [\Delta _N,\langle x\rangle ^\delta ]]f=\int _{\mathbb R^2} (\nabla _x \tilde{K}_N(x,y)+ \nabla _y \tilde{K}_N(x,y))f(y) dy \end{aligned}$$

and we conclude as above via the Schuur test since

$$\begin{aligned} \nabla _x \tilde{K}_N(x,y)+ \nabla _y \tilde{K}_N(x,y)=N^2[-\nabla _x \langle x\rangle ^\delta + \nabla _y \langle y \rangle ^\delta ]K(N(x-y)) \end{aligned}$$

and $$|-\nabla _x \langle x\rangle ^\delta + \nabla _y \langle y\rangle ^\delta | \le C |x-y|$$. $$\square $$

Next, we show a useful property of the Littlewood-Paley multipliers $\Delta _N$ in weighted Sobolev spaces.

#### Lemma 2.3

For every $s\in [0,2]$ and $\delta \in (0, 1)$, there exists $C>0$ such that for every *N* dyadic, we have

2.12 $$\begin{aligned} \Vert \Delta _N f\Vert _{H^s_\delta }\le C N^{s}\Big (\Vert \Delta _\frac{N}{2} f\Vert _{L^{2}_\delta }+ \Vert \Delta _N f\Vert _{L^{2}_\delta } +\Vert \Delta _{2N} f\Vert _{L^{2}_\delta }\Big ). \end{aligned}$$

#### Proof

We split the proof in several cases.

**Case 1:** $$s\in [0,1)$$.

By combining (2.3) and (2.10) we get:

2.13 $$\begin{aligned} \begin{aligned} \Vert \Delta _N f\Vert _{H^s_\delta }^2&\le C\sum _{{M-\textrm{dyadic}}} M^{2s} \Vert \Delta _M (\langle x\rangle ^\delta \Delta _N f)\Vert _{L^2}^2 \\&\le C \sum _{{M-\textrm{dyadic}}} M^{2s} \Vert \langle x\rangle ^\delta \Delta _M (\Delta _N f)\Vert _{L^2}^2 \\&\quad + C \sum _{{M-\textrm{dyadic}}} M^{2s-2} \Vert \Delta _N f\Vert _{L^2}^2 \\&\le C \sum _{M=\frac{N}{2}, N, 2N} M^{2s} \Vert \langle x\rangle ^\delta \Delta _M f\Vert _{L^2}^2 + C \Vert \Delta _N f\Vert _{L^2}^2, \end{aligned} \end{aligned}$$

where we used that $\sum _{M-\textrm{dyadic}} M^{2s-2} <\infty$ for $s\in [0,1)$. Hence, we get

2.14 $$\begin{aligned} \begin{aligned} \Vert \Delta _N f\Vert _{H^s_\delta }^2&\le C N^{2s}\Big (\Vert \Delta _\frac{N}{2} f\Vert _{L^2_\delta }^2 + \Vert \Delta _N f\Vert _{L^2_\delta }^2+\Vert \Delta _{2N} f\Vert _{L^2_\delta }^2\Big ) \\&\quad + C N^{-2s}\Vert \Delta _N f\Vert _{H^s_\delta }^2 \end{aligned} \end{aligned}$$

From (2.14), we can deduce (2.12) provided that $N>\bar{N}$, with $\bar{N}$ choosen in such a way that the last term on the r.h.s. can be absorbed on the l.h.s. On the other hand, we have finitely dyadic numbers $1\le N\le \bar{N}$ and hence the corresponding estimate (2.12) for those values of *N* holds, provided that we choose the multiplicative constant large enough on the r.h.s.

**Case 2:** $$s\in [1, 2)$$.

We start by proving that for $s\in [1, 2]$, there exists $C>0$ such that for every *N* dyadic:

2.15 $$\begin{aligned} \Vert \Delta _N f\Vert _{H^s_\delta } \le C\Big ( \Vert \Delta _N f\Vert _{H^{s-1}_\delta }+ \Vert \Delta _N \nabla f\Vert _{H^{s-1}_\delta }\Big ).\end{aligned}$$

In fact, by (2.3) we have

2.16 $$\begin{aligned} \begin{aligned} \Vert \Delta _N f\Vert _{H^s_\delta }&\le C \Vert \langle x\rangle ^\delta \Delta _N f\Vert _{H^s} \\&\le C \Big (\Vert \langle x\rangle ^\delta \Delta _N f\Vert _{L^2} + \Vert \nabla (\langle x\rangle ^\delta \Delta _N f)\Vert _{H^{s-1}}\Big ) \\&\le C\Big (\Vert \langle x\rangle ^\delta \Delta _N f\Vert _{L^2} +\Vert \langle x\rangle ^\delta (\Delta _N \nabla f)\Vert _{H^{s-1}}\\&\quad + \Vert [\nabla , \langle x\rangle ^\delta ] \Delta _N f\Vert _{H^{s-1}}\Big ) \\&\le C\Big (\Vert \langle x\rangle ^\delta \Delta _N f\Vert _{L^2} + \Vert \langle x\rangle ^\delta (\Delta _N \nabla f)\Vert _{H^{s-1}}+ \Vert \Delta _N f\Vert _{H^{s-1}}\Big ). \end{aligned} \end{aligned}$$

Here, we used the elementary commutator bound

2.17 $$\begin{aligned} \Vert [\nabla , \langle x\rangle ^\delta ] f\Vert _{H^\sigma }\le C \Vert f\Vert _{H^\sigma }, \quad \sigma \in [0,1], \end{aligned}$$

which follows from interpolating the $L^2 \rightarrow L^2$ bound and the $H^1 \rightarrow H^1$ bound of the commutator $[\nabla , \langle x\rangle ^\delta ]$.

Next, we show (2.12) for $s\in [1,2)$. Notice that we have $s-1\in [0,1)$ and hence we can combine (2.15) with the estimate proved in the first case in order to obtain

2.18 $$\begin{aligned} \begin{aligned} \Vert \Delta _N f\Vert _{H^s_\delta }&\le C N^{s-1}\Big (\Vert \Delta _\frac{N}{2} f\Vert _{L^{2}_\delta }+ \Vert \Delta _N f\Vert _{L^{2}_\delta } +\Vert \Delta _{2N} f\Vert _{L^{2}_\delta }\Big ) \\&\quad + C N^{s-1}\Big (\Vert \Delta _\frac{N}{2} \nabla f\Vert _{L^2_\delta }+ \Vert \Delta _N \nabla f\Vert _{L^2_\delta } +\Vert \Delta _{2N} \nabla f\Vert _{L^{2}_\delta }\Big ). \end{aligned} \end{aligned}$$

Then, we get for a generic dyadic *M*

$$\begin{aligned} \Vert \Delta _M \nabla f\Vert _{L^2_\delta } \le C\Big ( \Vert \nabla (\langle x\rangle ^\delta \Delta _M f)\Vert _{L^2}+ \Vert [\langle x\rangle ^\delta , \nabla ] \Delta _M f\Vert _{L^2}\Big ) \end{aligned}$$

and by noticing that $(\Delta _{\frac{M}{2}} + \Delta _M + \Delta _{2\,M}) \circ \Delta _M = \Delta _M$ and using (2.17), we get

$$\begin{aligned} \dots&\le C \sum _{M' = \frac{M}{2}, M, 2M} \Big ( \Vert \nabla \Delta _{M'} (\langle x\rangle ^\delta \Delta _M f)\Vert _{L^2} \\&\qquad \qquad +\Vert \nabla ( [\Delta _{M'},\langle x\rangle ^\delta ] \Delta _M f)\Vert _{L^2} + \Vert \Delta _M f\Vert _{L^2}\Big ) \\&\le C \sum _{M' = \frac{M}{2}, M, 2M} \Big (M\Vert \Delta _M f\Vert _{L^2_\delta }+\Vert [\Delta _{M'},\langle x\rangle ^\delta ] \Delta _M \nabla f\Vert _{L^2} \\&\qquad \qquad + \Vert [\nabla , [\Delta _{M'},\langle x\rangle ^\delta ]] \Delta _M f\Vert _{L^2}\Big ). \end{aligned}$$

By using (2.10) and (2.11), we can summarize the estimates above as follows

2.19 $$\begin{aligned} \Vert \Delta _M \nabla f\Vert _{L^2_\delta }\le C M\Vert \Delta _M f\Vert _{L^2_\delta }. \end{aligned}$$

We get the desired conclusion by combining (2.18) and (2.19).

**Case 3:** $$s=2$$.

We use (2.15) for $s=2$ and the fact that (2.12) has been proved for $s=1$ to obtain

$$\begin{aligned} \Vert \Delta _N f\Vert _{H^2_\delta }&\le C\Big ( \Vert \Delta _N f\Vert _{H^{1}_\delta }+ \Vert \Delta _N \nabla f\Vert _{H^{1}_\delta }\Big ) \\&\le C N\Big (\Vert \Delta _\frac{N}{2} f\Vert _{L^{2}_\delta }+ \Vert \Delta _N f\Vert _{L^{2}_\delta } +\Vert \Delta _{2N} f\Vert _{L^{2}_\delta }\Big ) \\&\quad +C N\Big (\Vert \Delta _\frac{N}{2} \nabla f\Vert _{L^2_\delta }+ \Vert \Delta _N \nabla f\Vert _{L^2_\delta } +\Vert \Delta _{2N} \nabla f\Vert _{L^{2}_\delta }\Big ). \end{aligned}$$

We conclude by using (2.19). $\square$

We shall also use the following estimates.

#### Lemma 2.4

For every $s\in (0,1)$, $s_0 \in \mathbb R$, and $\delta >0$, there exists $C>0$ such that for every *N* dyadic, we have

2.20 $$\begin{aligned}{} & {} \Vert \Delta _N f\Vert _{H^{s}_\delta }\le C N^{s_0} \sum _{\frac{N}{4} \le M \le 4N} \Vert \Delta _M f\Vert _{H^{s-s_0}_\delta }, \end{aligned}$$

2.21 $$\begin{aligned}{} & {} \Vert \Delta _N f\Vert _{H^{1+s}_\delta }\le C N^{s_0} \sum _{\frac{N}{4} \le M \le 4N} \Vert \Delta _M f\Vert _{H^{1+s-s_0}_\delta }. \end{aligned}$$

#### Proof

We first prove (2.20) and notice that by (2.13)

$$\begin{aligned} \Vert \Delta _N f\Vert _{H^s_\delta }^2 \le C \sum _{M=\frac{N}{2}, N, 2N} M^{2s} \Vert \langle x\rangle ^\delta \Delta _M f\Vert _{L^2}^2 + C \Vert \Delta _N f\Vert _{L^2}^2 \end{aligned}$$

that can be continued as follows

$$\begin{aligned} \dots&\le C N^{2s_0}\sum _{M=\frac{N}{2}, N, 2N} M^{2s-2s_0} \Vert \langle x\rangle ^\delta \Delta _M f\Vert _{L^2}^2 + C \Vert \Delta _N f\Vert _{L^2}^2 \\&\le C N^{2s_0} \sum _{M' = \frac{M}{2}, M, 2M} \sum _{M=\frac{N}{2}, N, 2N} M^{2s-2s_0} \Vert \Delta _M(\langle x\rangle ^\delta \Delta _{M'} f)\Vert _{L^2}^2 \\&\quad +C N^{2s_0} \sum _{M' = \frac{M}{2}, M, 2M} \sum _{M=\frac{N}{2}, N, 2N} M^{2s-2s_0-2} \Vert \Delta _{M'} f\Vert _{L^2}^2 + C \Vert \Delta _N f\Vert _{L^2}^2, \end{aligned}$$

where we used the estimate (2.10) and the fact that $(\Delta _{\frac{M}{2}} + \Delta _M + \Delta _{2\,M}) \circ \Delta _M = \Delta _M$. The proof follows since exactly as in the proof of (2.12), the term $\Vert \Delta _N f\Vert _{L^2}$ on the r.h.s. can be absorbed by $\Vert \Delta _N f\Vert _{H^s_\delta }^2$.

Next, we focus on (2.21) and we recall that by (2.15)

2.22 $$\begin{aligned} \Vert \Delta _N f\Vert _{H^{1+s}_\delta } \le C\Big ( \Vert \Delta _N f\Vert _{H^{s}_\delta }+ \Vert \Delta _N \nabla f\Vert _{H^{s}_\delta }\Big ). \end{aligned}$$

Arguing as above, we get

$$\begin{aligned} \Vert \Delta _N \psi \Vert _{H^s_\delta }^2 \le C N^{2s_0}\sum _{\frac{N}{4} \le M \le 4N} \Vert \Delta _M\psi \Vert _{H^{s-s_0}_\delta }^2 + C \Vert \Delta _N \psi \Vert _{L^2}^2 \end{aligned}$$

and by choosing $\psi =f$ and $\psi = \nabla f$, we get from (2.22)

$$\begin{aligned} \Vert \Delta _N f\Vert _{H^{1+s}_\delta }^2&\le C N^{2s_0}\sum _{\frac{N}{4} \le M \le 4N} \Vert \Delta _M f\Vert _{H^{s-s_0}_\delta }^2 + C \Vert \Delta _N f\Vert _{L^2}^2 \\&\quad +C N^{2s_0}\sum _{\frac{N}{4} \le M \le 4N} \Vert \Delta _M\nabla f\Vert _{H^{s-s_0}_\delta }^2 + C \Vert \Delta _N \nabla f\Vert _{L^2}^2. \end{aligned}$$

As in the proof of (2.12), we can absorb $\Vert \Delta _N f\Vert _{L^2}^2$ and $\Vert \Delta _N \nabla f\Vert _{L^2}^2$ on the l.h.s. and hence we obtain

$$\begin{aligned} \Vert \Delta _N f\Vert _{H^{1+s}_\delta }^2&\le C N^{2s_0}\sum _{\frac{N}{4} \le M \le 4N} \Vert \Delta _M f\Vert _{H^{s-s_0}_\delta }^2 \\&\quad +C N^{2s_0}\sum _{\frac{N}{4} \le M \le 4N} \Vert \Delta _M\nabla f\Vert _{H^{s-s_0}_\delta }^2 \\&\le C N^{2s_0}\sum _{\frac{N}{4} \le M \le 4N} \Vert \Delta _M f\Vert _{H^{s-s_0}_\delta }^2 +C N^{2s} \sum _{\frac{N}{4} \le M \le 4N} \Vert \Delta _M \nabla f\Vert _{L^2_\delta }^2 \\&\le C N^{2s_0}\sum _{\frac{N}{4} \le M \le 4N} \Vert \Delta _M f\Vert _{H^{s-s_0}_\delta }^2 \\&\quad +C N^{2s_0} \sum _{\frac{N}{4} \le M \le 4N} M^{2+2s-2s_0}\Vert \Delta _M f\Vert _{L^2_\delta }^2, \end{aligned}$$

where we used (2.19). The conclusion follows. $\square$

We close this subsection with the following result.

#### Lemma 2.5

For every $\beta ,\gamma \in \mathbb R$, $\delta \ge 0$, and $\varphi \in C^\infty _c(\mathbb R^2)$, there exists $C>0$ such that

2.23 $$\begin{aligned} \Vert \varphi *f \Vert _{\mathcal {C}^{\beta }_{-\delta }} \le C\Vert f\Vert _{\mathcal {C}^{\gamma }_{-\delta }}. \end{aligned}$$

#### Proof

In [2, Lemma 2.2] this estimate is proved for $\delta =0$. For $\delta >0$ we proceed as follows. Since $(\Delta _{\frac{N}{2}} + \Delta _N + \Delta _{2N}) \circ \Delta _N = \Delta _N$, we get

$$\begin{aligned} N^\beta \Vert \Delta _N (\varphi *f) \Vert _{L^\infty _{-\delta }}&= N^\beta \sum _{M = \frac{N}{2}, N, 2N} \Vert \Delta _M \varphi *\Delta _N f\Vert _{L^\infty _{-\delta }} \\&\le N^\beta \Vert \Delta _N f\Vert _{L^\infty _{-\delta }} \sum _{M = \frac{N}{2}, N, 2N} \Vert \Delta _M \varphi \Vert _{L^1_\delta } \\&\le N^\gamma \Vert \Delta _N f\Vert _{L^\infty _{-\delta }} \sum _{M = \frac{N}{2}, N, 2N} M^{\beta -\gamma } \Vert \Delta _M \varphi \Vert _{L^\infty _{2 + 2 \delta }}, \end{aligned}$$

where we used $\langle x\rangle ^{-1}\le 2\langle y\rangle \langle x-y\rangle ^{-1}$ and the Hölder inequality. We conclude since $\varphi \in \mathcal {C}_{2 + 2 \delta }^{\beta -\gamma }$. $\square$

### Some estimates on the approximation of the identity $\rho _\varepsilon$

We shall need the following estimate proved in [4] and in a different case, but with a similar proof, in [5, Lemma 8].

#### Lemma 2.6

For every $\delta >0$, there exists $C>0$ such that:

2.24 $$\begin{aligned} \Vert \rho _\varepsilon \Vert _{\mathcal {B}^0_{1,2,\delta }} \le C \sqrt{ |\ln \varepsilon |}, \quad \forall \varepsilon \in \Big (0, \frac{1}{2}\Big ). \end{aligned}$$

#### Proof

We give the proof for the sake of completeness. First we notice that

$$\begin{aligned} \Delta _N (\langle x\rangle ^\delta \rho _{\varepsilon }) (\varepsilon x)=N^2 \int _{\mathbb R^2} K(\varepsilon N(x-y)) \langle \varepsilon y\rangle ^\delta \rho (y) dy \end{aligned}$$

and hence it is easy to deduce

2.25 $$\begin{aligned} \begin{aligned} \varepsilon ^{-2}\Vert \Delta _N (\langle x\rangle ^\delta \rho _{\varepsilon })\Vert _{L^1}&\le C N^2\Vert K(\varepsilon Nx)\Vert _{L^1}\Vert \langle \varepsilon x\rangle ^\delta \rho \Vert _{L^1} \\&=C\varepsilon ^{-2} \Vert K\Vert _{L^1} \Vert \langle \varepsilon x\rangle ^\delta \rho \Vert _{L^1} \\&\le C\varepsilon ^{-2} \Vert K\Vert _{L^1} \Vert \rho \Vert _{L^1_\delta }. \end{aligned} \end{aligned}$$

We fix $N_0$ as the unique dyadic such that $N_0\le \frac{1}{\varepsilon }<2N_0$ and from (2.25) we get

2.26 $$\begin{aligned} \sum _{\frac{1}{2}\le N\le 8N_0} \Vert \Delta _N (\langle x\rangle ^\delta \rho _{\varepsilon })\Vert _{L^1}^2\le C |\ln \varepsilon |. \end{aligned}$$

Next, we focus on the case $N>8N_0$. First notice the identity

2.27 $$\begin{aligned} \varepsilon ^2 \langle x\rangle ^\delta \rho _{\varepsilon }=\sum _{{M-\textrm{dyadic}}} \Delta _M (\langle \varepsilon x\rangle ^\delta \rho ) \Big (\frac{x}{\varepsilon }\Big ), \end{aligned}$$

along with the following inclusion for the support of the Fourier transfom

$$\begin{aligned} \textrm{supp}\,\mathcal {F} \Big [\Delta _M (\langle \varepsilon x\rangle ^\delta \rho )\Big (\frac{x}{\varepsilon }\Big )\Big ]\subset \Big \{\frac{M}{2 \varepsilon }\le |\eta |\le \frac{2M}{\varepsilon }\Big \}\subset \Big \{\frac{MN_0}{2}\le |\eta |\le 4MN_0\Big \}. \end{aligned}$$

As a consequence, we get

$$\begin{aligned} \Delta _N \Big [\Delta _M (\langle \varepsilon x\rangle ^\delta \rho ) \Big (\frac{x}{\varepsilon }\Big )\Big ]=0, \quad \forall N \hbox { s.t. } \frac{N}{2} >4MN_0 \hbox { or } 2N<\frac{MN_0}{2}. \end{aligned}$$

Hence, by (2.27) we get

2.28 $$\begin{aligned} \varepsilon ^2 \Delta _N (\langle x\rangle ^\delta \rho _{\varepsilon })=\sum _{\frac{N}{8N_0}\le M\le \frac{4N}{N_0}} \Delta _N\Big [\Delta _M (\langle \varepsilon x\rangle ^\delta \rho ) \Big (\frac{x}{\varepsilon }\Big )\Big ], \quad \forall N\ge 8N_0, \end{aligned}$$

which in turn implies (since there are exactly six dyadic numbers in the interval $[\frac{N}{8N_0}, \frac{4N}{N_0}]$ )

$$\begin{aligned} \Vert \Delta _N (\langle x\rangle ^\delta \rho _{\varepsilon })\Vert _{L^1}^2&\le \Big (\sum _{\frac{N}{8N_0}\le M\le \frac{4N}{N_0}} \varepsilon ^{-2}\Big \Vert \Delta _M (\langle \varepsilon x\rangle ^\delta \rho ) \Big (\frac{x}{\varepsilon }\Big )\Big \Vert _{L^1}\Big )^2\\&\le 6 \sum _{\frac{N}{8N_0}\le M\le \frac{4N}{N_0}}\Vert \Delta _M (\langle \varepsilon x\rangle ^\delta \rho )\Vert _{L^1}^2, \quad \forall N\ge 8N_0 \end{aligned}$$

and then

2.29 $$\begin{aligned} \sum _{N>8 N_0} \Vert \Delta _N (\langle x\rangle ^\delta \rho _{\varepsilon })\Vert _{L^1}^2&\le 36 \sum _{{M-\textrm{dyadic}}} \Vert \Delta _M (\langle \varepsilon x\rangle ^\delta \rho )\Vert _{L^1}^2 \le C \Vert \langle \varepsilon x \rangle ^\delta \rho \Vert _{\mathcal {B}_{1, 2}^0}^2. \end{aligned}$$

By combining (2.26), (2.29), and the obvious bound $\sup _{\varepsilon (0, \frac{1}{2})} \Vert \langle \varepsilon x \rangle ^\delta \rho \Vert _{\mathcal {B}_{1, 2}^0} < \infty$, we conclude our estimate. $\square$

The following estimate will also be useful.

#### Lemma 2.7

For every $\beta \in (0,1)$, $\zeta >0$ with $\beta +\zeta \in (0,1)$, and $\delta \ge 0$, there exists $C>0$ such that

2.30 $$\begin{aligned} \Vert \rho _\varepsilon *f\Vert _{\mathcal {C}^{\beta +1}_{-\delta }}\le C\varepsilon ^{-\beta -\zeta }\Vert f\Vert _{\mathcal {C}^{1-\zeta }_{-\delta }}, \quad \forall \varepsilon \in (0,1). \end{aligned}$$

#### Proof

Arguing as in the proof of (2.23), we get

$$\begin{aligned} N^{1+\beta } \Vert \Delta _N (\rho _\varepsilon *f) \Vert _{L^\infty _{-\delta }} \le N^{1-\zeta } \Vert \Delta _N f\Vert _{L^\infty _{-\delta }} \sum _{M = \frac{N}{2}, N, 2N} M^{\beta +\zeta } \Vert \Delta _M \rho _\varepsilon \Vert _{L^1_\delta } \end{aligned}$$

and hence we conclude provided that we show

2.31 $$\begin{aligned} (\varepsilon N)^{\beta +\zeta } \Vert \Delta _N \rho _\varepsilon \Vert _{L^1_\delta }\le C, \quad \forall \varepsilon \in (0, 1), \quad \forall {N\hbox { } \textrm{dyadic}}. \end{aligned}$$

By using (2.10), and since we are assuming $\beta +\zeta \in (0,1)$, it is sufficient to prove

2.32 $$\begin{aligned} (\varepsilon N)^{\beta +\zeta } \Vert \Delta _N (\langle x\rangle ^{\delta } \rho _\varepsilon )\Vert _{L^1}\le C, \quad \forall \varepsilon \in (0, 1), \quad \forall {N\hbox { } \textrm{dyadic}}. \end{aligned}$$

In order to do that we introduce the unique dyadic $N_0$ such that $N_0\le \frac{1}{\varepsilon }<2N_0$. Notice that in case $\frac{N}{N_0}\le 8$ the estimate above is trivial. On the other hand from (2.28), we get

$$\begin{aligned} \Vert \Delta _N (\langle x \rangle ^\delta \rho _\varepsilon ) \Vert _{L^1} \le \sum _{\frac{N}{8N_0}\le M\le \frac{4N}{N_0}} \Vert \Delta _N (\langle \varepsilon x \rangle ^\delta \rho ) \Vert _{L^1}, \quad \forall N > 8 N_0, \end{aligned}$$

and hence

$$\begin{aligned} \Big ( \frac{N}{N_0} \Big )^{\beta + \zeta } \Vert \Delta _N (\langle x \rangle ^\delta \rho _\varepsilon ) \Vert _{L^1} \le C \sup _{\varepsilon \in (0, 1)} \Vert \langle \varepsilon x \rangle ^\delta \rho \Vert _{\mathcal {B}_{1, \infty }^{\beta + \zeta }}, \quad \forall N > 8 N_0. \end{aligned}$$

We conclude since $\sup _{\varepsilon \in (0, 1)} \Vert \langle \varepsilon x \rangle ^\delta \rho \Vert _{\mathcal {B}_{1, \infty }^{\beta + \zeta }} < \infty$. $\square$

We close the subsection with the following result.

#### Lemma 2.8

For every $\alpha \in \mathbb R$, $\eta \in (0,1)$, and $\delta \ge 0$, there exists $C>0$ such that

2.33 $$\begin{aligned} \Vert \rho _\varepsilon *f-f \Vert _{\mathcal {C}^\alpha _{-\delta }}\le C\varepsilon ^\eta \Vert f\Vert _{\mathcal {C}^{\alpha +\eta }_{-\delta }}, \quad \forall \varepsilon \in (0, 1). \end{aligned}$$

#### Proof

For $\varepsilon N \ge 1$, we have the bound

2.34 $$\begin{aligned} \begin{aligned} N^\alpha \Vert \Delta _N (\rho _\varepsilon * f)-\Delta _N f\Vert _{L^\infty _{-\delta }}&\le N^\alpha \Vert \rho _\varepsilon * \Delta _N f\Vert _{L^\infty _{-\delta }}+N^\alpha \Vert \Delta _N f\Vert _{L^\infty _{-\delta }} \\&\le N^\alpha \Vert \rho _\varepsilon \Vert _{L^1_{\delta }} \Vert \Delta _N f\Vert _{L^\infty _{-\delta }}+N^\alpha \Vert \Delta _N f\Vert _{L^\infty _{-\delta }} \\&\le C N^{\alpha +\eta } \varepsilon ^{\eta }\Vert \Delta _N f\Vert _{L^\infty _{-\delta }}, \end{aligned} \end{aligned}$$

where we used $\langle x\rangle ^{-1}\le 2\langle y\rangle \langle x-y\rangle ^{-1}$. To deal with the case $\varepsilon N<1$, we notice that

$$\begin{aligned} \langle x\rangle ^{-\delta } \Big (\Delta _N (\rho _\varepsilon * f)-\Delta _N f\Big )=\int _{\mathbb R^2} \tilde{K}_{\varepsilon ,N} (x,y) \langle y \rangle ^{-\delta } f(y) dy \end{aligned}$$

where

$$\begin{aligned} \tilde{K}_{\varepsilon ,N} (x,y)&= N^2\frac{\langle y \rangle ^\delta }{\langle x\rangle ^\delta }\Big (\int _{\mathbb R^2} K(Nx-Nz)\rho _\varepsilon (z-y)dz-K(Nx-Ny)\Big ) \\&= N^2 \frac{\langle y \rangle ^\delta }{\langle x\rangle ^\delta }\int _{\mathbb R^2}\Big (K(Nx-Nz)-K(Nx-Ny)\Big )\rho _\varepsilon (z-y)dz. \end{aligned}$$

Next, fox fixed *x*, we compute

2.35 $$\begin{aligned}{} & {} \begin{aligned}&\int _{\mathbb R^2} |\tilde{K}_{\varepsilon ,N} (x,y)|dy\\&\le \frac{N^2}{\langle x\rangle ^\delta } \int _{\mathbb R^2}\int _{\mathbb R^2} |K(Nx-Nz)-K(Nx-Ny)|\rho _\varepsilon (z-y) \langle y \rangle ^\delta dydz \\&=\frac{(N \varepsilon )^2}{\langle x\rangle ^\delta } \int _{\mathbb R^2}\int _{\mathbb R^2} |K(Nx-N\varepsilon z)-K(Nx-N\varepsilon y)|\rho (z-y)\langle \varepsilon y \rangle ^\delta dydz \\&\ \le \frac{(N\varepsilon )^3}{\langle x\rangle ^\delta } \int _{\mathbb R^2}\int _{\mathbb R^2} \Big ( \sup _{[Nx-N\varepsilon z,Nx-N\varepsilon y ]} |\nabla K|\Big ) |z-y| \rho (z-y) \langle \varepsilon y \rangle ^\delta dydz \end{aligned}\qquad \end{aligned}$$

and notice that for every $\bar{x}\in \mathbb R^2$ and $\lambda \in (0,1)$, we get

$$\begin{aligned} \int _{\mathbb R^2}&\int _{\mathbb R^2} \Big ( \sup _{[\bar{x}- \lambda z, \bar{x}-\lambda y ]} |\nabla K|\Big ) |z-y| \rho (z-y) \langle \varepsilon y \rangle ^\delta dydz \\&\le C\int _{\mathbb R^2} \int _{|z-y|<1} \Big ( \sup _{B((\bar{x}- \lambda z), \lambda )} |\nabla K|\Big ) \langle \varepsilon z \rangle ^\delta dzdy \\&\le C \int _{\mathbb R^2} \Big ( \sup _{B((\bar{x}- \lambda z), 1)} |\nabla K|\Big ) \langle \varepsilon z \rangle ^\delta dz. \end{aligned}$$

Here, we denoted by [*a*, *b*] the segment between *a* and *b*, *B*(*x*, *r*) the ball in $\mathbb R^2$ centered in *x* of radius *r* and we used the inclusion $[\bar{x}- \lambda z, \bar{x}-\lambda y ]\subset B((\bar{x}- \lambda z), 1)$ since $|z-y|<1$ and $\lambda \in (0,1)$. In particular, by introducing the function

2.36 $$\begin{aligned} G(w)=\sup _{B(w, 1)} |\nabla K|, \end{aligned}$$

we get from the estimate above

2.37 $$\begin{aligned} \begin{aligned} \int _{\mathbb R^2}&\int _{\mathbb R^2} \Big ( \sup _{[\bar{x}- \lambda z, \bar{x}-\lambda y ]} |\nabla K|\Big ) |z-y| \rho (z-y)\langle \varepsilon z \rangle ^\delta dydz \\&\le \int _{\mathbb R^2} G(\bar{x} - \lambda z) \langle \varepsilon z \rangle ^\delta dz \\&=\lambda ^{-2} \int _{\mathbb R^2} G(u) \langle \varepsilon \lambda ^{-1}(\bar{x} - u) \rangle ^\delta du. \end{aligned} \end{aligned}$$

By combining (2.35) with (2.37), where we choose $\lambda =\varepsilon N$ and $\bar{x}=Nx$, we conclude

2.38 $$\begin{aligned} \sup _{x,N} \int _{\mathbb R^2} |\tilde{K}_{\varepsilon ,N} (x,y)|dy=O(\varepsilon N), \end{aligned}$$

where we used

$$\begin{aligned} \sup _{\bar{x}, N} \int _{\mathbb R^2} G(u) \langle N^{-1}(\bar{x} - u) \rangle ^\delta du<\infty . \end{aligned}$$

Similarly, one can show

2.39 $$\begin{aligned} \sup _{y,N} \int _{\mathbb R^2} |\tilde{K}_{\varepsilon ,N} (x,y)|dx=O(\varepsilon N). \end{aligned}$$

In fact for *y* fixed we get:

$$\begin{aligned} \int _{\mathbb R^2}&|\tilde{K}_{\varepsilon ,N} (x,y)|dx \\&=N^2 \langle y \rangle ^\delta \int _{\mathbb R^2}\int _{\mathbb R^2} |K(Nx-N\varepsilon z-Ny)-K(Nx-N y)|\rho (z)\langle x \rangle ^{-\delta } dxdz \\ {}&\le C \varepsilon N^3 \langle y \rangle ^\delta \int _{\mathbb R^2}\int _{\mathbb R^2} \Big ( \sup _{[Nx-N\varepsilon z-Ny,Nx-N y ]} |\nabla K|\Big ) |z| \rho (z) \langle x \rangle ^{-\delta } dxdz \\ {}&\le C\varepsilon N^3 \langle y \rangle ^\delta \int _{\mathbb R^2}\int _{|z|<1} G(N(x-y)) \langle x \rangle ^{-\delta } dxdz, \end{aligned}$$

where the function *G*(*w*) is defined in (2.36) and we used the inclusion

$$[Nx-N\varepsilon z-Ny,Nx-N y ]\subset B((Nx-Ny), 1)$$

for $|z|<1$ and $\varepsilon N<1$. By a change of variable, we conclude

$$\begin{aligned} \int _{\mathbb R^2} |\tilde{K}_{\varepsilon ,N} (x,y)|dx\le C\varepsilon N \langle y \rangle ^\delta \int _{\mathbb R^2}G(x) \langle \frac{x}{N}+y \rangle ^{-\delta } dx \le C \varepsilon N \int _{\mathbb R^2} G(x) \langle x\rangle ^\delta dx. \end{aligned}$$

Summarizing by (2.38) and (2.39) we can apply the Schuur Lemma and we get

$$\begin{aligned} \Vert \Delta _N (\rho _\varepsilon * f)-\Delta _N f\Vert _{L^\infty _{-\delta }}\le C\varepsilon N \Vert \Delta _N f\Vert _{L^\infty _{-\delta }},\quad \varepsilon N<1, \end{aligned}$$

which in turn implies

$$\begin{aligned} \Vert \Delta _N (\rho _\varepsilon * f)-\Delta _N f\Vert _{L^\infty _{-\delta }}\le C\varepsilon ^\eta N^\eta \Vert \Delta _N f\Vert _{L^\infty _{-\delta }},\quad \varepsilon N<1. \end{aligned}$$

We conclude by combining this estimate with (2.34). $\square$

### Embeddings and product rules

We first record the useful interpolation inequality in weighted Besov spaces (see [36, Theorem 3.8])

#### Lemma 2.9

Let $p_0,q_0,p_1,q_1,p,q\in [1,\infty ],\,\alpha _0,\alpha _1,\mu _0,\mu _1, \alpha , \mu \in \mathbb R$ be such that

$$\begin{aligned} \frac{1}{p}&=\frac{1-\varTheta }{p_0}+\frac{\varTheta }{p_1},\quad \frac{1}{q}=\frac{1-\varTheta }{q_0}+\frac{\varTheta }{q_1},\\ \alpha&= (1-\varTheta )\alpha _0+\varTheta \alpha _1, \quad \mu =(1-\varTheta )\mu _0+\varTheta \mu _1 \end{aligned}$$

for a suitable $\varTheta \in [0,1]$. Then there exists $C>0$ such that

2.40 $$\begin{aligned} \Vert f\Vert _{\mathcal {B}^{\alpha }_{p,q,\mu }}\le C \Vert f\Vert ^{1-\varTheta }_{\mathcal {B}^{\alpha _0}_{p_0,q_0, \mu _0}} \Vert f\Vert ^\varTheta _{\mathcal {B}^{\alpha _1}_{p_1,q_1,\mu _1}}. \end{aligned}$$

Next, we state a Sobolev embedding for weighted Sobolev spaces. The proof follows from the corresponding unweighted version along with (2.3).

#### Lemma 2.10

Let $p\in [2,\infty )$, $\alpha \ge 1-\frac{2}{p}$, and $\mu ,\nu \in \mathbb R$ such that $\nu \le \mu$. Then, we have the continuous embedding

2.41 $$\begin{aligned} H^{\alpha }_{\mu } \subset L^p_{\nu }. \end{aligned}$$

Moreover for every $\alpha >1$ we have

2.42 $$\begin{aligned} H^{\alpha }_{\mu } \subset L^\infty _{\nu }. \end{aligned}$$

As a consequence, we can prove the following estimate.

#### Lemma 2.11

For every $q\in [2,\infty )$ and $\delta \in (0, 1]$, there exists $C>0$ such that

2.43 $$\begin{aligned} \Vert f\Vert _{W^{1,q}_{\delta }}\le C \Vert f\Vert _{H^2_{-\delta }}^{1-\frac{1}{q}} \Vert f\Vert _{L^2_{(2q-1)\delta }}^{\frac{1}{q}}. \end{aligned}$$

#### Proof

We have the following chain of estimates

$$\begin{aligned} \Vert f\Vert _{W^{1,q}_{\delta }}&\le C \Vert f\Vert _{H^{2-\frac{2}{q}}_{\delta }} \\&\le C \Vert f\Vert _{H^2_{-\delta }}^{1-\frac{2}{q}} \Vert f\Vert _{H^1_{(q-1)\delta }}^\frac{2}{q} \\&\le C \Vert f\Vert _{H^2_{-\delta }}^{1-\frac{2}{q}} \Vert f\Vert _{H^2_{-\delta }}^\frac{1}{q} \Vert f\Vert _{L^2_{(2q-1)\delta }}^{\frac{1}{q}} \end{aligned}$$

where the first inequality is a consequence of (2.17) and (2.41). Moreover we have also used twice special cases of (2.40). $\square$

We shall also need the following product estimate (see [2] or [35]).

#### Lemma 2.12

Let $\alpha _1, \alpha _2\in \mathbb R$ with $\alpha _1 + \alpha _2 > 0$, $\alpha = \min (\alpha _1, \alpha _2, \alpha _1 + \alpha _2)$, $\mu _1, \mu _2 \in \mathbb R$, $\mu = \mu _1 + \mu _2$, $p_1, p_2 \in [1, \infty ]$, and $\frac{1}{p} = \frac{1}{p_1} + \frac{1}{p_2}$. Then, for any $\kappa > 0$, we have

2.44 $$\begin{aligned} \Vert f_1 \cdot f_2 \Vert _{\mathcal {B}^{\alpha - \kappa }_{p, p, \mu }} \le C \Vert f_1 \Vert _{\mathcal {B}^{\alpha _1}_{p_1, p_1, \mu _1}} \Vert f_2 \Vert _{\mathcal {B}^{\alpha _2}_{p_2, p_2, \mu _2}}. \end{aligned}$$

Also, we have

2.45 $$\begin{aligned} \Vert f_1\cdot f_2\Vert _{\mathcal {C}^{\alpha }_{\mu }}\le C\Vert f_1\Vert _{\mathcal {C}^{\alpha _1}_{\mu _1}} \Vert f_2\Vert _{{\mathcal C}^{\alpha _2}_{\mu _2}}. \end{aligned}$$

The following duality estimate will also be useful (see [39, Theorem 2.11.2]).

#### Lemma 2.13

Let $\alpha , \mu \in \mathbb R$ and $p, q \in [1, \infty )$. Then, we have

2.46 $$\begin{aligned} \Big | \int _{\mathbb R^2} f_1 \cdot f_2 dx \Big | \le C \Vert f_1 \Vert _{\mathcal {B}^\alpha _{p, q, \mu }} \Vert f_2 \Vert _{\mathcal {B}^{-\alpha }_{p', q', - \mu }}, \end{aligned}$$

where $$\frac{1}{p} + \frac{1}{p'} = 1$$ and $$\frac{1}{q} + \frac{1}{q'} = 1$$.

## Stochastics bounds

The following results are improvements of some results from [20] and [12], where similar estimates were given in terms of moments. In this paper, these estimates hold almost surely.

### Estimates in classical spaces

We first show some estimates in classical spaces.

#### Proposition 3.1

Given $\delta \in (0,1)$, $r\in (2,\infty )$ such that $\delta \cdot r>2$, and $\varepsilon \in (0, \frac{1}{2})$, we have

3.1 $$\begin{aligned} \Vert \nabla Y_\varepsilon \Vert _{L^r_{-\delta }}^2+\Vert \mathbf {:}\nabla Y^2_\varepsilon \mathbf {:}\Vert _{L^r_{-\delta }}\le C(\omega ) |\ln \varepsilon |, \quad \hbox { a.s. } \omega . \end{aligned}$$

Moreover, for $\alpha \in (0,1),\,\delta >0, \; \beta \in \mathbb R$, $\varepsilon \in (0, 1)$, and $\varphi \in C_c^\infty (\mathbb R^2)$, there exists $\kappa \in (0,1)$ such that

3.2 $$\begin{aligned} \Vert Y_\varepsilon -Y\Vert _{\mathcal {C}^{\alpha }_{-\delta }} +\Vert \varphi *\xi _\varepsilon -\varphi *\xi \Vert _{\mathcal {C}^{\beta }_{-\delta }} \le C(\omega ) \varepsilon ^{\kappa }, \quad \hbox { a.s. } \omega . \end{aligned}$$

To prove Proposition 3.1, we shall use the following result (see [23, Proposition 3.1] and [44, Proposition 2.3]).

#### Lemma 3.2

Let $(X, \Vert \cdot \Vert _{X})$ be a separable Banach space and $(\eta _n)$ be a sequence of *X*-valued random variables. Assume that there exists a sequence $\sigma _n$ of real numbers such that:

$$\begin{aligned} \mathbb E\Big ((\eta _n,f)^2\Big )\le \sigma _n^2 \Vert f\Vert _{X'}^2, \quad \forall f\in X'. \end{aligned}$$

Then

$$\begin{aligned} \mathbb E\Big (\sup _n \Vert \eta _n\Vert _X\Big )\le \sup _n \mathbb E(\Vert \eta _n\Vert _X)+ 3 \rho (\sigma _n), \end{aligned}$$

where

$$\begin{aligned} \rho (\sigma _n)= \inf \Big \{\delta \;|\;\sum _n \sigma _n^2\delta ^{-2}\exp (-2^{-1}(\delta \sigma _n^{-1})^2)\le 1 \Big \}. \end{aligned}$$

#### Remark 3.3

As recalled in [44], if $\sigma _n\le \alpha ^n$ then $\rho (\sigma _n)\sim \sqrt{\ln (1-\alpha )^{-1}}$.

We also need the following result, which is an immediate consequence of [12, Lemma 2.5].

#### Lemma 3.4

For any $\alpha \in (0,1)$ and $\delta >0$, we have

3.3 $$\begin{aligned} \Vert Y\Vert _{\mathcal {C}^\alpha _{-\delta }}+\Vert \xi \Vert _{\mathcal {C}^{\alpha -2}_{-\delta }}\le C(\omega ), \quad \hbox { a.s. } \omega . \end{aligned}$$

#### Proof of Prop. 3.1

We split the proof in several steps. First we show the following property:

3.4 $$\begin{aligned} \mathbb E\Big (\Vert \nabla Y \Vert _{B_{r,\infty ,-\delta }^{0}}\Big ) <\infty , \quad \delta \cdot r>2, \quad r\in (2, \infty ), \end{aligned}$$

whose proof is a generalization of [44, Theorem 3.4].

For every fixed $x\in \mathbb R^2$, we have that $\Delta _N \xi (x)$ is a Gaussian random random variable and hence

$$\begin{aligned} \mathbb E\left( \Vert \Delta _N \xi \Vert _{L^r_{-\delta }}^r\right)&=\int _{\mathbb R^2} \mathbb E(\left| \Delta _N \xi (x)\right| ^r) \langle x\rangle ^{-\delta r}dx\\&\le C \int _{\mathbb R^2} \left( \mathbb E\left| \Delta _N\xi (x)\right| ^2\right) ^{r/2} \langle x\rangle ^{-\delta r}dx. \end{aligned}$$

Moreover, we have

$$\begin{aligned} \mathbb E(\left| \Delta _N \xi (x)\right| ^2) =N^4 \mathbb E\Big [\Big (\xi , K(N(x-\cdot )\Big )^2\Big ] = N^{4} \Vert K(N(x-\cdot )\Vert _{L^2}^2= N^2\Vert K\Vert _{L^2}^2 \end{aligned}$$

and hence

3.5 $$\begin{aligned} \mathbb E\left( \Vert \Delta _N \xi \Vert _{L^r_{-\delta }} \right) \le C N \end{aligned}$$

provided that $\delta \cdot r >2$. Also, for $f\in L^{r'}_\delta$, where $\frac{1}{r}+\frac{1}{r'}=1$, we get

3.6 $$\begin{aligned} \begin{aligned} \mathbb E\Big ((\Delta _N \xi ,f)^2\Big )&= \mathbb E\Big ((\xi , \Delta _Nf)^2\Big ) \\&= N^{4} \Vert K(N\cdot )*f\Vert _{L^2}^2 \\&\le N^4 \Vert K(N\cdot )\Vert _{L^\frac{2r}{r+2}}^2 \Vert f\Vert _{L^{r'}}^2 \\&\le C N^{\frac{2(r-2)}{r}}\Vert f\Vert _{L^{r'}_\delta }^2. \end{aligned} \end{aligned}$$

By using the fact that $L^{r'}_{\delta }= (L^r_{-\delta })'$ and (3.5), we can apply Lemma 3.2, where we choose: $X=L^r_{-\delta }$, $\eta _N=N^{-1}\Delta _N \xi$, $\sigma _N=N^{-\frac{1}{r}}$ (see (3.6)). Hence, we obtain

3.7 $$\begin{aligned} \mathbb E(\Vert \xi \Vert _{B_{r,\infty ,-\delta }^{-1}}) =\mathbb E\left( \sup _N N^{-1}\Vert \Delta _N \xi \Vert _{L^r_{-\delta }}\right) \le C + C\rho (N^{-\frac{2}{r}})\le C. \end{aligned}$$

where we have used Remark 3.3 at the last step. The estimate (3.4) clearly follows from (3.7).

Next, we claim that

3.8 $$\begin{aligned} \Vert \rho _\varepsilon *\nabla Y \Vert _{B^0_{r,2,-\delta }} \le C \Vert \rho _\varepsilon \Vert _{B^0_{1,2,\delta }}\Vert \nabla Y \Vert _{B_{r,\infty ,-\delta }^{0}}. \end{aligned}$$

Once this estimate is established, by combining (3.4) with (2.24), we get

$$\begin{aligned} \Vert \nabla Y_\varepsilon \Vert _{L^r_{-\delta }}^2\le C(\omega ) |\ln \varepsilon | \end{aligned}$$

and in turn also

$$\begin{aligned} \Vert \mathbf {:}\nabla Y^2_\varepsilon \mathbf {:}\Vert _{L^r_{-\delta }}^2\le C(\omega ) |\ln \varepsilon | \end{aligned}$$

since $\mathbf {:}\nabla Y^2_\varepsilon \mathbf {:}=\nabla Y_\varepsilon ^2-c_\varepsilon$ with $c_\varepsilon \sim |\ln \varepsilon |$. Hence, (3.1) follows from (3.8), whose proof without weight is in [28, Theorem 2.2]. Here, by using $(\Delta _{\frac{N}{2}} + \Delta _N + \Delta _{2N}) \circ \Delta _N = \Delta _N$, we get

$$\begin{aligned} \Vert \rho _\varepsilon *\nabla Y \Vert _{B^0_{r,2,-\delta }}^2&=\sum _{N-\textrm{dyadic}} \Vert \Delta _N ( \rho _\varepsilon *\nabla Y)\Vert _{L^r_{-\delta }}^2 \\&=\sum _{N-\textrm{dyadic}} \sum _{M = \frac{N}{2}, N, 2N} \Vert \Delta _M \rho _\varepsilon * \Delta _N (\nabla Y)\Vert _{L^r_{-\delta }}^2 \\&\le C\sum _{N-\textrm{dyadic}} \sum _{M = \frac{N}{2}, N, 2N} \Vert \Delta _M \rho _\varepsilon \Vert _{L^1_\delta }^2 \Vert \Delta _N \nabla Y\Vert _{L^r_{-\delta }}^2 \\&\le C \Vert \rho _\varepsilon \Vert _{B^0_{1,2,\delta }}^2 \Vert \nabla Y \Vert _{B_{r,\infty , -\delta }^0}^2, \end{aligned}$$

where we have used the inequality $\langle x\rangle ^{-1}\le \; 2 \langle y\rangle \langle x-y\rangle ^{-1}$. Thus, (3.8) follows.

Then, we consider (3.2). The bound

$$\begin{aligned} \Vert \varphi *\xi _\varepsilon -\varphi *\xi \Vert _{\mathcal {C}^{\beta }_{-\delta }} \le C(\omega ) \varepsilon ^{\kappa }, \quad \hbox { a.s. } \omega \end{aligned}$$

follows by combining (2.33) with (2.23) and (3.3). In order to conclude the proof of (3.2), we notice that again by (2.33) and (3.3) we get

$$\begin{aligned} \Vert Y_\varepsilon -Y\Vert _{\mathcal {C}^{\alpha }_{-\delta }}\le C(\omega ) \varepsilon ^{\kappa }, \quad \hbox { a.s. } \omega , \end{aligned}$$

as desired. $$\square $$

We also establish the following uniform bound and convergence result for $e^{aY_\varepsilon }$ for any $a \in \mathbb R$.

#### Proposition 3.5

For $\alpha \in (0, 1)$, $\delta > 0$, and $a \in \mathbb R$, we have

3.9 $$\begin{aligned} \sup _{\varepsilon \in (0,1)}\Vert e^{a Y_\varepsilon }\Vert _{\mathcal {C}^\alpha _{-\delta }}\le C(\omega ), \quad \hbox { a.s. } \omega . \end{aligned}$$

Moreover, there exists $\kappa \in (0, 1)$ such that

3.10 $$\begin{aligned} \Vert e^{a Y_\varepsilon } - e^{a Y} \Vert _{L^\infty _{- \delta }} \le C(\omega ) \varepsilon ^\kappa , \quad \hbox { a.s. } \omega . \end{aligned}$$

To prove Proposition 3.5, we shall use the following lemma (see [12, Corollary 2.6]).

#### Lemma 3.6

For any $\alpha \in (0, 1)$, $a \in \mathbb R$, and $\delta > 0$, we have

3.11 $$\begin{aligned} \Vert e^{a Y} \Vert _{C^\alpha _{-\delta }} \le C(\omega ), \quad \hbox { a.s. } \omega . \end{aligned}$$

We also need the following result (see [12, Lemma 2.3] and [1, Lemma 5.3]). Below we shall use the functions $\chi _k \in C_c^\infty (\mathbb R^2)$ with $k \in \mathbb N$, $\textrm{supp}\,\chi _k \subseteq [-k - 1, k + 1]^2$, and $\chi _k = 1$ on $[-k, k]^2$.

#### Lemma 3.7

For $\alpha < 1$, there exist $\lambda , \lambda ' > 0$ such that

3.12 $$\begin{aligned} \sup _{k \in \mathbb N} \frac{\mathbb E\Big ( \exp (\lambda \Vert \chi _k \xi \Vert _{\mathcal {C}^{\alpha - 2}}^2) \Big )}{k^{\lambda '}} \le C. \end{aligned}$$

#### Proof of Prop. 3.5

We first establish the uniform bound (3.9), whose proof is similar to that of Corollary 2.6 in [12]. For every $k \in N$, we easily get

3.13 $$\begin{aligned} \begin{aligned} \Vert Y_\varepsilon \Vert _{\mathcal {C}^\alpha ([-k, k]^2)}&= \Vert \rho _\varepsilon *Y \Vert _{\mathcal {C}^\alpha ([-k, k]^2)} \\&\le \Vert Y \Vert _{\mathcal {C}^\alpha ([-k - 1, k + 1]^2)} \\&\le C \Vert G *\chi _{k+2} \xi \Vert _{\mathcal {C}^\alpha } \\&\le C \Vert \chi _{k + 2} \xi \Vert _{\mathcal {C}^{\alpha - 2}} \end{aligned} \end{aligned}$$

uniformly in $\varepsilon \in (0, 1)$, where in the second inequality we used $Y = G *\xi$ and the fact that $\textrm{supp}\,G \subseteq B(0, 1)$, and in the last inequality we used a Schauder estimate (see [37]). We also note that

3.14 $$\begin{aligned} \Vert e^{a Y_{\varepsilon }} \Vert _{\mathcal {C}^\alpha _{-\delta }} \le C \sup _{k \in \mathbb N} \frac{\Vert e^{a Y_\varepsilon } \Vert _{\mathcal {C}^\alpha ([-k, k]^2)}}{k^\delta } \le C \sup _{k \in \mathbb N} \frac{\exp (C|a| \Vert Y_\varepsilon \Vert _{\mathcal {C}^\alpha ([-k, k]^2)})}{k^\delta }. \end{aligned}$$

Thus, for $p > 1$ big enough, by (3.14), (3.13), and (3.12), we get

$$\begin{aligned} \mathbb E\Big ( \sup _{\varepsilon \in (0, 1)} \Vert e^{a Y_\varepsilon } \Vert _{\mathcal {C}^\alpha _{- \delta }}^p \Big )&\le C \mathbb E\bigg ( \bigg | \sup _{\varepsilon \in (0, 1)} \sup _{k \in \mathbb N} \frac{\exp (C|a| \Vert Y_\varepsilon \Vert _{\mathcal {C}^\alpha ([-k, k]^2)})}{k^\delta } \bigg |^p \bigg ) \\&\le C \mathbb E\bigg ( \bigg | \sup _{k \in \mathbb N} \frac{\exp (C|a| \Vert \chi _{k + 2} \xi \Vert _{\mathcal {C}^{\alpha - 2} })}{k^\delta } \bigg |^p \bigg ) \\&\le C \sum _{k = 1}^\infty \frac{\mathbb E\Big ( \exp (p C |a| \Vert \chi _k \xi \Vert _{\mathcal {C}^{\alpha - 2}}) \Big )}{k^{\delta p}} \\&\le C \sum _{k = 1}^\infty \frac{\mathbb E\Big ( \exp (\lambda \Vert \chi _k \xi \Vert _{\mathcal {C}^{\alpha - 2}}^2) \Big )}{k^{2 + \lambda '}} \\&\le C, \end{aligned}$$

where in the last step we used $\exp (p C |a| x) \le C \exp (\lambda x^2)$ and we picked *p* big enough such that $\delta p \ge 2 + \lambda '$. The bound (3.9) then follows.

We now consider (3.10). By (3.2), (3.9), and (3.11), we get

$$\begin{aligned} \Vert e^{a Y_\varepsilon } - e^{a Y} \Vert _{L^\infty _{- \delta }} \le C \Vert Y_\varepsilon - Y \Vert _{L^\infty _{- \frac{\delta }{2}}} \Big ( \Vert e^{a Y_\varepsilon } \Vert _{L^\infty _{- \frac{\delta }{2}}} + \Vert e^{a Y} \Vert _{L^\infty _{- \frac{\delta }{2}}} \Big ) \le C(\omega ) \varepsilon ^\kappa , \end{aligned}$$

which is the desired estimate. $\square$

### Estimates in spaces at negative regularity

The main point of this subsection is the following result where the convergence in negative regularity occurs almost surely in $\omega \in \Omega$.

#### Proposition 3.8

For $\alpha \in (0,1)$, $\delta >0$, and $\varepsilon \in (0, \frac{1}{2})$, there exists $\kappa \in (0,1)$ such that

3.15 $$\begin{aligned} \Vert \nabla Y_\varepsilon - \nabla Y\Vert _{\mathcal {C}^{\alpha -1}_{-\delta }}+ \Vert \mathbf {:}\nabla Y^2_\varepsilon \mathbf {:}-\mathbf {:}\nabla Y^2\mathbf {:}\Vert _{\mathcal {C}^{\alpha -1}_{-\delta }} \le C(\omega ) \varepsilon ^{\kappa }, \quad \hbox { a.s. } \omega . \end{aligned}$$

Moreover, we have

3.16 $$\begin{aligned} \Vert \widetilde{\mathbf {:}\nabla Y^2_\varepsilon \mathbf {:}} - \widetilde{\mathbf {:}\nabla Y^2\mathbf {:}} \Vert _{\mathcal {C}^{\alpha - 1}_{-\delta }} \le C(\omega ) \varepsilon ^{\kappa }, \quad \hbox { a.s. } \omega , \end{aligned}$$

where $\widetilde{\mathbf {:}\nabla Y^2_\varepsilon \mathbf {:}}$ and $\widetilde{\mathbf {:}\nabla Y^2\mathbf {:}}$ are defined in (1.10) and (1.8), respectively.

To prove Proposition 3.8, we need the following result which follows from [20].

#### Lemma 3.9

For any $\alpha \in (0,1)$ and $\delta >0$, we have the bound

3.17 $$\begin{aligned} \Vert \nabla Y\Vert _{\mathcal {C}^{\alpha -1}_{-\delta }}+\Vert \mathbf {:}\nabla Y^2\mathbf {:}\Vert _{\mathcal {C}^{\alpha -1}_{-\delta }}\le C(\omega ), \quad \hbox { a.s. } \omega . \end{aligned}$$

#### Proof of Proposition 3.8

The estimate $\Vert \nabla Y_\varepsilon - \nabla Y\Vert _{\mathcal {C}^{\alpha -1}_{-\delta }} \le C(\omega ) \varepsilon ^{\kappa }$ follows by combining (2.33) with $\Vert \nabla Y\Vert _{\mathcal {C}^{\alpha -1}_{-\delta }}\le C(\omega )$ (see (3.17)). Also, the estimate (3.16) follows immediately from (3.15) and (3.2)

Hence, we focus on the proof of $\Vert \mathbf {:}\nabla Y^2_\varepsilon \mathbf {:}-\mathbf {:}\nabla Y^2\mathbf {:}\Vert _{\mathcal {C}^{\alpha -1}_{-\delta }}\le C(\omega ) \varepsilon ^{\kappa }$. The argument is a little more complicated since Wick products cannot be estimated pathwise. It is shown in [20] that there exists $\kappa _0>0$ such that for all $k\ge 1$ we have:

3.18 $$\begin{aligned} \mathbb E\left( \Vert \mathbf {:}\nabla Y^2_\varepsilon \mathbf {:}-\mathbf {:}\nabla Y^2\mathbf {:}\Vert _{\mathcal {C}^{\alpha -1}_{-\delta }}^k\right) \le C \varepsilon ^{k\kappa _0}. \end{aligned}$$

Recall (1.11) and notice that by elementary considerations we have:

3.19 $$\begin{aligned} \begin{aligned} |c_\varepsilon -c_\eta |&\le \Vert (\rho _\varepsilon -\rho _\eta ) *\nabla G\Vert _{L^2}\Big (\Vert \rho _\varepsilon *\nabla G\Vert _{L^2}+\Vert \rho _\eta *\nabla G\Vert _{L^2}\Big ) \\&\le C|\ln \varepsilon |^{\frac{1}{2}} \Vert \rho _\varepsilon -\rho _\eta \Vert _{L^q}, \quad \forall q\in (2,\infty ), \quad 0<\varepsilon<\eta <\frac{1}{2}, \end{aligned} \end{aligned}$$

where we used $\nabla G\in L^p$ for any $p\in (1,2)$, along with Young convolution inequality and the classical bound $\Vert \rho _\varepsilon *\nabla G\Vert _{L^2}^2=c_\varepsilon \sim |\ln \varepsilon |$ when $\varepsilon \rightarrow 0$. By combining the following inequality (whose proof is elementary)

3.20 $$\begin{aligned} \Vert \rho _\varepsilon -\rho _\eta \Vert _{L^q} \le C \varepsilon ^{-3+2/q} |\varepsilon -\eta |, \quad 0<\varepsilon<\eta <\frac{1}{2}, \; q \ge 1, \end{aligned}$$

with (3.19), we get

3.21 $$\begin{aligned} |c_\varepsilon -c_\eta | \le C \varepsilon ^{-\gamma } |\varepsilon -\eta |, \quad \quad 0<\varepsilon<\eta <\frac{1}{2}, \quad \gamma >1. \end{aligned}$$

Moreover, by (2.45) and the estimate $\Vert \nabla G *f \Vert _{\mathcal {C}^{\gamma }_\chi } \le C \Vert f \Vert _{\mathcal {C}^{\gamma - 1}_\chi }$ ($\gamma , \chi \in \mathbb R$) from [20], we obtain that for $1>\beta >1-\alpha$ and $p> \frac{2}{2-\alpha }$,

3.22 $$\begin{aligned} \begin{aligned} \Vert&\nabla Y_\varepsilon ^2-\nabla Y_\eta ^2\Vert _{\mathcal {C}^{\alpha -1}_{-\delta }} \\&\le C \Vert \nabla Y_\varepsilon - \nabla Y_\eta \Vert _{\mathcal {C}^{\alpha -1}_{-\frac{\delta }{2}}}\left( \Vert \nabla Y_\varepsilon \Vert _{\mathcal {C}^{\beta }_{-\frac{\delta }{2}}}+ \Vert \nabla Y_\eta \Vert _{\mathcal {C}^{\beta }_{-\frac{\delta }{2}}}\right) \\&\le C \Vert \xi _\varepsilon - \xi _\eta \Vert _{\mathcal {C}^{\alpha -2}_{-\frac{\delta }{2}}}\left( \Vert Y_\varepsilon \Vert _{\mathcal {C}^{\beta +1}_{-\frac{\delta }{2}}}+\Vert Y_\eta \Vert _{\mathcal {C}^{\beta +1}_{-\frac{\delta }{2}}}\right) \\&\le C \Vert \xi _\varepsilon - \xi _\eta \Vert _{L^p_{-\frac{\delta }{2}}}\left( \Vert Y_\varepsilon \Vert _{\mathcal {C}^{\beta +1}_{-\frac{\delta }{2}}}+\Vert Y_\eta \Vert _{\mathcal {C}^{\beta +1}_{-\frac{\delta }{2}}}\right) , \end{aligned} \end{aligned}$$

where we used at the last step $L^p_{-\frac{\delta }{2}}\subset \mathcal {C}^{\alpha -2}_{-\frac{\delta }{2}}$ for $p> \frac{2}{2-\alpha }$. In fact, this embedding comes from the following computation (recall (2.3)):

$$\begin{aligned} \sup _N \Big (N^{\alpha -2}\Vert \Delta _N (\langle x \rangle ^{-\frac{\delta }{2}} f) \Vert _{L^\infty }\Big ) \le C \sup _N N^{\alpha -2}\Vert \Delta _N (\langle x \rangle ^{-\frac{\delta }{2}} f) \Vert _{W^{2-\alpha ,p}} \le C \Vert f \Vert _{L^{p}_{-\frac{\delta }{2}}}, \end{aligned}$$

where we used the classical Sobolev embedding $W^{2-\alpha ,p}\subset L^\infty$ for $(2-\alpha )\cdot p>2$.

Now, using the Gaussianity of $\xi$ and Minkowski’s inequality, we have the following estimates for $k\ge p$:

3.23 $$\begin{aligned} \begin{aligned} \mathbb E\left( \Vert \xi _\varepsilon - \xi _\eta \Vert _{L^p_{-\frac{\delta }{2}}}^k \right)&\le \Big (\int _{\mathbb R^2} \langle x\rangle ^{-\frac{p\delta }{2}}\mathbb E\left( |\xi _\varepsilon (x)-\xi _\eta (x)|^k \right) ^\frac{p}{k} dx\Big )^\frac{k}{p} \\&\le C \Big (\int _{\mathbb R^2} \langle x\rangle ^{-\frac{p\delta }{2}}\mathbb E\left( |\xi _\varepsilon (x)-\xi _\eta (x)|^2 \right) ^{\frac{p}{2}}dx\Big )^\frac{k}{p} \\&= C\Vert \rho _\varepsilon -\rho _\eta \Vert _{L^2}^k \\&\le C \varepsilon ^{-2k} |\varepsilon -\eta |^k, \end{aligned} \end{aligned}$$

where $C>0$ depends on *k* and we have used (3.20) at the last step.

Then, by combining (3.22), (3.23), and (2.30), using Cauchy-Schwarz and [12, Lemma 2.7], we have that for any *k* large enough,

3.24 $$\begin{aligned} \mathbb E\left( \Vert \nabla Y_\varepsilon ^2-\nabla Y_\eta ^2\Vert _{\mathcal {C}^{\alpha -1}_{-\delta }}^k\right) \le C \varepsilon ^{-(2+\beta +\zeta )k} |\varepsilon -\eta |^k. \end{aligned}$$

By combining (3.24) with (3.21) and recalling (1.11), we get

3.25 $$\begin{aligned} \mathbb E\left( \Vert \mathbf {:}\nabla Y_\varepsilon ^2\mathbf {:}-\mathbf {:}\nabla Y_\eta ^2\mathbf {:}\Vert _{\mathcal {C}^{\alpha -1}_{-\delta }}^k\right) \le C \varepsilon ^{-(2+\beta +\zeta )k} |\varepsilon -\eta |^k. \end{aligned}$$

On the other hand, by (3.18), we have

3.26 $$\begin{aligned} \mathbb E\left( \Vert \mathbf {:}\nabla Y_\varepsilon ^2\mathbf {:}-\mathbf {:}\nabla Y_\eta ^2\mathbf {:}\Vert _{\mathcal {C}^{\alpha -1}(\langle x\rangle ^{-\delta })}^k\right) \le C \eta ^{k\kappa _0}, \quad 0<\varepsilon<\eta < \frac{1}{2}. \end{aligned}$$

Let us now consider several cases.

**Case 1:** $2\varepsilon <\eta$. We have necessarily $\eta < 2|\varepsilon -\eta |$ and by (3.26),

$$\begin{aligned} \mathbb E\left( \Vert \mathbf {:}\nabla Y_\varepsilon ^2\mathbf {:}-\mathbf {:}\nabla Y_\eta ^2\mathbf {:}\Vert _{\mathcal {C}^{\alpha -1}(\langle x\rangle ^{-\delta })}^k\right) \le C |\varepsilon -\eta |^{k\kappa _0}\le C |\varepsilon -\eta |^{\frac{k\kappa _0}{2+\kappa _0+\beta +\zeta }}. \end{aligned}$$

**Case 2:** $\varepsilon <\eta \le 2\varepsilon$ and $\varepsilon < |\varepsilon -\eta |^{\frac{1}{\kappa _0+2+\beta +\zeta }}$. Then, again by (3.26) we get

$$\begin{aligned} \mathbb E\left( \Vert \mathbf {:}\nabla Y_\varepsilon ^2\mathbf {:}-\mathbf {:}\nabla Y_\eta ^2\mathbf {:}\Vert _{\mathcal {C}^{\alpha -1}(\langle x\rangle ^{-\delta })}^k\right) \le C\varepsilon ^{k\kappa _0}\le C|\varepsilon -\eta |^{\frac{k\kappa _0}{\kappa _0+2+\beta +\zeta }}. \end{aligned}$$

**Case 3:** $\varepsilon <\eta \le 2\varepsilon$ and $\varepsilon \ge |\varepsilon -\eta |^{\frac{1}{\kappa _0+2+\beta +\zeta }}$. In this case, we use (3.25) to get

$$\begin{aligned} \mathbb E\left( \Vert \mathbf {:}\nabla Y_\varepsilon ^2\mathbf {:}-\mathbf {:}\nabla Y_\eta ^2\mathbf {:}\Vert _{\mathcal {C}^{\alpha -1}(\langle x\rangle ^{-\delta })}^k\right) \le C\varepsilon ^{-(2+\beta +\zeta )k} |\varepsilon -\eta |^k \le C |\varepsilon -\eta |^{\frac{k\kappa _0}{2+\kappa _0+\beta +\zeta }}. \end{aligned}$$

Summarizing, we get

$$\begin{aligned} \mathbb E\left( \Vert \mathbf {:}\nabla Y_\varepsilon ^2\mathbf {:}-\mathbf {:}\nabla Y_\eta ^2\mathbf {:}\Vert _{\mathcal {C}^{\alpha -1}_{-\delta }}^k\right) \le C |\varepsilon -\eta |^{\frac{k\kappa _0}{2+\kappa _0+\beta +\zeta }}, \quad \forall \varepsilon ,\, \eta \in \Big ( 0, \frac{1}{2} \Big ). \end{aligned}$$

It remains to choose *k* large enough so that $\frac{k\kappa _0}{2+\kappa _0+\beta +\zeta }>1$ and we may invoke Kolmogorov continuity criterion (see [11, Theorem 3.3]) to deduce that $\varepsilon \mapsto \mathbf {:}\nabla Y_\varepsilon ^2\mathbf {:}$ from [0, 1] to $\mathcal {C}^{\alpha -1}_{-\delta }$ is almost surely Hölder continuous of exponent arbitrarily less than $\frac{\kappa _0}{2+\kappa _0}-\frac{1}{k}$ on [0, 1]. The proof is complete. $\square$

## Linear estimates

We introduce the propagator $S_{A,V}(t)$ associated with

4.1 $$\begin{aligned} \imath \partial _t w=\Delta w- 2\nabla A\cdot \nabla w+V w. \end{aligned}$$

We also denote for shortness

4.2 $$\begin{aligned} H_{A,V}=\Delta - 2\nabla A\cdot \nabla +V. \end{aligned}$$

In the sequel we shall assume that *A* and *V* satisfy:

4.3 $$\begin{aligned}{} & {} \forall \delta>0, ~ \exists C>0 \hbox { s.t. } \Vert \langle x \rangle ^{-\delta } e^{\pm A}\Vert _{L^\infty }\le C; \end{aligned}$$

4.4 $$\begin{aligned}{} & {} \forall \delta>0, ~ \exists C>0 \hbox { s.t. } \Big |\int _{\mathbb R^2} V |\varphi |^2 e^{-2A} dx\Big | \le C \Vert \varphi \Vert _{H^\frac{1}{2}_{\delta }}^2, \quad \forall \varphi \in H^\frac{1}{2}_{\delta }. \end{aligned}$$

Under the assumption (4.3), we have that by (2.3) and classical elliptic regularity,

4.5 $$\begin{aligned} \forall \delta>0, ~ \exists c,C>0 \hbox { s.t. } c\Vert u\Vert _{H^2_{-\delta }}\le \Vert \Delta u e^{-A}\Vert _{L^2}+ \Vert u e^{-A}\Vert _{L^2}\le C \Vert u\Vert _{H^2_\delta }. \end{aligned}$$

Here, we emphasize that the constants *c* and *C* in (4.5) depend only on the constant *C* in (4.3). It is easy to check that any solution to (4.1) satisfies the following conservation laws:

4.6 $$\begin{aligned}{} & {} \frac{\textrm{d}}{\textrm{d}t} \int _{\mathbb R^2} |w(t)|^2 e^{-2A} dx=0; \end{aligned}$$

4.7 $$\begin{aligned}{} & {} \frac{\textrm{d}}{\textrm{d}t} \int _{\mathbb R^2} \Big ( |\nabla w(t)|^2 e^{-2A} - V |w(t)|^2 e^{-2A} \Big ) dx=0. \end{aligned}$$

Next we associate to any couple (*A*, *V*) the following quantity for any given $\delta >0$, $r\in (2,\infty )$:

4.8 $$\begin{aligned} \begin{aligned} |(A,V)|_{\delta , r}&= \Vert \langle x\rangle ^{-\delta } \nabla A e^{-A}\Vert _{L^r} + \Vert \langle x\rangle ^{-2\delta } \nabla A e^{-2A} V\Vert _{L^\frac{r}{2}} \\&\quad +\Vert \langle x\rangle ^{-\delta }V e^{-A}\Vert _{L^r}+\Vert \langle x\rangle ^{-\delta }e^{-(p+2)A}\Vert _{L^\infty } \\&\quad +\Vert \langle x\rangle ^{-\delta } V e^{-(p+2)A}\Vert _{L^r} +\Vert \langle x\rangle ^{-\delta }\nabla A e^{-(p+2)A}\Vert _{L^r} \\&\quad + \Vert \langle x\rangle ^{-\delta } e^{-(p+1)A}\Vert _{L^\infty } + \Vert \langle x\rangle ^{-\delta } \nabla A e^{-(p+1)A}\Vert _{L^r} \\&\quad + \Vert \langle x\rangle ^{-\delta } e^{-pA}\Vert _{L^\infty } + \Vert \langle x\rangle ^{-\delta } \nabla A e^{-pA}\Vert _{L^r}. \end{aligned} \end{aligned}$$

### Linear energy estimates

We first prove some $L^2$ weighted estimates for the propagator $S_{A,V}(t)$ associated with (4.1).

#### Proposition 4.1

Assume *A*, *V* satisfy (4.3) and (4.4).

(i) For every $\delta > 0$, we have

4.9 $$\begin{aligned} \Vert S_{A,V}(t)\varphi \Vert _{L^\infty ((0,\infty );L^2_{-\delta })}\le C \Vert \varphi \Vert _{L^2_{\delta }}. \end{aligned}$$

(ii) For every $T > 0$ and $0< \delta < \delta ^+$ satisfying $\frac{\delta }{2}+2\delta ^+<1$, we have

4.10 $$\begin{aligned} \Vert S_{A,V}(t)\varphi \Vert _{L^\infty ((0,T); L^2_{\delta })}\le C \Vert \varphi \Vert _{H^1_{\delta ^+}}. \end{aligned}$$

(iii) For every $T > 0$, $s \in (0, 1)$, and $0< \delta < \delta ^+$ satisfying $\delta +9\delta ^+<4\,s$, we have

4.11 $$\begin{aligned} \Vert S_{A,V}(t)\varphi \Vert _{L^\infty ((0,T); L^2_{\delta })}\le C \Vert \varphi \Vert _{H^{s^+}_{\frac{\delta ^+}{s}}}. \end{aligned}$$

(iv) For every $T > 0$, $r \in (2, \infty )$, and $0< \delta < \delta ^+$ satisfying $\frac{\delta }{2} + 2\delta ^+<\frac{r-2}{3r+2}$, we have

4.12 $$\begin{aligned} \Vert S_{A,V}(t)\varphi \Vert _{L^\infty ((0, T);H^2_{-\delta })}\le {\mathcal P} \Big (|(A,V)|_{\delta ,r}\Big )\Vert \varphi \Vert _{H^2_{(\frac{3r+2}{r-2})\delta ^+ }}, \end{aligned}$$

where we recall that $\mathcal P (a)$ is a polynomial function depending on *a*.

#### Proof

We denote for simplicity $w(t)=S_{A,V}(t)\varphi$. The conservation of mass (4.6) along with (4.3) imply (4.9). In order to prove (4.10) we rely on (4.6) and (4.7). After integrating (4.6) and (4.7) in time, by recalling (4.4) with $\delta$ replaced by $\frac{\delta }{4}$, we get the following bound:

$$\begin{aligned} \Vert&e^{-A} w(t)\Vert _{L^2}^2+ \Vert e^{-A}\nabla w(t)\Vert _{L^2}^2 \\&\le \Vert e^{-A} w(0)\Vert _{L^2}^2 +\Vert e^{-A}\nabla w(0)\Vert _{L^2}^2 + C \Vert w(0)\Vert _{H^\frac{1}{2}_{\frac{\delta }{4}}}^2 + C \Vert w(t)\Vert _{H^\frac{1}{2}_{\frac{\delta }{4}}}^2 \\&\le C \Vert w(0)\Vert _{H^1_{\frac{\delta }{2}}}^2 + C \Vert w(t)\Vert _{L^2_{\delta }}\Vert w(t)\Vert _{H^1_{-\frac{\delta }{2}}} \\&\le C \Vert w(0)\Vert _{H^1_{\frac{\delta }{2}}}^2 + \frac{C}{\eta } \Vert w(t)\Vert _{L^2_{\delta }}^2 + \eta \Vert w(t)\Vert _{H^1_{-\frac{\delta }{2}}}^2, \end{aligned}$$

where we used interpolation, (4.3), and Cauchy’s inequality with $\eta > 0$ small. By using again (4.3) and taking $\eta > 0$ to be sufficiently small, we get the bound

4.13 $$\begin{aligned} \Vert w(t)\Vert _{H^1_{-\frac{\delta }{2}}}\le C \Vert w(0)\Vert _{H^1_{\delta ^+}} + C\Vert w(t)\Vert _{L^2_{\delta }}.\end{aligned}$$

Next, by fixing $\delta _1 \in (\delta , \delta ^+)$ and following the proof of Lemma 3.1 in [12], we get

4.14 $$\begin{aligned} \begin{aligned} \frac{\textrm{d}}{\textrm{d}t}&\int _{\mathbb R^2} \langle x\rangle ^{2 \delta _1}|w(t)|^2 e^{-2A} \\&=2\text {Re}{\int _{\mathbb R^2} \langle x\rangle ^{2 \delta _1} \, \partial _t w(t) \overline{w}(t)\, e^{-2A}} dx\\&= 2\,\text {Im}{\int _{\mathbb R^2} \langle x\rangle ^{2 \delta _1}(\Delta w(t) -2 \nabla w (t)\cdot \nabla A\, )\bar{w}(t)\,e^{-2A}} dx \\&=-2\,\text {Im}{\int _{\mathbb R^2} \nabla \langle x\rangle ^{2 \delta _1}\cdot \nabla w(t)\,\overline{w} (t) e^{-2A}} dx \\&\le C \,\int _{\mathbb R^2} \langle x\rangle ^{2 \delta _1 - 1} |\nabla w(t)| \,|w(t)| \,e^{-2A} dx. \end{aligned} \end{aligned}$$

After integration in time, by using (4.3) and the Cauchy-Schwarz inequality, we get

4.15 $$\begin{aligned} \begin{aligned} \Vert&w(t) e^{-A}\Vert _{L^2_{ \delta _1 }}^2 \\&\le \Vert w(0) e^{-A}\Vert _{L^2_{ \delta _1 }}^2 +C \int _0^t \Vert \nabla w(\tau )\Vert _{L^2_{2 \delta _1 - 1}} \Vert w(\tau )\Vert _{L^2_{\delta }}d\tau \\&\le \Vert w(0) e^{-A}\Vert _{L^2_{ \delta _1 }}^2 +C\int _0^t \Vert \nabla w(\tau )\Vert _{L^2_{-\frac{\delta }{2}}}^2 d\tau + C \int _0^t \Vert w(\tau )\Vert _{L^2_{\delta }}^2d\tau , \end{aligned} \end{aligned}$$

where we used the condition $\frac{\delta }{2}+2\delta ^+<1$ in order to guarantee $\langle x\rangle ^{2 \delta _1 - 1}\le \langle x\rangle ^{-\frac{\delta }{2}}$. By inserting in (4.15) the estimate (4.13) and by using again (4.3), we deduce

$$\begin{aligned} \Vert w(t)\Vert _{L^2_{\delta }}^2\le C (1+T) \Vert w(0)\Vert _{H^1_{\delta ^+}}^2 + C \int _0^t \Vert w(\tau )\Vert _{L^2_{\delta }}^2 d\tau \end{aligned}$$

and we conclude by the Gronwall inequality.

Next, we focus on (4.11) and we fix $\eta , \mu >0$ such that

4.16 $$\begin{aligned} s \eta -(1-s)\mu =\delta , \quad \eta =\frac{\delta }{s}+\frac{(\delta ^+-\delta )}{2s}. \end{aligned}$$

Then, for every $t\in [0,T]$ we use interpolation to obtain

$$\begin{aligned} \Vert S_{A,V}(t) \varphi \Vert _{L^2_{\delta }}&\le \sum _{{N-\textrm{dyadic}}} \Vert S_{A,V} (t) \Delta _N \varphi \Vert _{L^2_{\delta }}\\&\le C \sum _{{N-\textrm{dyadic}}} \Vert S_{A,V}(t) \Delta _N \varphi \Vert _{L^2_{\eta }}^{s} \Vert S_{A,V}(t) \Delta _N \varphi \Vert _{L^2_{-\mu }}^{1-s} \\&\le C \sum _{{N-\textrm{dyadic}}} \Vert S_{A,V}(t) \Delta _N \varphi \Vert _{L^2_{\eta }}^{s} \Vert \Delta _N \varphi \Vert _{L^2_{\frac{\delta }{s}}}^{1-s}, \end{aligned}$$

where we have used (4.9) at the last step. By (4.10) (that we can use thanks to the conditions $\delta +9\delta ^+<4\,s$ and $\delta ^+>\delta$), we continue the estimate above as follows

$$\begin{aligned} \dots&\le C \sum _{{N-\textrm{dyadic}}} \Vert \Delta _N \varphi \Vert _{H^1_{\frac{\delta ^+}{s}}}^{s} \Vert \Delta _N \varphi \Vert _{L^2_{\frac{\delta }{s}}}^{1-s} \\&\le C \sum _{{N-\textrm{dyadic}}} N^s \Vert \Delta _N \varphi \Vert _{L^2_{\frac{\delta ^+}{s}}}^{s} \Vert \Delta _N \varphi \Vert _{L^2_{\frac{\delta }{s}}}^{1-s} \le C \Vert \varphi \Vert _{H^{s^+}_{\frac{\delta ^+}{s}}}, \end{aligned}$$

where we used (2.8) and (2.12).

For (4.12), we postpone the proof to Sect. 6, since the proof requires a special case of modified energies. Nevertheless, the tools needed to prove (4.12) are elementary (such as Hölder’s inequality) and do not rely on any estimates in Subsect. 4.2 and Sect. 5. $\square$

### Linear Strichartz estimates

In this subsection, we shall need the following norm associated with *A*, *V*:

4.17 $$\begin{aligned} \Vert (A,V)\Vert _{\delta ,k}=\Vert V\Vert _{L^{k}_{-\delta }} + \Vert \nabla A \Vert _{L^{k}_{-\delta }}, \quad k\in [1, \infty ), \quad \delta >0. \end{aligned}$$

We start with some useful lemmas.

#### Lemma 4.2

For every $s\in (0,1)$, there exists $C>0$ such that

4.18 $$\begin{aligned} \Vert (H_{A,V} -\Delta ) u\Vert _{L^2}\le C \Vert (A,V)\Vert _{\delta , \frac{2}{s}} \Vert u\Vert _{H^{1+s}_\delta }, \quad \forall \delta >0. \end{aligned}$$

#### Proof

We have for every $s\in (0,1)$ and $\delta >0$,

$$\begin{aligned} \Vert \nabla u \cdot \nabla A\Vert _{L^2}\le \Vert \nabla A\Vert _{L^{\frac{2}{s}}_{-\delta }} \Vert \nabla u\Vert _{L^{\frac{2}{1-s}}_\delta }\le C \Vert \nabla A\Vert _{L^{\frac{2}{s}}_{-\delta }} \Vert u\Vert _{H^{1+s}_\delta }, \end{aligned}$$

where we have used the embedding $H^s_\delta \subset L^{\frac{2}{1-s}}_\delta$ (see (2.41)) along with (2.17) and (2.3). By a similar argument,

$$\begin{aligned} \Vert V u\Vert _{L^2}\le C \Vert V\Vert _{L^{\frac{2}{s}}_{-\delta }} \Vert u\Vert _{H^{s}_{\delta }}. \end{aligned}$$

The proof is complete. $\square$

#### Lemma 4.3

Let $T>0$ be fixed and *A*, *V* satisfy (4.3) and (4.4). There exists $\bar{\delta }>0$ such that for $\delta \in (0, \bar{\delta })$ and for every $r\in [4, \infty )$, we have^1^

4.19 $$\begin{aligned} \Vert S_{A,V}(t)\varphi \Vert _{L^\infty ((0,T); H^{1+\delta }_{\frac{\delta }{2}(1-3\delta )})}\le \mathcal {P} \Big (|(A,V)|_{\delta ,r}\Big ) \Vert \varphi \Vert _{H_{4\sqrt{\delta }}^{\frac{\sqrt{\delta }(1-\delta )}{\sqrt{2}}+1+\delta ^+}}. \end{aligned}$$

#### Proof

By (4.11) (where we choose $s=\sqrt{\delta }$), there exists $\bar{\delta }>0$ such that for $0<\delta <\bar{\delta }$, we have

4.20 $$\begin{aligned} \Vert S_{A,V}(t)\varphi \Vert _{L^\infty ((0,T); L^2_{2\delta })}\le C \Vert \varphi \Vert _{H^{\sqrt{2\delta }}_{4\sqrt{\delta }}}. \end{aligned}$$

From (4.12), under the extra assumption $r\ge 4$, we get

4.21 $$\begin{aligned} \Vert S_{A,V}(t)\varphi \Vert _{L^\infty ((0, T);H^2_{-\delta })}\le {\mathcal P} \Big (|(A,V)|_{\delta ,r}\Big )\Vert \varphi \Vert _{H^2_{8\delta }}. \end{aligned}$$

Next, we notice that by special case of (2.40) and by (4.20), (4.21) we get:

$$\begin{aligned} \Vert&S_{A,V}(t)\varphi \Vert _{L^\infty ((0,T); H^{1+\delta }_{\frac{\delta }{2}(1-3\delta )})} \\&\le \sum _{{N-\textrm{dyadic}}} \Vert S_{A,V}(t) \Delta _N \varphi \Vert _{L^\infty ((0,T); H^{1+\delta }_{\frac{\delta }{2}(1-3\delta )})}\\ {}&\le \sum _{{N-\textrm{dyadic}}} \Vert S_{A,V}(t)\Delta _N \varphi \Vert _{L^\infty ((0,T); L^2_{2\delta })}^\frac{1-\delta }{2} \Vert S_{A,V}(t)\Delta _N \varphi \Vert _{L^\infty ((0, T);H^2_{-\delta })}^\frac{1+\delta }{2} \\ {}&\le \mathcal {P} \Big (|(A,V)|_{\delta ,r}\Big ) \sum _{{N-\textrm{dyadic}}} \Vert \Delta _N \varphi \Vert _{H^{\sqrt{2\delta }}_{4\sqrt{\delta }}}^\frac{1-\delta }{2} \Vert \Delta _N \varphi \Vert _{H^2_{8\delta }}^\frac{1+\delta }{2} \\ {}&\le \mathcal {P} \Big (|(A,V)|_{\delta ,r}\Big ) \sum _{{N-\textrm{dyadic}}} N^{\frac{\sqrt{\delta }(1-\delta )}{\sqrt{2}}+1+\delta } \Vert \Delta _N \varphi \Vert _{L^2_{4\sqrt{\delta }}}. \end{aligned}$$

The conclusion follows by (2.8). $\square$

#### Lemma 4.4

Let $T>0$ be fixed and *A*, *V* satisfy (4.3) and (4.4). There exists $\bar{\delta }>0$ such that for every $\delta \in (0, \bar{\delta })$ and $r\in [4, \infty )$, we have

4.22 $$\begin{aligned} \Vert S_{A,V}(t) \varphi \Vert _{L^\infty ((0,T);H^{\frac{3}{2}})}\le {\mathcal P}\Big (|(A,V)|_{\delta , r}\Big ) \Vert \varphi \Vert _{H^{\frac{3}{2}+\frac{\sqrt{\delta }^+}{4}}_{\sqrt{\delta }^+}}. \end{aligned}$$

#### Proof

By combining a special case of (2.40), (4.12) and (4.11) (here we assume $\delta >0$ small enough in order to guarantee $\delta +9\delta ^+<4\sqrt{\delta }$, and hence we can apply (4.11) with $s=\sqrt{\delta }$), we get

$$\begin{aligned} \Vert&S_{A,V}(t) \varphi \Vert _{H^{\frac{3}{2}}}\\&\le \sum _{{N-\textrm{dyadic}}} \Vert S_{A,V}(t) \Delta _N \varphi \Vert _{L^\infty ((0,T);H^{\frac{3}{2}})}\\ {}&\le C \sum _{{N-\textrm{dyadic}}} \Vert S_{A,V}(t) \Delta _N \varphi \Vert _{L^\infty ((0,T);H^{2}_{-\frac{\delta }{3}})}^\frac{3}{4} \Vert S_{A,V}(t) \Delta _{N}\varphi \Vert _{L^\infty ((0,T);L^{2}_\delta )}^\frac{1}{4} \\ {}&\le \mathcal {P}\Big (|(A,V)|_{\delta , r}\Big )\sum _{{N-\textrm{dyadic}}} \Vert \Delta _N \varphi \Vert _{H^{2}_{8\delta }}^\frac{3}{4} \Vert \Delta _N \varphi \Vert _{H^{\sqrt{\delta }^+}_{\sqrt{\delta }^+}}^\frac{1}{4}\\ {}&\le \mathcal {P}\Big (|(A,V)|_{\delta , r}\Big )\sum _{{N-\textrm{dyadic}}} N^{\frac{3}{2}+\frac{\sqrt{\delta }^+}{4}} \Vert \Delta _N \varphi \Vert _{L^{2}_{\sqrt{\delta }^+}}. \end{aligned}$$

The conclusion follows by (2.8). $\square$

We are now ready to prove the Strichartz estimates associated with $S_{A,V}(t)$.

#### Proposition 4.5

Let $T>0$ be fixed and *A*, *V* satisfy (4.3) and (4.4). For every $\delta ,s>0$, there exist $\tilde{\delta }, \delta _1, s_1>0$ such that $\frac{\delta _1}{s_1}>1$ and for every $r\in [4,\infty )$,

4.23 $$\begin{aligned} \Vert S_{A,V}(t)\varphi \Vert _{L^l((0,T);L^q)} \le \mathcal {P} \Big (|(A,V)|_{\tilde{\delta },r}\Big ) \mathcal P \Big (\Vert (A,V)\Vert _{\delta _1, \frac{2}{s_1}} \Big ) \Vert \varphi \Vert _{H^{\frac{1}{l}+s}_{\delta }}, \end{aligned}$$

where $$\frac{1}{\,}l+\frac{1}{q}=\frac{1}{2}$$ and $$l>2$$.

#### Proof

Notice that by (4.19), for every $\delta _2, s_2>0$, there exist $\tilde{\delta }, \delta _1, s_1>0$ such that for every $r\in [4,\infty )$,

4.24 $$\begin{aligned} \Vert S_{A,V}(t) \varphi \Vert _{L^\infty ((0,T);H^{1+s_1}_{\delta _1})}\le \mathcal {P}\Big (|(A,V)|_{\tilde{\delta },r}\Big ) \Vert \varphi \Vert _{H^{1+\frac{s_2}{2}}_{\delta _2}}. \end{aligned}$$

Moreover, by (2.5) we can also assume $\frac{\delta _1}{s_1}$ to be arbitrarily large and in particular larger than 1. We also have the following bound for any given $s_2, \delta _2>0$ as a consequence of (4.11):

4.25 $$\begin{aligned} \Vert S_{A,V}(t) \varphi \Vert _{L^\infty ((0,T);L^2)}\le C\Vert \varphi \Vert _{H^{\frac{s_2}{2}}_{\delta _2}}. \end{aligned}$$

Next, following [7, 27] we split the interval [0, *T*] in an essentially disjoint union of intervals of size $$ N^{-1}$$ as

4.26 $$\begin{aligned}{}[0,T]=\bigcup _j I_j \end{aligned}$$

and we aim to estimate $\Vert S_{A,V}(t)\Delta _{N}\varphi \Vert _{L^l(I_j;L^q )}\,.$ Suppose that $I_j=[a,b]$. Then, for $t\in [a,b]$ we can write:

4.27 $$\begin{aligned} \begin{aligned} S_{A,V}(t)\Delta _{N}\varphi&= e^{i(t-a)\Delta } S_{A,V}(a)\Delta _{N}\varphi \\&\quad + i\int _a^t e^{i(t-\tau )\Delta } (H_{A,V} -\Delta ) S_{A,V}(\tau ) \Delta _{N}\varphi d\tau . \end{aligned} \end{aligned}$$

We now estimate each term in the r.h.s. of (4.27). Using the Strichartz estimates on $\mathbb R^2$ (see [9, 17, 26]), and (4.25), we get

$$\begin{aligned} \Vert&e^{i(t-a)\Delta } S_{A,V}(a)\Delta _{N}\varphi \Vert _{L^l(I_j;L^q)}\\&\le C \Vert S_{A,V}(a)\Delta _{N}\varphi \Vert _{L^2} \\&\le C\Vert \Delta _N \varphi \Vert _{H^{\frac{s_2}{2}}_{\delta _2}}\\&\le C N^{-\frac{1}{l}-\frac{s_2}{2}} \sum _{\frac{N}{4} \le M \le 4N} \Vert \Delta _{M}\varphi \Vert _{H^{\frac{1}{l}+s_2}_{\delta _2}} \\ {}&\le C N^{-\frac{1}{l}-\frac{s_2}{2}} \Vert \varphi \Vert _{H^{\frac{1}{l}+s_2}_{\delta _2}} \end{aligned}$$

where we used (2.20) at the second last step. Now we estimate the second term in the r.h.s. of (4.27). Using Minkowski’s inequality, the Strichartz estimates on $\mathbb R^2$, (4.18), and (4.24) (with $s_2$ small), we get

$$\begin{aligned} \Big \Vert&\int _a^t e^{i(t-\tau )\Delta } (H_{A,V} -\Delta ) S_{A,V}(\tau ) \Delta _{N}\varphi d\tau \Big \Vert _{L^l(I_j;L^q)} \\ {}&\le C \int _{I_j} \Vert (H_{A,V} -\Delta ) S_{A,V}(\tau ) \Delta _{N}\varphi \Vert _{L^2} d\tau \\ {}&\le C \Vert (A,V)\Vert _{\delta _1, \frac{2}{s_1}} \mathcal {P} \Big (|(A,V)|_{\tilde{\delta },r}\Big ) N^{-1} \Vert \Delta _{N}\varphi \Vert _{H^{1+\frac{s_2}{2}}_{\delta _2}}\\ {}&\le C \Vert (A,V)\Vert _{\delta _1, \frac{2}{s_1}} \mathcal {P} \Big (|(A,V)|_{\tilde{\delta },r}\Big ) N^{-1} N^{1-\frac{1}{l}-\frac{s_2}{2}} \Vert \varphi \Vert _{H^{\frac{1}{l}+s_2}_{\delta _2}}, \end{aligned}$$

where we used (2.21). Summarizing, we get

$$\begin{aligned} \Vert&S_{A,V}(t)\Delta _{N}\varphi \Vert _{L^l(I_j;L^q)}\\&\le C \mathcal {P} \Big (|(A,V)|_{\tilde{\delta },r}\Big ) \Big (1+\Vert (A,V)\Vert _{\delta _1, \frac{2}{s_1}} \Big ) N^{-\frac{1}{l}-\frac{s_2}{2}} \Vert \varphi \Vert _{H^{\frac{1}{l}+s_2}_{\delta _2}}. \end{aligned}$$

Using that the number of $I_j$ in (4.26) is *O*(*TN*), taking the *l*’th power of the previous bound, and summing over *j*, we get the estimate

$$\begin{aligned} \Vert&S_{A,V}(t)\Delta _{N}\varphi \Vert _{L^l((0,T);L^q)}\\&\le C T^\frac{1}{l} \mathcal {P} \Big (|(A,V)|_{\tilde{\delta },r}\Big ) \Big (1+ \Vert (A,V)\Vert _{\delta _1, \frac{2}{s_1}} \Big ) N^{-\frac{s_2}{2}}\Vert \varphi \Vert _{H^{\frac{1}{l}+s_2}_{\delta _2}} \end{aligned}$$

and hence we conclude by summation over *N*. $\square$

In the sequel, we shall need the following Strichartz estimate.

#### Proposition 4.6

Assume (4.3) and (4.4) and let $T>0$ be fixed. For every $s, \delta >0$ small enough, there exist $s_1, \delta _1, \tilde{\delta }>0$ such that $\frac{\delta _1}{s_1}>1$ and for every $r\in [4,\infty )$, we have

4.28 $$\begin{aligned} \begin{aligned} \Vert&S_{A,V}(t)\varphi \Vert _{L^4((0,T);W^{\frac{3}{4}-s,4})}\\&\le \mathcal {P} \Big (|(A,V)|_{\frac{\delta ^2}{4},r}\Big ) {\mathcal P} \Big (|(A,V)|_{\tilde{\delta },r}\Big ){\mathcal P}\Big (\Vert (A,V)\Vert _{\delta _1, \frac{2}{s_1}}\Big ) \Vert \varphi \Vert _{H^1_{\delta }} \end{aligned} \end{aligned}$$

and

4.29 $$\begin{aligned} \begin{aligned} \Big \Vert&\int _0^t S_{A,V}(t-\tau ) f(\tau ) d\tau \Big \Vert _{L^4((0,T);W^{\frac{3}{4}-s,4})}\\&\le \mathcal {P} \Big (|(A,V)|_{\frac{\delta ^2}{4},r}\Big ) {\mathcal P} \Big (|(A,V)|_{\tilde{\delta },r}\Big ){\mathcal P}\Big (\Vert (A,V)\Vert _{\delta _1, \frac{2}{s_1}}\Big ) \Vert f\Vert _{L^1((0,T);H^1_{\delta })}\, . \end{aligned} \end{aligned}$$

#### Proof

Notice that it is not restrictive to assume $\delta , s$ small enough (we will exploit this fact later). Notice also that (4.29) follows by combining (4.28) with Minkowski’s inequality. Thus, we focus on the proof of (4.28).

For every $s\in (0,\frac{1}{2})$, there exists $q\in (2, \infty )$ such that the following Gagliardo-Nirenberg inequality holds:

$$\begin{aligned} \Vert u\Vert _{W^{\frac{3}{4}-s,4}}\le C \Vert u\Vert _{L^q}^{\frac{1}{2}} \Vert u\Vert _{H^\frac{3}{2}}^{\frac{1}{2}}, \end{aligned}$$

and hence by integration in time and the Hölder inequality in time we get

$$\begin{aligned} \Vert S_{A,V}(t)\varphi \Vert _{L^4((0,T);W^{\frac{3}{4}-s,4})}^4&\le C \Vert S_{A,V}(t)\varphi \Vert _{L^{2}((0,T);L^q)}^{2} \Vert S_{A,V}(t)\varphi \Vert _{L^\infty ((0,T);H^\frac{3}{2})}^{2} \\ {}&\le C \mathcal {P}\Big (|(A,V)|_{\frac{\delta ^2}{4}, r}\Big ) \Vert S_{A,V}(t)\varphi \Vert _{L^{l}((0,T);L^q)}^{2} \Vert \varphi \Vert _{H^{\frac{3}{2}+(\frac{\delta }{8})^+}_{(\frac{\delta }{2})^+}}^2, \end{aligned}$$

where $\frac{1}{l}+\frac{1}{q}=1, l>2$ are Strichartz admissible and we used (4.22) (where $r\in [4,\infty )$ is arbitrary and we have replaced $\delta$ by $\frac{\delta ^2}{4}$). By using the Strichartz estimates (4.23), we can continue the estimate above as follows

$$\begin{aligned} \dots \le \mathcal {P}\Big (|(A,V)|_{\frac{\delta ^2}{4}, r}\Big ) \mathcal {P} \Big (|(A,V)|_{\tilde{\delta },r}\Big ) \mathcal P \Big (|(A,V)\Vert _{\delta _1, \frac{2}{s_1}} \Big ) \Vert \varphi \Vert _{H^{\frac{1}{l}+s}_{\delta }}^2 \Vert \varphi \Vert _{H^{\frac{3}{2}+(\frac{\delta }{8})^+}_{(\frac{\delta }{2})^+}}^2, \end{aligned}$$

where $\tilde{\delta }, \delta _1, s_1>0$ depend on $s, \delta$. Notice that for initial datum $\varphi =\Delta _N \varphi$ which is localized at dyadic frequency *N*, we get from the previous bound that

$$\begin{aligned} \Vert&S_{A,V}(t)\Delta _N \varphi \Vert _{L^4((0,T);W^{\frac{3}{4}-s,4})} \\ {}&\le \mathcal {P} \Big (|(A,V)|_{\frac{\delta ^2}{4},r}\Big ) \mathcal {P} \Big (|(A,V)|_{\tilde{\delta },r}\Big )\mathcal P\Big (\Vert (A,V)\Vert _{\delta _1, \frac{2}{s_1}}\Big ) \Vert \Delta _N \varphi \Vert _{H^{\frac{1}{2l}+\frac{s}{2} +\frac{3}{4}+(\frac{\delta }{16})^+}_{\delta }}. \end{aligned}$$

We conclude (4.28) by summing over *N* and using (2.8), once we notice that $\frac{1}{2l}+\frac{3}{4}<1$ for $l>2$ and $s, \delta >0$ are small enough. $\square$

## Nonlinear estimates

Along this section, we focus on solutions to the following nonlinear problem

5.1 $$\begin{aligned} \imath \partial _t v= H_{A,V} v -\lambda e^{-pA} v|v|^p, \quad \lambda >0 \end{aligned}$$

where $H_{A,V}$ is defined in (4.2).

### Nonlinear energy estimates

We have the following conserved quantities for any solution to (5.1):

5.2 $$\begin{aligned}{} & {} \frac{\textrm{d}}{\textrm{d}t} \int _{\mathbb R^2}| v(t)|^2 e^{-2A} dx=0; \end{aligned}$$

5.3 $$\begin{aligned}{} & {} \begin{aligned}&\frac{\textrm{d}}{\textrm{d}t} \int _{\mathbb R^2} \Big (\frac{1}{2}|\nabla v|^2e^{-2A}-\frac{1}{2}|v(t)|^2 V e^{-2A} \\&+\frac{\lambda }{p+2}|v(t) |^{p+2} e^{-(p+2)A}\Big ) dx=0. \end{aligned} \end{aligned}$$

#### Proposition 5.1

Assume (4.3) and (4.4) and let $T>0$ be fixed. Then, for every $\delta \in (0, \frac{1}{9})$, there exists $C>0$ such that for every solution *v* to (5.1), we have

5.4 $$\begin{aligned}{} & {} \Vert v\Vert _{L^\infty ((0,T);L^2_\delta )}\le C \Big (1+\Vert v(0)\Vert _{H^1_{4\delta }}^{\frac{p+2}{2}}\Big );\end{aligned}$$

5.5 $$\begin{aligned}{} & {} \Vert v\Vert _{L^\infty ((0,T);H^1_{-\delta })}\le C\Big (1+\Vert v(0)\Vert _{H^1_{8\delta }}^{\frac{p+2}{2}}\Big ). \end{aligned}$$

Moreover, for every $q\in [2, \infty )$ and $\delta \in \Big (0, \frac{1}{36(2q-1)}\Big )$, we have

5.6 $$\begin{aligned} \Vert v\Vert _{L^\infty ((0,T);W^{1,q}_{\delta })}\le C \Vert v\Vert _{L^\infty ((0,T);H^2_{-\delta })}^{1-\frac{1}{q}} \mathcal {P}\Big ( \Vert v(0)\Vert _{H^1_{4(2q-1)\delta }}\Big ). \end{aligned}$$

#### Proof

By using conservations (5.2) and (5.3) and recalling that $\lambda >0$, we get

$$\begin{aligned} \int _{\mathbb R^2}&\frac{1}{2}(|\nabla v(t)|^2+|v(t)|^2) e^{-2A} dx-\int _{\mathbb R^2} \frac{1}{2}|v(t)|^2 V e^{-2A}dx \\ {}&\le \int _{\mathbb R^2} \frac{1}{2}(|\nabla v(0)|^2+|v(0)|^2)e^{-2A}dx-\int _{\mathbb R^2} \frac{1}{2}|v(0)|^2 V e^{-2A}dx \\ {}&\quad +\int _{\mathbb R^2} \frac{\lambda }{p+2} |v(0) |^{p+2} e^{-(p+2)A}dx \end{aligned}$$

and by (4.4) we get

5.7 $$\begin{aligned} \begin{aligned} \int _{\mathbb R^2}&\frac{1}{2}(|\nabla v(t)|^2+|v(t)|^2) e^{-2A} dx\\ {}&\le C \Vert v (t)\Vert _{H^\frac{1}{2}_{\chi }}^2 + \int _{\mathbb R^2} \frac{1}{2}(|\nabla v(0)|^2+|v(0)|^2)e^{-2A} dx \\ {}&\quad +\int _{\mathbb R^2} \frac{\lambda }{p+2} |v(0) |^{p+2} e^{-(p+2)A} dx. \end{aligned} \end{aligned}$$

In turn by (4.3) and the Sobolev embedding $H^1_{\delta }\subset L^{p+2}_\delta$, we get

5.8 $$\begin{aligned} \Vert v(t)\Vert _{H^1_{-\delta }}^2\le C\Big (1+\Vert v(0)\Vert _{H^1_{\delta }}^{p+2}\Big )+C \Vert v(t)\Vert _{H^1_{-\delta }}\Vert v(t)\Vert _{L^2_{2\delta }}, \end{aligned}$$

where we used a special case of (2.40) and we chose $\chi = \frac{\delta }{2}$. Hence, by elementary considerations, we get

5.9 $$\begin{aligned} \Vert v(t)\Vert _{H^1_{-\delta }}^2\le C\Big (1+\Vert v(0)\Vert _{H^1_{\delta }}^{p+2}\Big )+ C \Vert v(t)\Vert _{L^2_{2\delta }}^2. \end{aligned}$$

Next, by following the computation in [12, Lemma 3.1] (see also (4.14)), we get

$$\begin{aligned} \frac{\textrm{d}}{\textrm{d}t} \int _{\mathbb R^2} |\langle x\rangle ^{\delta } \,v(t)|^2 e^{-2A} dx \le C \,\int _{\mathbb R^2} \langle x\rangle ^{2\delta -1} |\nabla v(t)| \,|v(t)| \,e^{-2A} dx, \end{aligned}$$

which, by integration in time, (4.3), and the Cauchy-Schwarz inequality, implies that

$$\begin{aligned} \Vert v(t) e^{-A}\Vert _{L^2_\delta }^2 \le \Vert e^{-A}v(0)\Vert _{L^2_\delta }^2 +C \int _0^t \Vert \nabla v(\tau )\Vert _{L^2_{2\delta -1}} \Vert v(\tau )\Vert _{L^2_{\frac{\delta }{2}}}d\tau , \end{aligned}$$

so that by using again (4.3),

$$\begin{aligned} \Vert v(t)\Vert _{L^2_\frac{\delta }{2}}^2 \le C \Vert v(0)\Vert _{L^2_{2\delta }}^2 +C \int _0^t \Vert \nabla v(\tau )\Vert _{L^2_{-\frac{\delta }{4}}}^2 d\tau + C \int _0^t \Vert v(\tau )\Vert _{L^2_{\frac{\delta }{2}}}^2d\tau \end{aligned}$$

where we assumed $\delta \in (0, \frac{1}{9})$ in order to guarantee $\langle x\rangle ^{2\delta - 1}\le \langle x\rangle ^{-\frac{\delta }{4}}$. By combining this estimate with (5.9), where we replace $\delta$ by $\frac{\delta }{4}$, we get

$$\begin{aligned} \Vert v(t)\Vert _{L^2_\frac{\delta }{2}}^2 \le C \Vert v(0)\Vert _{L^2_{2\delta }}^2 +C t \Big (1+\Vert v(0)\Vert _{H^1_{\frac{\delta }{4}}}^{p+2}\Big ) + C \int _0^t \Vert v(\tau )\Vert _{L^2_{\frac{\delta }{2}}}^2d\tau . \end{aligned}$$

We deduce (5.4) by the Gronwall inequality. The estimate (5.5) follows by combining (5.4) and (5.9). Concerning (5.6) we can combine (2.43) with (5.4). $\square$

#### Remark 5.2

We consider (5.1) with $\lambda < 0$. If $0< p < 2$, (5.5) and (5.4) follow from Proposition 3.2 and Corollary 3.3 in [12], respectively. If $p \ge 2$, we further assume that $\Vert v(0) \Vert _{H^1_{\delta _0}} \ll 1$ for some $\delta _0 > 0$. In this case, (5.7) needs to be replaced by

$$\begin{aligned} \int _{\mathbb R^2}&\frac{1}{2}(|\nabla v(t)|^2+|v(t)|^2) e^{-2A} dx\\ {}&\le C \Vert v (t)\Vert _{H^\frac{1}{2}_{\frac{\delta }{2}}}^2 + \int _{\mathbb R^2} \frac{1}{2}(|\nabla v(0)|^2+|v(0)|^2)e^{-2A} dx \\ {}&\quad +\int _{\mathbb R^2} \frac{\lambda }{p+2} |v(0) |^{p+2} e^{-(p+2)A} dx - \int _{\mathbb R^2} \frac{\lambda }{p+2} |v(t) |^{p+2} e^{-(p+2)A} dx. \end{aligned}$$

Thus, instead of (5.8), we obtain

$$\begin{aligned} \Vert v(t)\Vert _{H^1_{-\delta }}^2&\le C \Vert v(0)\Vert _{H^1_{\delta }}^{2} +C \Vert v(t)\Vert _{H^1_{-\delta }}\Vert v(t)\Vert _{L^2_{2p\delta }} + \Vert v(t) \Vert _{L^{p + 2}_{\frac{3p \delta }{p+2}}}^{p + 2} \\&\le C \Vert v(0)\Vert _{H^1_{\delta }}^{2} + \frac{1}{2} \Vert v(t)\Vert _{H^1_{-\delta }}^2 + C \Vert v(t)\Vert _{L^2_{2p\delta }}^2 + \Vert v(t) \Vert _{H^{\frac{p}{p + 2}}_{\frac{3p \delta }{p+2}}}^{p + 2} \\&\le C \Vert v(0)\Vert _{H^1_{\delta }}^{2} + \frac{1}{2} \Vert v(t)\Vert _{H^1_{-\delta }}^2 + C \Vert v(t)\Vert _{L^2_{2p\delta }}^2 + \Vert v(t) \Vert _{H^1_{-\delta }}^p \Vert v(t) \Vert _{L^2_{2p \delta }}^2 \end{aligned}$$

where we take $\delta > 0$ to be sufficiently small and we used (4.3), the Sobolev embedding $H^{\frac{p}{p+2}}_{ \chi } \subset L^{p+2}_{\chi }$ for any $\chi \in \mathbb R$, and (2.40). Assume that $\Vert v(t)\Vert _{H^1_{-\delta }} \le 1$, so that we have the bound

5.10 $$\begin{aligned} \Vert v(t)\Vert _{H^1_{-\delta }}^2 \le C \Vert v(0)\Vert _{H^1_{\delta }}^{2} + C \Vert v(t)\Vert _{L^2_{2p\delta }}^2. \end{aligned}$$

Then, by arguing as in the proof of Proposition 5.1 and using (5.10) instead of (5.9), we get

$$\begin{aligned} \Vert v(t) \Vert _{L^2_{2p \delta }}^2 \le C(1 + t) \Vert v(0) \Vert _{H^1_{4p \delta }}^2 + C \int _0^t \Vert v(\tau ) \Vert _{L^2_{2p \delta }}^2 d\tau . \end{aligned}$$

By applying the Gronwall inequality and taking $\Vert v(0) \Vert _{H^1_{4p \delta }}^2$ to be sufficiently small, we obtain $\Vert v(t) \Vert _{L^2_{2p \delta }}^2 \le \frac{1}{100C}$, so that (5.10) gives $\Vert v(t) \Vert _{H^1_{-\delta }} \le \frac{1}{2}$. Thus, by a standard continuity argument, we get the following two uniform bounds for $\delta > 0$ sufficiently small:

$$\begin{aligned} \Vert v \Vert _{L^\infty ((0, T); L^2_\delta )}&\le 1, \\ \Vert v \Vert _{L^\infty ((0, T); H^1_{-\delta })}&\le 1. \end{aligned}$$

The next proposition will also be useful.

#### Proposition 5.3

Assume (4.3) and (4.4). Let $0<\delta <\min \{\frac{1}{9}, \frac{\bar{\eta }}{9(6-4\bar{\eta })} \}$ and $\bar{\eta }\in (0, 1)$. For every $t>0$ and for every solution *v* to (5.1), we have the bound

5.11 $$\begin{aligned} \Vert v (t)\Vert _{H^{\frac{2}{2-\bar{\eta }}}_\delta } \le C \mathcal {P} \Big (\Vert v(0)\Vert _{H^1_{4\delta (\frac{6-4\bar{\eta }}{\bar{\eta }})}}\Big )\Vert v (t)\Vert _{H^{2}_{-\delta (\frac{1-2\bar{\eta }}{\bar{\eta }})}}^{\bar{\eta }}. \end{aligned}$$

In particular, for every $\eta _0\in (0, \frac{1}{3}), \delta _0>0$, there exists $\bar{\delta }>0$ such that

5.12 $$\begin{aligned} \Vert v (t)\Vert _{H^{\frac{2}{2-\eta _0}}_\delta } \le C \mathcal {P} \Big (\Vert v(0)\Vert _{H^1_{\delta _0}}\Big )\Vert v (t)\Vert _{H^{2}_{-\delta }}^{\eta _0}, \quad \forall \delta \in (0, \bar{\delta }). \end{aligned}$$

#### Proof

By special case of (2.40), we get

$$\begin{aligned} \Vert f\Vert _{H^{1+s}_\delta } \le C \Vert f\Vert _{H^{1-s}_{2\delta }}^{\frac{1-s}{1+s}} \Vert f\Vert _{H^{2}_{-\delta (\frac{1-3s}{2s})}}^{\frac{2s}{1+s}}, \quad \delta >0, \quad s\in (0,1) \end{aligned}$$

and also

$$\begin{aligned} \Vert f\Vert _{H^{1-s}_{2\delta }} \le C \Vert f\Vert _{H^{1}_{-\delta }}^{1-s} \Vert f\Vert _{L^2_{\delta (\frac{3-s}{s})}}^{s}, \quad \delta >0, \quad s\in (0,1). \end{aligned}$$

Summarizing, we obtain

$$\begin{aligned} \Vert f\Vert _{H^{1+s}_\delta } \le C\Vert f\Vert _{H^{1}_{-\delta }}^{\frac{(1-s)^2}{1+s}} \Vert f\Vert _{L^2_{\delta (\frac{3-s}{s})}}^{\frac{s(1-s)}{1+s}} \Vert f\Vert _{H^{2}_{-\delta (\frac{1-3s}{2s})}}^{\frac{2s}{1+s}}, \quad \delta >0, \quad s\in (0,1) \end{aligned}$$

Next, we select $s\in (0,1)$ such that $\bar{\eta }=\frac{2s}{s+1}$, namely $s=\frac{\bar{\eta }}{2-\bar{\eta }}$. We recall that (5.4) and (5.5) are available for solutions *v* to (5.1) and so we get

$$\begin{aligned} \Vert v (t)\Vert _{H^{\frac{2}{2-\bar{\eta }}}_\delta } \le C \mathcal {P} \Big (\Vert v(0)\Vert _{H^1_{8\delta }}\Big ) \mathcal {P} \Big (\Vert v(0)\Vert _{H^1_{4\delta (\frac{6-4\bar{\eta }}{\bar{\eta }})}}\Big )\Vert v (t)\Vert _{H^{2}_{-\delta (\frac{1-2\bar{\eta }}{\bar{\eta }})}}^{\bar{\eta }}. \end{aligned}$$

The conclusion follows since $8\delta \le 4\delta (\frac{6-4\bar{\eta }}{\bar{\eta }})$ for every $\bar{\eta }\in (0,1)$. The bound (5.12) is an easy consequence of (5.11). $\square$

### Nonlinear Strichartz estimates

Along this subsection, *v* denotes any solution to (5.1) and $\delta _0>0$ is a fixed number such that $v(0)\in H^2_{\delta _0}$. We aim at proving local in time Strichartz space-time bounds for the solution *v*. We shall use the quantities introduced respectively in (4.8) and (4.17).

#### Proposition 5.4

Assume (4.3) and (4.4). Let $T>0$ and $\bar{\eta }\in (0,1)$ be fixed. Then, for every $s>0$ and $0<\delta <\min \{\frac{1}{18}, \frac{\bar{\eta }}{18(6-4\bar{\eta })}\}$, there exist $s_1, \delta _1,\tilde{\delta }>0$ such that $\frac{\delta _1}{s_1}>1$ and for every $r\in [4, \infty )$, we have the following bound:

5.13 $$\begin{aligned}{} & {} \begin{aligned} \Vert&v\Vert _{L^4((0,T);W^{{\frac{3}{4}-s},4})} \\&\le \mathcal {P} \Big (|(A,V)|_{\delta ,r}\Big ) \mathcal {P} \Big (|(A,V)|_{\tilde{\delta },r}\Big ) \mathcal {P} \Big (|(A,V)|_{\frac{\delta ^2}{4},r}\Big ) \mathcal P \Big (\Vert (A,V)\Vert _{\delta _1, \frac{2}{s_1}}^2 \Big ) \\&\quad \times \Big [\Vert v(0)\Vert _{H^1_{\delta }} + \mathcal {P} \Big (\Vert v(0)\Vert _{H^1_{8\delta (\frac{6-4\bar{\eta }}{\bar{\eta }})}}\Big )\Vert v\Vert _{L^\infty ((0,T);H^{2}_{-2\delta (\frac{1-2\bar{\eta }}{\bar{\eta }})})}^{(p+1)\bar{\eta }}\Big ]. \end{aligned} \end{aligned}$$

In particular, for every given $\eta _0\in (0,\frac{2}{5})$ and $s>0$, there exists $\bar{\delta }>0$ such that for every $\delta \in (0, \bar{\delta })$, there are $s_1, \delta _1, \tilde{\delta }>0$ with $\frac{\delta _1}{s_1}>1$ and for every $r\in [4,\infty )$,

5.14 $$\begin{aligned}{} & {} \begin{aligned} \Vert&v\Vert _{L^4((0,T);W^{{\frac{3}{4}-s},4})} \\&\le \mathcal {P} \Big (|(A,V)|_{\delta ,r}\Big ) \mathcal {P} \Big (|(A,V)|_{\tilde{\delta },r}\Big ) \mathcal {P} \Big (|(A,V)|_{\frac{\delta ^2}{4},r}\Big ) {\mathcal P}\Big (\Vert (A,V)\Vert _{\delta _1, \frac{2}{s_1}}\Big ) \\&\quad \times \mathcal {P} \Big (\Vert v(0)\Vert _{H^1_{\delta _0}}\Big ) \Vert v\Vert _{L^\infty ((0,T);H^{2}_{-\delta })}^{(p+1)\eta _0}. \end{aligned} \end{aligned}$$

#### Proof

We get by (4.28) and (4.29) that

$$\begin{aligned} \Vert v\Vert _{L^4((0,T);W^{{\frac{3}{4}-s},4})}&\le \mathcal {P} \Big (|(A,V)|_{\tilde{\delta },r}\Big ) \mathcal {P} \Big (|(A,V)|_{\frac{\delta ^2}{4},r}\Big ) \mathcal P \Big (\Vert (A,V)\Vert _{\delta _1, \frac{2}{s_1}}^2 \Big ) \\ {}&\quad \times \Big (\Vert v(0)\Vert _{H^1_{\delta }} + |\lambda |\int _0^T \Vert e^{-pA} v(\tau ) |v(\tau )|^p\Vert _{H^1_{\delta }} d\tau \Big ). \end{aligned}$$

On the other hand, we have

$$\begin{aligned} \Vert&e^{-pA} v |v|^p\Vert _{H^1_{\delta }} \\&\le C \Vert e^{-pA} v |v|^p\Vert _{L^2_{\delta }}+ C \Vert \nabla A e^{-pA} v |v|^p\Vert _{L^2_{\delta }}+ C\Vert e^{-pA} \nabla v |v|^p\Vert _{L^2_{\delta }}\\ {}&\le C \Vert \langle x\rangle ^{-\delta } e^{-pA}\Vert _{L^\infty } \Vert v\Vert _{H^1_{2\delta }} \Vert v\Vert _{L^\infty }^p + C \Vert \langle x\rangle ^{-\delta } \nabla A e^{-pA}\Vert _{L^r} \Vert v\Vert _{L^\frac{2r}{r-2}_{2\delta }} \Vert v\Vert _{L^\infty }^p\\ {}&\le C |(A,V)|_{\delta ,r} \Vert v\Vert _{H^{\frac{2}{2-\bar{\eta }}}_{2\delta }}^{p+1}, \end{aligned}$$

where $v=v(t)$ for some fixed $t\in [0, T]$ and we used the Sobolev embedding $H^{\frac{2}{2-\bar{\eta }}}_{2\delta }\subset L^q_{\delta }$ for every $q\in [2, \infty ]$. By using (5.11), we get

$$\begin{aligned} \Vert e^{-pA} v |v|^p\Vert _{L^\infty ((0,T);H^1_{\delta })}\le C\mathcal {P} \Big (\Vert v(0)\Vert _{H^1_{8\delta (\frac{6-4\bar{\eta }}{\bar{\eta }})}}\Big ) \Vert v\Vert _{L^\infty ((0,T);H^{2}_{-2\delta (\frac{1-2\bar{\eta }}{\bar{\eta }})})}^{(p+1)\bar{\eta }}. \end{aligned}$$

The bound (5.14) easily follows from (5.13). $\square$

As a consequence, we can show the following estimates.

#### Proposition 5.5

Assume (4.3) and (4.4). Let $T>0$, $\eta _0\in (0,\frac{2}{5})$, and $s\in (0,\frac{1}{8})$ be given. Then, there exists $\bar{\delta }>0$ such that for every $\delta \in (0, \bar{\delta })$, there exist $s_1, \delta _1, \tilde{\delta }>0$ with $\frac{\delta _1}{s_1}>1$ and for every $r\in [4,\infty )$,

5.15 $$\begin{aligned} \begin{aligned} \Vert&v\Vert _{L^2((0,T);W^{1,4})}\\&\le \mathcal {P} \Big (|(A,V)|_{\delta ,r}\Big ) \mathcal {P} \Big (|(A,V)|_{\tilde{\delta },r}\Big ) \mathcal {P} \Big (|(A,V)|_{\frac{\delta ^2}{4},r}\Big ) {\mathcal P}\Big (\Vert (A,V)\Vert _{\delta _1, \frac{2}{s_1}}\Big ) \\ {}&\quad \times \mathcal {P} \Big (\Vert v(0)\Vert _{H^1_{\delta _0}}\Big )\Vert v\Vert _{L^\infty ((0,T);H^{2}_{-\delta })}^{\frac{2(1-2s)(p+1)\eta _0}{3}} \Vert v\Vert _{L^\infty ((0,T);H^2_{-\frac{s\delta _0}{4(4-s)}})}^{\frac{(1+4s)(2-s)}{6}}. \end{aligned} \end{aligned}$$

In particular, by choosing $s=\frac{1}{16}$ and $0<\delta <\min \Big \{\bar{\delta }, \frac{\delta _0}{64(4-\frac{1}{16})}\Big \}$, we get

5.16 $$\begin{aligned} \begin{aligned} \Vert&v\Vert _{L^2((0,T);W^{1,4})}\\&\le \mathcal {P} \Big (|(A,V)|_{\delta ,r}\Big ) \mathcal {P} \Big (|(A,V)|_{\tilde{\delta },r}\Big ) \mathcal {P} \Big (|(A,V)|_{\frac{\delta ^2}{4},r}\Big ) {\mathcal P}\Big (\Vert (A,V)\Vert _{\delta _1, \frac{2}{s_1}}\Big )\\&\quad \times \mathcal {P} \Big (\Vert v(0)\Vert _{H^1_{\delta _0}}\Big )\Vert v\Vert _{L^\infty ((0,T);H^{2}_{-\delta })}^{\frac{7}{12}(p+1)\eta _0+\frac{155}{384}} \end{aligned} \end{aligned}$$

#### Proof

We have the following interpolation bound at time fixed:

$$\begin{aligned} \Vert v(t)\Vert _{W^{1,4}}\le C \Vert v(t)\Vert _{W^{\frac{3}{4}-s, 4}}^{\frac{2(1-2s)}{3}} \Vert v(t)\Vert _{H^{2-s}}^{\frac{1+4s}{3}}, \quad s\in \Big (0,\frac{1}{2}\Big ) \end{aligned}$$

and hence by integration in time and using Hölder in time, we get

5.17 $$\begin{aligned} \begin{aligned} \Vert&v(t)\Vert _{L^2((0,T);W^{1,4})}\\&\le C \Vert v(t)\Vert _{L^4((0,T);W^{\frac{3}{4}-s, 4})}^{\frac{2(1-2s)}{3}} \Vert v(t)\Vert _{L^\infty ((0,T);H^{2-s})}^{\frac{1+4s}{3}}, \quad s\in \Big (0,\frac{1}{8}\Big ). \end{aligned} \end{aligned}$$

Next, notice that we have

5.18 $$\begin{aligned} \Vert v(t)\Vert _{H^{2-s}_\frac{s\delta _0}{4(4-s)}} \le C \Vert v(t)\Vert _{L^2_{\frac{\delta _0}{4}}}^\frac{s}{2} \Vert v(t)\Vert _{H^2_{-\frac{s\delta _0}{4(4-s)}}}^\frac{2-s}{2}. \end{aligned}$$

We conclude (5.15) by combining (5.4) with (5.14), (5.17), and (5.18). The estimate (5.16) follows by (5.15) once we notice that the condition $0<\delta <\frac{\delta _0}{64(4-\frac{1}{16})}$ implies that we have the embedding $H^2_{-\delta }\subset H^2_{-\frac{s\delta _0}{4(4-s)}}$ for $s=\frac{1}{16}$. Then one can abosorb the term $\Vert v\Vert _{L^\infty ((0,T);H^2_{-\frac{s\delta _0}{4(4-s)}})}$ in the factor $\Vert v\Vert _{L^\infty ((0,T);H^2_{-\delta })}$ on the r.h.s. in (5.15). $\square$

## Modified energies

Along this section we denote by *v* a solution to the following equation with time-independent *A* and *V*:

6.1 $$\begin{aligned} \imath \partial _t v=\Delta v- 2\nabla A\cdot \nabla v+V v-\lambda e^{-pA}v |v|^{p}, \quad \lambda \ge 0 \end{aligned}$$

and $\delta _0>0$ will denote a fixed given number such that $v(0)\in H^2_{\delta _0}$. The following result has already been used in the linear case ($\lambda =0$).

### Proposition 6.1

Let *v* be solution to (6.1), then we have the following identity:

6.2 $$\begin{aligned} \frac{\textrm{d}}{\textrm{d}t} \mathcal {E}_{A,V}(v(t))= -\lambda \mathcal H_{A,V}(v(t)), \end{aligned}$$

where

$$\begin{aligned} \mathcal {E}_{A,V}(v(t))= \int _{\mathbb R^2} |\Delta v(t)|^2 e^{-2A} dx+\mathcal {F}_{A,V}(v(t)) - \lambda \mathcal {G}_{A,V}(v(t)) \end{aligned}$$

and the energies $\mathcal {F}_{A,V}, \mathcal {G}_{A,V}, \mathcal H_{A,V}$ are defined as follows:

$$\begin{aligned} \mathcal {F}_{A,V}(v(t)) :\!&= -4 \text {Re}\int _{\mathbb R^2} \Delta v(t) \nabla A \cdot \nabla \bar{v}(t) e^{-2A} dx \\ {}&\quad - 4 \int _{\mathbb R^2} (\nabla A\cdot \nabla v)^2 e^{-2A}dx +2 \text {Re}\int _{\mathbb R^2} v(t) V \nabla \bar{v}(t) \cdot \nabla (e^{-2A})dx \\ {}&\quad +2 \text {Re}\int _{\mathbb R^2} \Delta v(t) \bar{v}(t) V e^{-2A}dx + \int _{\mathbb R^2} |v(t)|^2 V^2 e^{-2A}dx, \\ \mathcal {G}_{A,V}(v(t)) :\!&= - \int _{\mathbb R^2} |\nabla v(t)|^2|v(t)|^p e^{-(p+2)A} dx \\&\quad -2 \text {Re}\int _{\mathbb R^2} v(t) \nabla (|v(t)|^p) \cdot \nabla \bar{v}(t) e^{-(p+2)A}dx \\ {}&\quad +\frac{p}{4} \int _{\mathbb R^2} |\nabla (|v(t)|^2)|^2|v(t)|^{p-2} e^{-(p+2)A}dx \\&\quad +\frac{2 }{p+2} \int _{\mathbb R^2} |v(t)|^{p+2} V e^{-(p+2)A}dx\\ {}&\quad +2p\text {Re}\int _{\mathbb R^2} v(t) |v(t)|^p \nabla A \cdot \nabla \bar{v}(t) e^{-(p+2)A}dx, \\ \mathcal H_{A,V}(v(t)):\!&= -\int _{\mathbb R^2} |\nabla v(t)|^2 \partial _t (|v(t)|^p) e^{-(p+2)A} dx\\ {}&\quad -2 \text {Re}\int _{\mathbb R^2} \partial _t v(t) \nabla (|v(t)|^p) \cdot \nabla \bar{v}(t) e^{-(p+2) A} dx\\ {}&\quad -\frac{p}{4} \int _{\mathbb R^2} |\nabla (|v(t)|^2)|^2\partial _t (|v(t)|^{p-2}) e^{-(p+2)A} dx \\&\quad +2p \text {Re}\int _{\mathbb R^2} \partial _t (v(t) |v(t)|^p) \nabla A \cdot \nabla \bar{v}(t) e^{-(p+2)A}dx. \end{aligned}$$

### Proof

In the sequel, we denote by $(\cdot ,\cdot )$ the usual scalar product in $L^2$. We start with the following computation:

6.3 $$\begin{aligned} \begin{aligned} \frac{\textrm{d}}{\textrm{d}t} (\Delta v, \Delta v e^{-2A})&= 2\text {Re}(\partial _t \Delta v, \Delta v e^{-2A}) \\&= 2\text {Re}(\partial _t \Delta v, \imath \partial _t v e^{-2A} ) + 4\text {Re}(\partial _t \Delta v, \nabla A \cdot \nabla ve^{-2A}) \\&\quad -2\text {Re}(\partial _t \Delta v, v Ve^{-2A}) +2\lambda \text {Re}(\partial _t \Delta v, v|v|^p e^{-(p+2)A}) \\ {}&=I+II+III+IV. \end{aligned} \end{aligned}$$

Notice that

6.4 $$\begin{aligned} \begin{aligned} I&=-2\text {Re}(\partial _t \nabla v, \imath \partial _t \nabla v e^{-2A} )-2\text {Re}(\partial _t \nabla v, \imath \partial _t v \nabla (e^{-2A}) )\\&=-2 \text {Im}(\partial _t \nabla v, \partial _t v \nabla (e^{-2A}) ). \end{aligned} \end{aligned}$$

Moreover we have

$$\begin{aligned} II&= 4\text {Re}(\partial _t \Delta v, \nabla A \cdot \nabla ve^{-2A})\\&=2\text {Re}(\Delta v, \partial _t \nabla v \cdot \nabla (e^{-2A})) + 4 \frac{\textrm{d}}{\textrm{d}t}\text {Re}(\Delta v, \nabla A \cdot \nabla ve^{-2A}) \end{aligned}$$

and using again the equation solved by *v*, we obtain

$$\begin{aligned} II&=4 \frac{\textrm{d}}{\textrm{d}t}\text {Re}(\Delta v, \nabla A \cdot \nabla ve^{-2A})-2\text {Im}(\partial _t v, \partial _t \nabla v \cdot \nabla (e^{-2A})) \\ {}&\quad +4\text {Re}(\nabla A \cdot \nabla v, \partial _t \nabla v \cdot \nabla (e^{-2A})) - 2\text {Re}(V v , \partial _t \nabla v \cdot \nabla (e^{-2A})) \\&\quad + 2\lambda \text {Re}(e^{-pA}v|v|^p , \partial _t \nabla v \cdot \nabla (e^{-2A})) \\&=4\frac{\textrm{d}}{\textrm{d}t}\text {Re}(\Delta v, \nabla A \cdot \nabla ve^{-2A})+2 \text {Im}(\partial _t \nabla v, \partial _t v \nabla (e^{-2A})) \\ {}&\quad +4\text {Re}(\nabla A \cdot \nabla v, \partial _t \nabla v \cdot \nabla (e^{-2A}))- 2\text {Re}(V v , \partial _t \nabla v \cdot \nabla (e^{-2A})) \\ {}&\quad + 2\lambda \text {Re}(e^{-pA} v|v|^p , \partial _t \nabla v \cdot \nabla (e^{-2A})). \end{aligned}$$

Hence by (6.4) we get

$$\begin{aligned} II&= 4 \frac{\textrm{d}}{\textrm{d}t}\text {Re}(\Delta v, \nabla A \cdot \nabla ve^{-2A}) -I +4\text {Re}(\nabla A \cdot \nabla v, \partial _t \nabla v \cdot \nabla (e^{-2A})) \\ {}&\quad - 2\text {Re}(V v, \partial _t \nabla v \cdot \nabla (e^{-2A})) +2\lambda \text {Re}(e^{-pA} v|v|^p , \partial _t \nabla v \cdot \nabla (e^{-2A})) \\ {}&= 4\frac{\textrm{d}}{\textrm{d}t}\text {Re}(\Delta v, \nabla A \cdot \nabla ve^{-2A}) -I +4\text {Re}(\nabla A \cdot \nabla v, \partial _t \nabla v \cdot \nabla (e^{-2A})) \\ {}&\quad -2 \frac{\textrm{d}}{\textrm{d}t} \text {Re}(Vv , \nabla v \cdot \nabla (e^{-2A})) +2\text {Re}(V \partial _tv, \nabla v \cdot \nabla (e^{-2A})) \\ {}&\quad +2\lambda \text {Re}(e^{-pA}v|v|^p , \partial _t \nabla v \cdot \nabla (e^{-2A})). \end{aligned}$$

Namely, we have

$$\begin{aligned} I+II&= 2\text {Re}(V \partial _tv, \nabla v \cdot \nabla (e^{-2A})) + 4 \frac{\textrm{d}}{\textrm{d}t}\text {Re}(\Delta v, \nabla A \cdot \nabla ve^{-2A}) \\&\quad +4\text {Re}(\nabla A \cdot \nabla v, \partial _t \nabla v \cdot \nabla (e^{-2A})) - 2\frac{\textrm{d}}{\textrm{d}t} \text {Re}(V v , \nabla v \cdot \nabla (e^{-2A})) \\&\quad +2\lambda \text {Re}(e^{-pA} v|v|^p , \partial _t \nabla v \cdot \nabla (e^{-2A})). \end{aligned}$$

On the other hand, we can compute the last term in the above identity

$$\begin{aligned}&2 \lambda \text {Re}(e^{-pA} v|v|^p , \partial _t \nabla v \cdot \nabla (e^{-2A}))\\ {}&= - 2\lambda \text {Re}(\nabla ( e^{-pA} v|v|^p) , \partial _t \nabla v e^{-2A}) -2\lambda \text {Re}(e^{-pA} v|v|^p , \partial _t \Delta v e^{-2A}) \\ {}&= -2\lambda \text {Re}(\nabla ( e^{-pA}v|v|^p) , \partial _t \nabla v e^{-2A}) -IV \\ {}&= -2\lambda \text {Re}(e^{-pA} \nabla v|v|^p, \partial _t \nabla v e^{-2A}) -2\lambda \text {Re}(e^{-pA}v\nabla (|v|^p), \partial _t \nabla v e^{-2A})\\ {}&\quad -2\lambda \text {Re}(\nabla (e^{-pA}) v |v|^p, \partial _t \nabla v e^{-2A}) -IV\\ {}&=-\lambda (\partial _t (|\nabla v|^2)|v|^p, e^{-(p+2)A}) -2\lambda \frac{\textrm{d}}{\textrm{d}t} \text {Re}(v\nabla (|v|^p), \nabla v e^{-(p+2)A}) \\&\quad +2\lambda \text {Re}(\partial _t v\nabla (|v|^p), \nabla v e^{-(p+2)A}) + 2\lambda \text {Re}(v\nabla \partial _t (|v|^p), \nabla v e^{-(p+2)A}) \\&\quad -2\lambda \text {Re}(\nabla (e^{-pA})v |v|^p, \partial _t \nabla v e^{-2A}) -IV\\ {}&=-\lambda \frac{\textrm{d}}{\textrm{d}t} (|\nabla v|^2|v|^p, e^{-(p+2)A}) +\lambda (|\nabla v|^2 \partial _t (|v|^p), e^{-(p+2)A}) \\&\quad -2\lambda \frac{\textrm{d}}{\textrm{d}t} \text {Re}(v\nabla (|v|^p), \nabla v e^{-(p+2)A}) +2\lambda \text {Re}(\partial _t v\nabla (|v|^p), \nabla v e^{-(p+2)A}) \\&\quad +\frac{\lambda p}{2} (\partial _t (\nabla (|v|^2)|v|^{p-2}), \nabla (|v|^2) e^{-(p+2)A}) \\&\quad -2\lambda \text {Re}(\nabla (e^{-pA})v |v|^p, \partial _t \nabla v e^{-2A}) -IV \\ {}&=-\lambda \frac{\textrm{d}}{\textrm{d}t} (|\nabla v|^2|v|^p, e^{-(p+2)A}) +\lambda (|\nabla v|^2 \partial _t (|v|^p), e^{-(p+2)A}) \\&\quad -2\lambda \frac{\textrm{d}}{\textrm{d}t} \text {Re}(v\nabla (|v|^p), \nabla v e^{-(p+2)A}) +2\lambda \text {Re}(\partial _t v\nabla (|v|^p), \nabla v e^{-(p+2)A}) \\&\quad +\frac{\lambda p}{4} (\partial _t (|\nabla (|v|^2)|^2)|v|^{p-2}, e^{-(p+2)A}) + \frac{\lambda p}{2} (\nabla (|v|^2) \partial _t (|v|^{p-2}), \nabla (|v|^2) e^{-(p+2)A}) \\&\quad -2\lambda \text {Re}(\nabla (e^{-pA})v |v|^p, \partial _t \nabla v e^{-2A}) -IV \\ {}&=-\lambda \frac{\textrm{d}}{\textrm{d}t} (|\nabla v|^2|v|^p, e^{-(p+2)A}) +\lambda (|\nabla v|^2 \partial _t (|v|^p), e^{-(p+2)A}) \\&\quad -2\lambda \frac{\textrm{d}}{\textrm{d}t} \text {Re}(v\nabla (|v|^p), \nabla v e^{-(p+2)A}) +2\lambda \text {Re}(\partial _t v\nabla (|v|^p), \nabla v e^{-(p+2)A}) \\&\quad +\frac{\lambda p}{4} \frac{\textrm{d}}{\textrm{d}t}(|\nabla (|v|^2)|^2|v|^{p-2}, e^{-(p+2)A}) +\frac{\lambda p}{4} (|\nabla (|v|^2)|^2\partial _t (|v|^{p-2}), e^{-(p+2)A}) \\&\quad -2\lambda \text {Re}(\nabla (e^{-pA})v |v|^p, \partial _t \nabla v e^{-2A})-IV\,. \end{aligned}$$

By combining the identities above we get

$$\begin{aligned}I&+II+IV \\&=2\text {Re}(\partial _tvV, \nabla v \cdot \nabla (e^{-2A})) + 2 \frac{\textrm{d}}{\textrm{d}t}\text {Re}(\Delta v, 2 \nabla A \cdot \nabla ve^{-2A}) \\&\quad +4\text {Re}(\nabla A \cdot \nabla v, \partial _t \nabla v \cdot \nabla (e^{-2A})) -2\frac{\textrm{d}}{\textrm{d}t} \text {Re}(Vv, \nabla v \cdot \nabla (e^{-2A}))\\ {}&\quad -\lambda \frac{\textrm{d}}{\textrm{d}t} \text {Re}(|\nabla v|^2|v|^p, e^{-(p+2)A}) +\lambda \text {Re}(|\nabla v|^2 \partial _t (|v|^p), e^{-(p+2)A}) \\&\quad -2\lambda \frac{\textrm{d}}{\textrm{d}t} \text {Re}(v\nabla (|v|^p), \nabla v e^{-(p+2)A}) +2\lambda \text {Re}(\partial _t v\nabla (|v|^p), \nabla v e^{-(p+2)A}) \\&\quad +\frac{\lambda p}{4} \frac{\textrm{d}}{\textrm{d}t}(|\nabla (|v|^2)|^2|v|^{p-2}, e^{-(p+2)A}) +\frac{\lambda p}{4} (|\nabla (|v|^2)|^2\partial _t (|v|^{p-2}), e^{-(p+2)A}) \\&\quad -2\lambda \text {Re}(\nabla (e^{-pA})v |v|^p, \partial _t \nabla v e^{-2A}). \end{aligned}$$

Since $III= -2\frac{\textrm{d}}{\textrm{d}t} \text {Re}(\Delta v, V v e^{-2A})+ 2 \text {Re}(\Delta v, \partial _t v V e^{-2A})$, we obtain the following identity:

$$\begin{aligned} I&+II+III+IV \\&=2\text {Re}(V \partial _tv, \nabla v \cdot \nabla (e^{-2A})) + 4\frac{\textrm{d}}{\textrm{d}t}\text {Re}(\Delta v, \nabla A \cdot \nabla ve^{-2A}) \\&\quad + 4\text {Re}(\nabla A \cdot \nabla v, \partial _t \nabla v \cdot \nabla (e^{-2A})) - 2\frac{\textrm{d}}{\textrm{d}t} \text {Re}(V v , \nabla v \cdot \nabla (e^{-2A})) \\&\quad -\lambda \frac{\textrm{d}}{\textrm{d}t} \text {Re}(|\nabla v|^2|v|^p, e^{-(p+2)A}) +\lambda \text {Re}(|\nabla v|^2 \partial _t (|v|^p), e^{-(p+2)A}) \\&\quad -2\lambda \frac{\textrm{d}}{\textrm{d}t} \text {Re}(v\nabla (|v|^p), \nabla v e^{-(p+2)A}) +2\lambda \text {Re}(\partial _t v\nabla (|v|^p), \nabla v e^{-(p+2)A}) \\&\quad +\frac{\lambda p}{4} \frac{\textrm{d}}{\textrm{d}t}(|\nabla (|v|^2)|^2|v|^{p-2}, e^{-(p+2)A}) +\frac{\lambda p}{4} (|\nabla (|v|^2)|^2\partial _t (|v|^{p-2}), e^{-(p+2)A}) \\&\quad -2\lambda \frac{\textrm{d}}{\textrm{d}t} \text {Re}(\nabla (e^{-pA})v |v|^p, \nabla v e^{-2A}) +2\lambda \text {Re}(\nabla (e^{-pA})\partial _t (v |v|^p), \nabla v e^{-2A})\\&\quad -2\frac{\textrm{d}}{\textrm{d}t}\text {Re}(\Delta v, v V e^{-2A})+ 2 \text {Re}(\Delta v, \partial _t v V e^{-2A}). \end{aligned}$$

Next, by using the equation solved by *v*, we compute the first, the third, and the last term on the r.h.s. above as

$$\begin{aligned} 2&\text {Re}(\partial _tv V, \nabla v \cdot \nabla (e^{-2A})) + 4\text {Re}(\nabla A \cdot \nabla v, \partial _t \nabla v \cdot \nabla (e^{-2A})) \\&\quad +2 \text {Re}(\Delta v, \partial _t v V e^{-2A}) \\ {}&= 2\text {Re}(\partial _tv V, \nabla v \cdot \nabla (e^{-2A}))-8\text {Re}(\nabla A \cdot \nabla v, \partial _t \nabla v \cdot \nabla A e^{-2A}) \\&\quad +4 \text {Re}(\nabla v \cdot \nabla A, \partial _t v Ve^{-2A}) - 2 \text {Re}(V v, \partial _t v V e^{-2A}) \\&\quad + 2 \lambda \text {Re}( e^{-pA}v|v|^p, \partial _t v V e^{-2A}) \\&= -4\text {Re}(\partial _t v V, \nabla v \cdot \nabla A e^{-2A}) - 4 \frac{\textrm{d}}{\textrm{d}t} ((\nabla A\cdot \nabla v)^2, e^{-2A}) \\&\quad + 4 \text {Re}(\nabla v\cdot \nabla A, \partial _t v V e^{-2A}) - 2 \text {Re}(Vv, \partial _t v V e^{-2A}) \\&\quad + 2 \lambda \text {Re}(v|v|^p, \partial _t v V e^{-(p+2)A}) \\ {}&=- 4 \frac{\textrm{d}}{\textrm{d}t} ((\nabla A\cdot \nabla v)^2, e^{-2A})-\frac{\textrm{d}}{\textrm{d}t} (V |v|^2, V e^{-2A}) \\&\quad + \frac{2 \lambda }{p+2} \frac{\textrm{d}}{\textrm{d}t}\text {Re}(|v|^{p+2}, V e^{-(p+2)A}). \end{aligned}$$

The proof of Proposition 6.1 easily follows by combining the above two identities with (6.3). $\square$

Next, we provide some useful estimates on the energies ${\mathcal F}_{A,V}, \mathcal {G}_{A,V}, \mathcal {H}_{A,V}$. We recall that the quantity $|(A,V)|_{\delta ,r}$ has been introduced in (4.8).

### Proposition 6.2

For every $\delta >0$ and $r\in (2, \infty )$, we have

6.5 $$\begin{aligned} |\mathcal {F}_{A,V} (w)|\le \mathcal P\Big (|(A,V)|_{\delta ,r}\Big ) \Big (\Vert e^{-A} \Delta w\Vert _{L^2} \Vert w\Vert _{W_\delta ^{1,\frac{2r}{r-2}}} +\Vert w\Vert _{W_\delta ^{1,\frac{2r}{r-2}}}^2\Big ) \end{aligned}$$

for any generic time-independent function *w*. Moreover, there exists $\bar{\delta }>0$ such that for any given $T>0$ and for every $\delta \in (0, \bar{\delta })$ and $r\in [4, \infty )$, we have

6.6 $$\begin{aligned} \begin{aligned} \sup _{t\in (0,T)}&|\mathcal {F}_{A,V} (v(t))|\le \mathcal P\Big (|(A,V)|_{\delta ,r}\Big ) \mathcal {P}\Big ( \Vert v(0)\Vert _{H^1_{\delta _0}}\Big )\\&\times \Big (\Vert e^{-A} \Delta v\Vert _{L^\infty ((0,T);L^2)}\Vert v\Vert _{L^\infty ((0,T);H^2_{-\delta })}^{\frac{r+2}{2r}} + \Vert v\Vert _{L^\infty ((0,T);H^2_{-\delta })}^{\frac{r+2}{r}}\Big ). \end{aligned} \end{aligned}$$

### Proof

By the Hölder inequality we get:

$$\begin{aligned} |\mathcal {F}_{A,V} (w)|&\le C \Vert e^{-A} \Delta w\Vert _{L^2} \Vert \langle x\rangle ^\delta \nabla w(t)\Vert _{L^{\frac{2r}{r-2}}} \Vert \langle x\rangle ^{-\delta } \nabla A e^{-A}\Vert _{L^r} \\ {}&\quad + C \Vert \langle x\rangle ^{-\delta } e^{-A} \nabla A\Vert _{L^{r}}^2 \Vert \langle x\rangle ^{\delta } \nabla w\Vert _{L^{\frac{2r}{r-2}}}^2 \\ {}&\quad + C \Vert \langle x\rangle ^{-2\delta } \nabla A e^{-2A} V\Vert _{L^\frac{r}{2}} \Vert \langle x\rangle ^\delta \nabla w\Vert _{L^\frac{2r}{r-2}} \Vert \langle x\rangle ^\delta w\Vert _{L^\frac{2r}{r-2}} \\ {}&\quad +C \Vert e^{-A}\Delta w\Vert _{L^2} \Vert \langle x\rangle ^\delta w\Vert _{L^\frac{2r}{r-2}}\Vert \langle x\rangle ^{-\delta }V e^{-A}\Vert _{L^r} \\ {}&\quad +C \Vert \langle x\rangle ^{-\delta }V e^{-A}\Vert _{L^r}^2 \Vert \langle x\rangle ^{\delta } w\Vert _{L^\frac{2r}{r-2}}^2, \end{aligned}$$

which in turn implies (6.5). On the other hand, by (5.6) for $\delta \in (0, \frac{r-2}{36(3r+2)})$, we have

6.7 $$\begin{aligned} \begin{aligned} \Vert v\Vert _{L^\infty ((0,T);W^{1,\frac{2r}{r-2}}_{\delta })}&\le C \Vert v\Vert _{L^\infty ((0,T);H^2_{-\delta })}^{\frac{r+2}{2r}} {\mathcal P}\Big ( \Vert v(0)\Vert _{H^1_{4(\frac{3r+2}{r-2})\delta }}\Big ) \\ {}&\le C \Vert v\Vert _{L^\infty ((0,T);H^2_{-\delta })}^{\frac{r+2}{2r}} \mathcal {P}\Big ( \Vert v(0)\Vert _{H^1_{\delta _0}}\Big ), \end{aligned} \end{aligned}$$

where at the last step we used that for $r>4$ we have $4(\frac{3r+2}{r-2})\delta \le 28 \delta$ and hence $\Vert v(0)\Vert _{H^1_{4(\frac{3r+2}{r-2})\delta }} \le \Vert v(0)\Vert _{H^1_{\delta _0}}$ provided that $\delta >0$ is small enough. The proof is complete. $\square$

We now have the necessary tools to provide the proof of (4.12) in Proposition 4.1.

### Proof of (4.12)

For simplicity, we denote $w (t) = S_{A, V} (t) \varphi$. To prove (4.12), we use (6.2) with $\lambda = 0$ to obtain

$$\begin{aligned} \frac{\textrm{d}}{\textrm{d}t} \int _{\mathbb R^2} \Big (|\Delta w(t)|^2 e^{-2A} + \mathcal F_{A,V}(w(t))\Big ) dx=0, \end{aligned}$$

which in turn after integration in time implies

$$\begin{aligned} \int _{\mathbb R^2} |\Delta w(t)|^2 e^{-2A} dx=\int _{\mathbb R^2} |\Delta w(0)|^2 e^{-2A}dx - \mathcal {F}_{A,V}(w(t))+\mathcal {F}_{A,V}(w(0)). \end{aligned}$$

By (6.5) (whose proof only involves the Hölder inequality) in conjunction with (4.6), we get that for every $\mu >0$,

$$\begin{aligned} \int _{\mathbb R^2}&(|w(t)|^2+ |\Delta w(t)|^2) e^{-2A} dx\\&\le \int _{\mathbb R^2} (|w(0)|^2 + |\Delta w(0)|^2) e^{-2A}dx\\&\quad + \mathcal P\Big ( |(A,V)|_{\delta ,r}\Big ) \Big (\mu \Vert e^{-A} \Delta w(t)\Vert _{L^2}^2+ (1+ \frac{1}{2\mu }) \Vert w(t)\Vert _{W_\delta ^{1,\frac{2r}{r-2}}}^2\Big )\\&\quad +\mathcal P\Big (|(A,V)|_{\delta ,r}\Big ) \Big (\Vert e^{-A} \Delta w(0)\Vert _{L^2} \Vert w(0)\Vert _{W_\delta ^{1,\frac{2r}{r-2}}} +\Vert w(0)\Vert _{W_\delta ^{1,\frac{2r}{r-2}}}^2\Big ). \end{aligned}$$

In particular, we can choose $\mu =\frac{1}{2\mathcal P(|(A,V)|_{\delta ,r})}$ and get

6.8 $$\begin{aligned} \begin{aligned} \frac{1}{2}&\int _{\mathbb R^2} (|w(t)|^2+ |\Delta w(t)|^2) e^{-2A} dx \\&\le \int _{\mathbb R^2} (|w(0)|^2 + |\Delta w(0)|^2) e^{-2A}dx + \mathcal P\Big (|(A,V)|_{\delta ,r}\Big ) \Vert w(t)\Vert _{W_\delta ^{1,\frac{2r}{r-2}}}^2\\&\quad +\mathcal P\Big (|(A,V)|_{\delta ,r}\Big ) \Big (\Vert e^{-A} \Delta w(0)\Vert _{L^2} \Vert w(0)\Vert _{W_\delta ^{1,\frac{2r}{r-2}}} +\Vert w(0)\Vert _{W_\delta ^{1,\frac{2r}{r-2}}}^2\Big ) \end{aligned} \end{aligned}$$

Then, notice that by (2.43), where we choose $q=\frac{2r}{r-2}$, we get

6.9 $$\begin{aligned} \begin{aligned} \Vert w(t)\Vert _{W^{1,\frac{2r}{r-2}}_\delta }&\le C \Vert w(t)\Vert _{H^2_{-\delta }}^\frac{r+2}{2r} \Vert w(t)\Vert _{L^2_{(\frac{3r+2}{r-2})\delta }}^{\frac{r-2}{2r}} \\ {}&\le C \Vert w(t)\Vert _{H^2_{-\delta }}^\frac{r+2}{2r} \Vert w(0)\Vert _{H^1_{(\frac{3r+2}{r-2})\delta ^+ }}^{\frac{r-2}{2r}}, \end{aligned} \end{aligned}$$

where we used at the last step (4.10) (recall our assumptions $\frac{\delta }{2} + 2\delta ^+<\frac{r-2}{3r+2}$ and $\delta ^+>\delta$). We conclude by combining (6.8), (6.9) and (4.5). $\square$

We move on and prove the following estimates for $\mathcal {G}_{A, V}$ and $\mathcal {H}_{A, V}$.

### Proposition 6.3

For any given $T>0$ and $\eta _0\in (0, \frac{1}{3})$, there exists $\bar{\delta }>0$ such that for every $r\in [4,\infty )$ and $\delta \in (0, \bar{\delta })$, we have the bound

6.10 $$\begin{aligned} \begin{aligned}&\sup _{t\in (0,T)}|\mathcal {G}_{A,V} (v(t))|\\&\quad \le |(A,V)|_{\delta ,r} \mathcal {P} \Big (\Vert v(0)\Vert _{H^1_{\delta _0}}\Big ) \Big (1+\Vert v\Vert _{L^\infty ((0,T);H^2_{-\delta })}^{\frac{1}{r}+\frac{1}{2}+\eta _0 (p+2)} \Big ). \end{aligned} \end{aligned}$$

### Proof

We have by the Hölder inequality

6.11 $$\begin{aligned} \begin{aligned} |\mathcal {G}_{A,V} (v(t))|&\le C\Vert \nabla v(t)\Vert _{L^{\frac{2r}{r-2}}}^2 \Vert \langle x\rangle ^\delta |v(t)|^p\Vert _{L^\frac{r}{2}} \Vert \langle x\rangle ^{-\delta }e^{-(p+2)A}\Vert _{L^\infty } \\ {}&\quad +C\Vert \langle x\rangle ^\delta |v(t)|^{p+2}\Vert _{L^\frac{r}{r-1}} \Vert \langle x\rangle ^{-\delta } V e^{-(p+2)A}\Vert _{L^r}\\&\quad +C\Vert \nabla v(t)\Vert _{L^\frac{2r}{r-2}} \Vert \langle x\rangle ^\delta |v(t)|^{p+1}\Vert _{L^2} \Vert \langle x\rangle ^{-\delta }\nabla A e^{-(p+2)A}\Vert _{L^r}. \end{aligned} \end{aligned}$$

Next, notice that by combining the Sobolev embedding $L^q_\rho \subset H^\frac{2}{2-\eta _0}_\rho$ for $q\in [2, \infty ]$ with (5.12), we get

$$\begin{aligned}&\Vert \langle x\rangle ^\delta |v(t)|^p\Vert _{L^\frac{r}{2}} = \Vert v(t)\Vert ^p_{L^\frac{rp}{2}_\frac{\delta }{p}}\le \mathcal {P} \Big (\Vert v(0)\Vert _{H^1_{\delta _0}}\Big )\Vert v(t)\Vert _{H^{2}_{-\delta }}^{\eta _0 p} \\ {}&\Vert \langle x\rangle ^\delta |v(t)|^{p+2}\Vert _{L^\frac{r}{r-1}} = \Vert v(t)\Vert _{L^\frac{r(p+2)}{r-1}_{\frac{\delta }{p+2}}}^{p+2}\le \mathcal {P} \Big (\Vert v(0)\Vert _{H^1_{\delta _0}}\Big )\Vert v(t)\Vert _{H^{2}_{-\delta }}^{\eta _0 (p+2)} \\ {}&\Vert \langle x\rangle ^\delta |v(t)|^{p+1}\Vert _{L^2}= \Vert v(t)\Vert ^{p+1}_{L^{2(p+1)}_{\frac{\delta }{p+1}}} \le \mathcal {P} \Big (\Vert v(0)\Vert _{H^1_{\delta _0}}\Big )\Vert v(t)\Vert _{H^{2}_{-\delta }}^{\eta _0 (p+1)}. \end{aligned}$$

By combining the estimates above with (6.11) and (5.6), where we choose $q=\frac{2r}{r-2}$, we conclude the proof. $\square$

### Proposition 6.4

For any given $T>0$ and $\eta _0\in (0, \frac{1}{3})$, there exists $\bar{\delta }>0$ such that for every $r\in [4,\infty )$ and $\delta \in (0, \bar{\delta })$, we have the bound

6.12 $$\begin{aligned}{} & {} \begin{aligned} \int _0^T |\mathcal {H}_{A,V} (v(\tau ))|d\tau&\le |(A,V)|_{r,\delta } \mathcal {P} \Big (\Vert v(0)\Vert _{H^1_{\delta _0}}\Big ) \Vert \partial _t v e^{-A}\Vert _{L^\infty ((0,T);L^2)} \\&\quad \times \Big (1+ \Vert \nabla v\Vert _{L^2((0,T);L^4)}^2\Big ) \Big (1+ \Vert v\Vert _{L^\infty ((0,T);H^{2}_{-\delta })}\Big )^{\eta _0 p}. \end{aligned}\qquad \end{aligned}$$

### Proof

We only focus on the case when $p > 1$. The case $p = 1$ will follow in a similar (and easier) manner. Notice that by the Hölder inequality, one can estimate

$$\begin{aligned} |\mathcal {H}_{A,V}&(v(t))| \le C \Vert \nabla v(t)\Vert _{L^4}^2 \Vert \partial _t v e^{-A}\Vert _{L^2} \Vert \langle x\rangle ^\delta |v(t)|^{p-1}\Vert _{L^\infty } \Vert \langle x\rangle ^{-\delta } e^{-(p+1)A}\Vert _{L^\infty } \\ {}&\quad +C \Vert \nabla v(t)\Vert _{L^4} \Vert \partial _t v e^{-A}\Vert _{L^2} \Vert \langle x\rangle ^\delta |v(t)|^{p}\Vert _{L^\frac{4r}{r-4}} \Vert \langle x\rangle ^{-\delta } |\nabla A|e^{-(p+1)A}\Vert _{L^r}. \end{aligned}$$

By combining (5.12) with the Sobolev embedding $H^\frac{2}{2-\eta _0}_\rho \subset L^q_\rho$ for $q\in [2, \infty ]$, we get

$$\begin{aligned} \Vert \langle x\rangle ^\delta |v(t)|^{p}\Vert _{L^\frac{4r}{r-4}}= \Vert v(t)\Vert _{L^\frac{4rp}{r-4}_\frac{\delta }{p}}^p\le \mathcal {P} \Big (\Vert v(0)\Vert _{H^1_{\delta _0}}\Big )\Vert v(t)\Vert _{H^{2}_{-\delta }}^{\eta _0 p} \end{aligned}$$

and

$$\begin{aligned} \Vert \langle x\rangle ^\delta |v(t)|^{p-1}\Vert _{L^\infty }= \Vert v(t)\Vert _{L^\infty _{\frac{\delta }{p-1}}}^{p-1}\le \mathcal {P} \Big (\Vert v(0)\Vert _{H^1_{\delta _0}}\Big )\Vert v(t)\Vert _{H^{2}_{-\delta }}^{\eta _0 (p-1)}. \end{aligned}$$

After integration in time and the Hölder inequality w.r.t. time variable, we obtain (6.12). $\square$

## $H^2$ a-priori bound

We introduce the following family of regularized and localized potentials, for $\varepsilon >0$ and $n\in \mathbb N$:

$$\begin{aligned} A_{\varepsilon , n}=\theta _n Y_\varepsilon , \quad V_{\varepsilon , n}= \theta _n \widetilde{\mathbf {:}\nabla Y^2_\varepsilon \mathbf {:}}\end{aligned}$$

where $\theta _n (x) = \theta (\frac{x}{n})$, $\theta \in C^\infty _0(\mathbb R^2)$, $\theta \ge 0$, and $\theta (0)=1$. We first notice that due to (3.9), if we choose $A=A_{\varepsilon , n}$ and $V=V_{\varepsilon , n}$, then the condition (4.3) holds uniformly w.r.t. *n* and $\varepsilon$ with the constant *C* replaced by a random constant $C(\omega )$. The same for (4.4) which is satisfied uniformly w.r.t. $n, \varepsilon$ with a random constant $C(\omega )$. In order to prove this fact combine (3.15), (3.17), (3.2), (3.3), and [12] (more precisely, see at page 1160 three lines below (33)).

We introduce, following the notation (4.2), the operators

$$\begin{aligned} H_{\varepsilon , n}= H_{A_{\varepsilon , n},V_{\varepsilon , n}} \end{aligned}$$

as well as the associated nonlinear Cauchy problem

7.1 $$\begin{aligned} \imath \partial _t v_{\varepsilon , n}=H_{\varepsilon , n} v_{\varepsilon , n}+\lambda e^{-pA_{\varepsilon , n}}v_{\varepsilon ,n} |v_{\varepsilon ,n}|^{p}, \quad v_{\varepsilon , n}(0)=v_0, \quad \lambda \ge 0 \end{aligned}$$

where $v_0=u_0e^{Y}\in H^2_{\delta _0}$ for some fixed $\delta _0 > 0$. The main point in this section is to establish the following a-propri bound for any sufficiently small $\delta > 0$:

7.2 $$\begin{aligned} \sup _{n\in \mathbb N} \Vert v_{\varepsilon , n}\Vert _{L^\infty ((0,T);H^2_{-\delta })}\le C(\omega ) |\ln \varepsilon |^{C} \mathcal {P} \Big ( \Vert v_0 \Vert _{H_{\delta _0}^2} \Big ), \quad \hbox { a.s. } \omega \end{aligned}$$

for any given $T>0$.

We now focus on proving the bound (7.2). Recall the quantity $|(A, V)|_{\delta , r}$ defined in (4.8). Assume that $\delta >0$ has been fixed in such a way that (6.6), (6.10) and (6.12) are satisfied with the choice $A =A_{\varepsilon , n}, V=V_{\varepsilon , n}$. On the other hand, one can show that once $\delta$ is fixed, one can choose $r \ge 4$ large enough in such a way that

7.3 $$\begin{aligned} \sup _{n\in \mathbb N} |(A_{\varepsilon , n},V_{\varepsilon , n})|_{\delta ,r}\le C(\omega ) O(|\ln \varepsilon |^{C}), \quad \hbox { a.s. } \omega . \end{aligned}$$

This bound follows from (4.8), (3.1), and (3.9) (the cut-off $\theta \big ( \frac{x}{n} \big )$ can be easily handled by an elementary argument and the bounds are uniform in $n \in \mathbb N$). Moreover, by (6.2) we get

7.4 $$\begin{aligned} \begin{aligned} \int _{\mathbb R^2}&|\Delta v_{\varepsilon , n}(t)|^2 e^{-2A_{\varepsilon , n}} dx \\&=\int _{\mathbb R^2} |\Delta v_{\varepsilon , n}(0)|^2 e^{-2A_{\varepsilon , n}}dx - \mathcal {F}_{_{\varepsilon , n}}(v_{\varepsilon , n}(t)) + \mathcal {F}_{_{\varepsilon , n}}(v_{\varepsilon , n}(0)) \\&\quad - \lambda \mathcal {G}_{\varepsilon ,n}(v_{\varepsilon , n}(t))+ \lambda \mathcal {G}_{\varepsilon ,n}(v_{\varepsilon , n}(0))+\int _0^t \mathcal {H}_{\varepsilon ,n}(v_{\varepsilon , n}(\tau ))d\tau \end{aligned} \end{aligned}$$

where

$$\begin{aligned} \mathcal {F}_{\varepsilon ,n}=\mathcal {F}_{A_{\varepsilon , n},V_{\varepsilon , n}}, \quad \mathcal {G}_{\varepsilon ,n}={\mathcal G}_{A_{\varepsilon , n},V_{\varepsilon , n}}, \quad {\mathcal H}_{\varepsilon ,n}=\mathcal {H}_{A_{\varepsilon , n},V_{\varepsilon , n}} \end{aligned}$$

are the energies defined along Proposition 6.1 with $A =A_{\varepsilon , n}$ and $V=V_{\varepsilon , n}$. Next, we apply (6.6), (6.10) and (6.12) with $A =A_{\varepsilon , n}$ and $V=V_{\varepsilon , n}$. By using (6.6) in conjunction with (7.3) and by choosing *r* large enough, we deduce

7.5 $$\begin{aligned} \begin{aligned}&\sup _{t\in [0,T]} |\mathcal {F}_{\varepsilon , n}(v_{\varepsilon , n}(t))| \le C(\omega )|\ln \varepsilon |^C \mathcal {P}\Big ( \Vert v_{\varepsilon , n}(0)\Vert _{H^1_{\delta _0}}\Big ) \\&\times \Big (\Vert e^{-A_{\varepsilon , n}} \Delta v_{\varepsilon , n}\Vert _{L^\infty ((0,T);L^2)}\Vert v_{\varepsilon , n}\Vert _{L^\infty ((0,T);H^2_{-\delta })}^{1^-} + \Vert v_{\varepsilon , n}\Vert _{L^\infty ((0,T);H^2_{-\delta })}^{2^-}\Big ) \end{aligned} \end{aligned}$$

for suitable $1^-\in (0,1)$ and $2^-\in (1,2)$. Moreover, we can choose $\eta _0$ in such a way that

7.6 $$\begin{aligned}{} & {} \frac{1}{r}+\frac{1}{2}+\eta _0 (p+2)\in (0,1); \end{aligned}$$

7.7 $$\begin{aligned}{} & {} \frac{7}{6}(p+1)\eta _0+\frac{155}{192}+\eta _0 p\in (0,1); \end{aligned}$$

7.8 $$\begin{aligned}{} & {} \eta _0(p+1)\in (0,1). \end{aligned}$$

Hence by (6.10), (7.3), and (7.6), we obtain

7.9 $$\begin{aligned} \begin{aligned} \sup _{t\in [0,T]}&|\mathcal {G}_{\varepsilon , n}(v_{\varepsilon , n}(t))|\\&\le C(\omega ) |\ln \varepsilon |^C \mathcal {P} \Big (\Vert v_{\varepsilon , n}(0)\Vert _{H^1_{\delta _0}}\Big ) \Big ( 1+\Vert v_{\varepsilon , n}(t)\Vert _{L^\infty ((0,T);H^{2}_{-\delta })}^{1^-} \Big ), \end{aligned} \end{aligned}$$

where $1^-\in (0,1)$. Notice also that we have (by choosing *r* even larger than above)

$$\begin{aligned} \sup _n |(A_{\varepsilon , n},V_{\varepsilon , n})|_{\frac{\delta ^2}{4},r}\le C(\omega )|\ln \varepsilon |^C, \quad \sup _n |(A_{\varepsilon , n},V_{\varepsilon , n})|_{\tilde{\delta },r}\le C(\omega )|\ln \varepsilon |^C \hbox { a.s. } \omega , \end{aligned}$$

where $\tilde{\delta }>0$ is the one that appears in (5.16). Moreover, by (3.1) in conjunction with the fact that in (5.16) we can assume $\frac{\delta _1}{s_1}>1$, we also have (see (4.17))

7.10 $$\begin{aligned} \sup _n \Vert (A_{\varepsilon , n},V_{\varepsilon , n})\Vert _{\delta _1, \frac{2}{s_1}}\le C(\omega )O(|\ln \varepsilon |^C) \hbox { a.s. } \omega \end{aligned}$$

and hence by combining (6.12) with (5.16) and (7.7), we get

7.11 $$\begin{aligned} \begin{aligned} \int _0^T |\mathcal {H}_{\varepsilon , n}&(v_{\varepsilon , n}(\tau ))|d\tau \le C(\omega )|\ln \varepsilon |^C \mathcal {P}\Big ( \Vert v_{\varepsilon , n}(0)\Vert _{H^1_{\delta _0}}\Big ) \\&\times \Vert \partial _t v_{\varepsilon , n} e^{-A_{\varepsilon , n}}\Vert _{L^\infty ((0,T);L^2)} \Big (1+ \Vert v_{\varepsilon , n}\Vert _{L^\infty ((0,T);H^{2}_{-\delta })}\Big )^{1^-}, \end{aligned} \end{aligned}$$

where $1^-\in (0,1)$. Next we notice that by using the equation solved by $v_{\varepsilon , n}$, we get by the Hölder inequality and the Sobolev embedding $H^\frac{2}{2-\eta _0}_\delta \subset L^q_\delta$, $q\in [2, \infty ]$, in conjunction with (5.6), (5.12), (7.10), and (7.8),

7.12 $$\begin{aligned} \begin{aligned} \Vert&\partial _t v_{\varepsilon , n}(t) e^{-A_{\varepsilon , n}}\Vert _{L^2}\\&\le \Vert \Delta v_{\varepsilon , n}(t) e^{-A_{\varepsilon , n}}\Vert _{L^2}\\&\quad + C(\omega )|\ln \varepsilon |^C \mathcal {P} \Big (\Vert v_{\varepsilon , n}(0)\Vert _{H^1_{\delta _0}}\Big )\Vert v_{\varepsilon , n}\Vert _{L^\infty ((0,T);H^2_{-\delta })}^{1^-}\\&\quad +C(\omega ) |\ln \varepsilon |^C \Vert v_{\varepsilon , n}(t)|v_{\varepsilon , n}(t)|^p\Vert _{L^2_\delta } \\&\le \Vert \Delta v_{\varepsilon , n}(t) e^{-A_{\varepsilon , n}}\Vert _{L^2}\\&\quad + C(\omega ) |\ln \varepsilon |^C \mathcal {P} \Big (\Vert v_{\varepsilon , n}(0)\Vert _{H^1_{\delta _0}}\Big ) \Vert v_{\varepsilon , n}\Vert _{L^\infty ((0,T);H^2_{-\delta })}^{1^-} \\ {}&\quad + C(\omega ) |\ln \varepsilon |^C \mathcal {P} \Big (\Vert v_{\varepsilon , n}(0)\Vert _{H^1_{\delta _0}}\Big )\Vert v_{\varepsilon , n}\Vert _{L^\infty ((0,T);H^2_{-\delta })}^{\eta _0(p+1)} \\ {}&\le \Vert \Delta v_{\varepsilon , n}(t) e^{-A_{\varepsilon , n}}\Vert _{L^2}\\ {}&\quad + C(\omega ) |\ln \varepsilon |^C \mathcal {P} \Big (\Vert v_{\varepsilon , n}(0)\Vert _{H^1_{\delta _0}}\Big )\Vert v_{\varepsilon , n}\Vert _{L^\infty ((0,T);H^2_{-\delta })}^{1^-} \end{aligned} \end{aligned}$$

for a suitable $1^{-}\in (0,1)$. Hence, we can gather together (7.4), (7.5), (7.9), (7.11), and (7.12) and we get by elementary manipulations that

$$\begin{aligned} \int _{\mathbb R^2}&|\Delta v_{\varepsilon , n}(t)|^2 e^{-2A_{\varepsilon , n}} dx\le C\int _{\mathbb R^2} |\Delta v_{\varepsilon , n}(0)|^2 e^{-2A_{\varepsilon , n}}dx \\ {}&+ C \Vert v_{\varepsilon , n}\Vert _{L^\infty ((0,T);H^2_{-\delta })}^{2^-}+C(\omega )|\ln \varepsilon |^C+ {\mathcal P} \Big (\Vert v_{\varepsilon , n}(0)\Vert _{H^1_{\delta _0}}\Big ), \end{aligned}$$

which in turn by (4.5) implies

$$\begin{aligned} \Vert v_{\varepsilon , n}&\Vert _{L^\infty ((0,T);H^2_{-\delta })}^2\le \mathcal {P}\Big ( \Vert v_{\varepsilon , n}(0)\Vert _{H^2_{\delta _0}}\Big ) +C \Vert v_{\varepsilon , n}\Vert _{L^\infty ((0,T); H^2_{-\delta })}^{2^-}+ C(\omega )|\ln \varepsilon |^C. \end{aligned}$$

By an elementary continuity argument we get (7.2).

## Proof of the main result

In this section, we first prove Theorem 1.1, global well-posedness of the mollified equation (1.9). After that, we prove Theorem 1.2, the convergence of the solutions of the mollified problem to a unique solution of (1.7).

### Proof of Theorem 1.1

We first follow the steps from [12, Proposition 2.11] to show the existence of a solution $v_\varepsilon$ to (1.9), where $\varepsilon \in (0, \frac{1}{2})$ is fixed. Fix $\delta _0 > 0$, $T > 0$, and let $v_0 \in H_{\delta _0}^2$. By (7.2), (5.12), and (2.40), we have the following bound for any $\gamma \in (1, 2)$ and for some $\delta > 0$:

8.1 $$\begin{aligned} \sup _{n \in \mathbb N} \Vert v_{\varepsilon , n} \Vert _{\mathcal {C}([0, T); H^\gamma _\delta )} \le C(\omega ) |\ln \varepsilon |^C \mathcal {P} \Big ( \Vert v_0 \Vert _{H^2_{\delta _0}} \Big ), \quad \hbox { a.s. } \omega \end{aligned}$$

where the bound is uniform in $n \in \mathbb N$. Also, by (3.2), (3.15), and (3.9), for any $\alpha \in (0, 1)$, $0< \delta ^- < \delta$, and $\varepsilon \in (0, \frac{1}{2})$ we have the following bounds:

8.2 $$\begin{aligned} \sup _{n \in \mathbb N} \Big ( \Vert \theta _n Y_\varepsilon \Vert _{\mathcal {C}^\alpha _{-\delta ^-}} + \Vert \theta _n \widetilde{\mathbf {:}\nabla Y^2_{\varepsilon }\mathbf {:}} \Vert _{\mathcal {C}^{\alpha - 1}_{- \delta ^-}} + \Vert e^{-p \theta _n Y_\varepsilon } \Vert _{L^\infty _{-\delta ^-}} \Big ) \le C(\omega ), \quad \hbox { a.s. } \omega . \end{aligned}$$

Using the equation (1.12), (8.1), (8.2), and (2.41), we can easily deduce that $\{ \partial _t v_{\varepsilon , n} \}_{n \in \mathbb N}$ is bounded (uniformly in $n \in \mathbb N$) in $\mathcal {C} ([0, T); H^{\gamma -2}_{\delta '})$ for any $0< \delta ' < \delta$. By the Arzelà-Ascoli theorem along with the compact embedding (2.5), we obtain a convergent subsequence $\{v_{\varepsilon , n_k}\}_{k \in \mathbb N}$ in $\mathcal {C} ([0, T); H^{\gamma _1 - 2}_{\delta ''})$ for any $\gamma _1 < \gamma$ and $\delta '' < \delta$, and we denote the limit as $v_\varepsilon$. By (7.2) and (2.40), the convergence also holds in $\mathcal {C} ([0, T); H^s_{\delta _1})$ for any $s \in (1, 2)$ and some $\delta _1 > 0$. Also, by (7.2), the Banach-Alaoglu theorem, and taking a further subsequence if necessary, we obtain the following bound:

8.3 $$\begin{aligned} \Vert v_\varepsilon \Vert _{L^\infty ((0, T); H^2_{- \tilde{\delta }})} \le C(\omega ) |\ln \varepsilon |^C \mathcal {P} \Big ( \Vert v_0 \Vert _{H^2_{\delta _0}} \Big ),\quad \hbox { a.s. } \omega \end{aligned}$$

for some $\tilde{\delta }> 0$. Furthermore, $v_\varepsilon$ satisfies the equation (1.9).

Next, we show the uniqueness of $v_\varepsilon$ in $\mathcal {C} ([0, T); H^s_{\delta _1})$. Assume that $v_{\varepsilon }$ and $w_{\varepsilon }$ are two solutions to (1.9). Define

$$\begin{aligned} r_\varepsilon (t) = v_\varepsilon (t) - w_\varepsilon (t), \quad t \in [0,T). \end{aligned}$$

Then, $r_\varepsilon$ satisfies the equation:

$$\begin{aligned} \imath \partial _t r_\varepsilon = \Delta r_\varepsilon + r_\varepsilon \widetilde{\mathbf {:}\nabla Y^2_{\varepsilon }\mathbf {:}} - 2 \nabla r_\varepsilon \nabla Y_\varepsilon - \lambda e^{-p Y_\varepsilon } (|v_\varepsilon |^p v_\varepsilon - |w_\varepsilon |^p w_\varepsilon ), \quad r_\varepsilon (0) = 0. \end{aligned}$$

Using the equation for $r_\varepsilon$, we can deduce that

$$\begin{aligned} \frac{1}{2} \frac{\textrm{d}}{\textrm{d}t} \int _{\mathbb R^2} |r_\varepsilon (t)|^2 e^{-2 Y_\varepsilon } dx = \lambda \text {Im}\int _{\mathbb R^2} \bar{r}_\varepsilon (t) \big ( |v_\varepsilon (t)|^p v_\varepsilon (t) - |w_\varepsilon (t)|^p w_\varepsilon (t) \big ) e^{-(p + 2) Y_\varepsilon } dx. \end{aligned}$$

Thus, using the embedding $H^s_{\delta _1} \subset L_{\delta _1}^\infty$ and (3.9), there exists $\delta > 0$ small enough such that

$$\begin{aligned} \frac{1}{2} \frac{\textrm{d}}{\textrm{d}t} \int _{\mathbb R^2} |r_\varepsilon (t)|^2 e^{-2 Y_\varepsilon } dx&\le C \Vert r_\varepsilon e^{- Y_\varepsilon } \Vert _{L^2}^2 \Big ( \Vert v_\varepsilon (t) \Vert _{L_{p \delta }^\infty }^p + \Vert w_\varepsilon (t) \Vert _{L_{p \delta }^\infty }^p \Big ) \Vert e^{- Y_\varepsilon } \Vert _{L_{- \delta }^\infty }^p \\&\le C(\omega ) \Vert r_\varepsilon e^{- Y_\varepsilon } \Vert _{L^2}^2 \Big ( \Vert v_\varepsilon (t) \Vert _{H^s_{\delta _1}}^p + \Vert w_\varepsilon (t) \Vert _{H^s_{\delta _1}}^p \Big ). \end{aligned}$$

By the Gronwall inequality, we obtain $r_\varepsilon (t) = 0$, so the uniqueness result follows. This implies that the whole sequence $\{v_{\varepsilon , n}\}_{n \in \mathbb N}$ converges to $v_\varepsilon$ in $\mathcal {C} ([0, T); H^s_{\delta _1})$.

### Proof of Theorem 1.2

The scheme of the proof is similar to the previous works on NLS equation with white noise potential.

We first note that by taking

$$A = Y_\varepsilon ,\quad V = \widetilde{\mathbf {:}\nabla Y^2_\varepsilon \mathbf {:}},$$

the conditions (4.3) and (4.4) hold almost surely uniformly in $\varepsilon \in (0, \frac{1}{2})$ by using the same reasoning as in the beginning of Sect. 7. In particular, we can use (5.4), (5.5), and (5.12) with *v* replaced by $v_\varepsilon$.

Fix $\delta _0 > 0$ and assume that $v_0 \in H^2_{\delta _0}$. Let $0< \varepsilon _2< \varepsilon _1 < \frac{1}{2}$ and define

$$\begin{aligned} r(t)=v_{\varepsilon _1}(t)-v_{\varepsilon _2}(t), \quad t\in [0,T]. \end{aligned}$$

Then, *r* satisfies the equation:

$$\begin{aligned} \imath \partial _t r&=\Delta r+r{\widetilde{\mathbf {:}\nabla Y^2_{\varepsilon _1}\mathbf {:}}}+v_{\varepsilon _2}({\widetilde{\mathbf {:}\nabla Y^2_{\varepsilon _1}\mathbf {:}}}-{\widetilde{\mathbf {:}\nabla Y^2_{\varepsilon _2}\mathbf {:}}}) - 2\nabla r \nabla Y_{\varepsilon _1} \\&\quad -2\nabla v_{\varepsilon _2} (\nabla Y_{\varepsilon _1}-\nabla Y_{\varepsilon _2}) -\lambda |v_{\varepsilon _1} e^{-Y_{\varepsilon _1}}|^{p} v_{\varepsilon _1}+\lambda |v_{\varepsilon _2} e^{-Y_{\varepsilon _2}}|^{p} v_{\varepsilon _2} ,\\ r(0)&=0. \end{aligned}$$

Using the equation for *r*, we can deduce that

8.4 $$\begin{aligned} \begin{aligned} \frac{1}{2} \frac{\textrm{d}}{\textrm{d}t}&\int _{\mathbb R^2} |r(t)|^2 e^{-2Y_{\varepsilon _1}} d x \\&= \text {Im}\int _{\mathbb R^2} \bar{r}(t) v_{\varepsilon _2}(t)({\widetilde{\mathbf {:}\nabla Y^2_{\varepsilon _1}\mathbf {:}}}-{\widetilde{\mathbf {:}\nabla Y^2_{\varepsilon _2}\mathbf {:}}}) e^{-2Y_{\varepsilon _1}} d x \\&\quad -2 \text {Im}\int _{\mathbb R^2} \bar{r}(t) \nabla v_{\varepsilon _2}(t)(\nabla Y_{\varepsilon _1}-\nabla Y_{\varepsilon _2})e^{-2Y_{\varepsilon _1}} d x \\&\quad -\lambda \text {Im}\int _{\mathbb R^2} \bar{r}(t) \left( |v_{\varepsilon _1}(t) e^{-Y_{\varepsilon _1}}|^{p } v_{\varepsilon _1}(t) - |v_{\varepsilon _2}(t) e^{-Y_{\varepsilon _2}}|^{p } v_{\varepsilon _2}(t)\right) e^{-2Y_{\varepsilon _1}} d x\\&=: I + II + III. \end{aligned}\qquad \end{aligned}$$

Using (2.46), (2.44), (3.16), (3.9), and interpolation with (5.4) and (5.5), we get that for $\alpha \in (0, \frac{1}{100})$, there exists $\delta > 0$ small enough and $\kappa > 0$ such that

8.5 $$\begin{aligned} \begin{aligned} |I|&\le C \Vert \bar{r}(t) v_{\varepsilon _2}(t) e^{-2Y_{\varepsilon _1}}\Vert _{\mathcal {B}^{\alpha }_{1,1, \delta }} \Vert {\widetilde{\mathbf {:}\nabla Y^2_{\varepsilon _1}\mathbf {:}}}-{\widetilde{\mathbf {:}\nabla Y^2_{\varepsilon _2}\mathbf {:}}}\Vert _{\mathcal {C}^{-\alpha }_{- \delta }}\\&\le C(\omega ) \varepsilon _1^\kappa \Vert r(t) \Vert _{H^{2\alpha }_{\delta }} \Vert v_{\varepsilon _2}(t)\Vert _{H^{3\alpha }_{\delta }} \Vert e^{-2Y_{\varepsilon _1}}\Vert _{\mathcal {C}^{3\alpha }_{- \delta }}\\&\le C(\omega )\varepsilon _1^\kappa \mathcal {P} \Big ( \Vert v_0\Vert _{H^1_{\delta _0}} \Big ). \end{aligned} \end{aligned}$$

Similarly, using (2.46), (2.44), (3.15), (3.9), (5.12), and (8.3), there exists $\delta > 0$ small enough and $\kappa > 0$ such that

8.6 $$\begin{aligned} \begin{aligned} |II|&\le C \Vert \bar{r}(t) \nabla v_{\varepsilon _2}(t) e^{-2Y_{\varepsilon _1}}\Vert _{\mathcal {B}^{\alpha }_{1,1, \delta }} \Vert \nabla Y_{\varepsilon _1}-\nabla Y_{\varepsilon _2} \Vert _{\mathcal {C}^{-\alpha }_{-\delta }}\\&\le C(\omega ) \varepsilon _1^\kappa \Vert r(t) \Vert _{H^{2\alpha }_\delta } \Vert \nabla v_{\varepsilon _2}(t) \Vert _{H^{3\alpha }_\delta } \Vert e^{-2Y_{\varepsilon _1}}\Vert _{\mathcal {C}^{3\alpha }_{-\delta }}\\&\le C(\omega ) \varepsilon _1^\kappa \mathcal {P} \Big ( \Vert v_0\Vert _{H^1_{\delta _0}} \Big ) \Vert v_{\varepsilon _2}(t) \Vert _{H^2_{-\delta }}^{C} \\&\le C(\omega ) \varepsilon _1^\kappa |\ln \varepsilon _2|^{C} \mathcal {P} \Big ( \Vert v_0\Vert _{H^2_{\delta _0}} \Big ). \end{aligned} \end{aligned}$$

Concerning *III*, using the embedding $H^{\frac{2}{2 - \eta }}_{\tilde{\delta }} \subset L^\infty _{\tilde{\delta }}$ ($\eta , \tilde{\delta }> 0$ small enough), (3.9), (5.4), (3.10), (5.12), and (8.3), there exists $\delta > 0$ small enough and $\kappa > 0$ such that

8.7 $$\begin{aligned} \begin{aligned} |III|&\le C \Vert r e^{-Y_{\varepsilon _1}} \Vert _{L^2}^2 \left( \Vert v_{\varepsilon _1}(t)\Vert _{L^\infty _{p \delta }}^{p}+\Vert v_{\varepsilon _2}(t)\Vert _{L^\infty _{p \delta }}^{p}\right) \Vert e^{-Y_{\varepsilon _1}}\Vert _{L^\infty _{-\delta }}^{p}\\&\quad + \Vert r\Vert _{L^2_\delta } \Vert v_{\varepsilon _2}(t)\Vert _{L^{2p + 2}_\delta }^{p+1} \Vert e^{-p Y_{\varepsilon _1}}- e^{-p Y_{\varepsilon _2}}\Vert _{L^\infty _{- p\delta }} \Vert e^{- Y_{\varepsilon _1}} \Vert _{L^\infty _{-\delta }} \\&\le C(\omega ) \Vert r e^{-Y_{\varepsilon _1}} \Vert _{L^2}^2 \Big ( \Vert v_{\varepsilon _1}(t)\Vert _{H^{\frac{2}{2 - \eta }}_{p \delta }}^{p}+\Vert v_{\varepsilon _2}(t)\Vert _{H^{\frac{2}{2 - \eta }}_{p \delta }}^{p} \Big ) \\&\quad + C(\omega ) \varepsilon _1^\kappa \Vert v_{\varepsilon _2}(t) \Vert _{H_\delta ^{\frac{2}{2 - \eta }}} \mathcal {P} \Big ( \Vert v_0\Vert _{H^1_{\delta _0}} \Big ) \\&\le C(\omega ) |\ln \varepsilon _2|^{\gamma } \mathcal {P} \Big ( \Vert v_0\Vert _{H^2_{\delta _0}} \Big ) \Vert r e^{-Y_{\varepsilon _1}} \Vert _{L^2}^2\\&\quad +C(\omega )\varepsilon _1^\kappa |\ln \varepsilon _2|^{C} \mathcal {P} \Big ( \Vert v_0\Vert _{H^2_{\delta _0}} \Big ), \end{aligned} \end{aligned}$$

for some $$\gamma \in (0, 1)$$.

Now, letting $\varepsilon _1 = 2^{-k}$ and $\varepsilon _2 = 2^{- (k+1)}$ for $k \in \mathbb N$, we combine (8.4), (8.5), (8.6), and (8.7) and apply the Gronwall inequality to obtain

$$\begin{aligned} \sup _{t \in [0, T)}&\int _{\mathbb R^2} |r (t)|^2 e^{- 2 Y_{2^{-k}}} dx \\&\le C(\omega ) 2^{-k \kappa } (k+1)^C \mathcal {P} \Big ( \Vert v_0\Vert _{H^2_{\delta _0}} \Big ) e^{ C(\omega ) T |\ln 2^{- (k + 1)}|^\gamma \mathcal {P} ( \Vert v_0\Vert _{H^2_{\delta _0}} )} \\&\le C(\omega ) 2^{-\frac{k \kappa }{2}} 2^{\tilde{\gamma } (k+1)C(\omega ) T \mathcal {P} ( \Vert v_0\Vert _{H^2_{\delta _0}} )} \mathcal {P} \Big ( \Vert v_0\Vert _{H^2_{\delta _0}} \Big ), \end{aligned}$$

where we used $e^{|\ln (1 + x)|^\gamma } \le C x^{\tilde{\gamma }}$ for $\gamma \in (0, 1)$, $\tilde{\gamma } > 0$ arbitrarily small, and $x > 0$ large. Thus, by (3.9), for any $\delta > 0$ we obtain

$$\begin{aligned} \Vert v_{2^{-k}} - v_{2^{- (k+1)}} \Vert _{\mathcal {C}([0, T); L^2_{- \delta })}^2 \le C(\omega ) 2^{-\frac{k \kappa }{4}}. \end{aligned}$$

Using interpolation along with the bounds (5.12) and (8.3), it is not hard to deduce that for any $s \in (1, 2)$, there exists $\delta _1 > 0$ such that $\{ v_{2^{-k}} \}_{k \in \mathbb N}$ is a Cauchy sequence in $\mathcal {C}([0, T); H^s_{\delta _1})$ and converges to some function $v \in \mathcal {C}([0, T); H^s_{\delta _1})$. By using similar steps as above, we can also deduce that

$$\begin{aligned} \sup _{\varepsilon \in (2^{-(k+1)}, 2^{-k}]} \Vert v_\varepsilon - v_{2^{-k}} \Vert _{\mathcal {C} ([0, T); H^s_{\delta _1})} \le C(\omega ) 2^{-k \tilde{\kappa }} \mathcal {P} \Big ( \Vert v_0\Vert _{H^2_{\delta _0}} \Big ), \end{aligned}$$

for some $\tilde{\kappa }> 0$, so that the whole sequence $\{ v_\varepsilon \}_{\varepsilon \in (0, \frac{1}{2})}$ converges to *v* in $\mathcal {C} ([0, T); H^s_{\delta _1})$ as $\varepsilon \rightarrow 0$. This finishes the convergence part of the theorem.

Lastly, we prove the uniqueness of the solution *v* in $\mathcal {C} ([0, T); H^s_{\delta _1})$ to the equation (1.7). Assume that *v* and *w* are two solutions to (1.7). Define

$$\begin{aligned} r' (t) = v (t) - w (t), \quad t \in [0,T). \end{aligned}$$

Then, $r'$ satisfies the equation:

$$\begin{aligned} \imath \partial _t r' = \Delta r' + r' \widetilde{\mathbf {:}\nabla Y^2\mathbf {:}} - 2 \nabla r' \nabla Y - \lambda e^{-p Y} (|v|^p v - |w|^p w), \quad r' (0) = 0. \end{aligned}$$

Using the equation for $r'$, we can deduce that

$$\begin{aligned} \frac{1}{2} \frac{\textrm{d}}{\textrm{d}t} \int _{\mathbb R^2} |r' (t)|^2 e^{-2 Y} dx = \lambda \text {Im}\int _{\mathbb R^2} \overline{r'} (t) \big ( |v (t)|^p v (t) - |w (t)|^p w (t) \big ) e^{-(p + 2) Y} dx. \end{aligned}$$

Thus, using the embedding $H^s_{\delta _1} \subset L_{\delta _1}^\infty$ and (3.11), there exists $\delta > 0$ small enough such that

$$\begin{aligned} \frac{1}{2} \frac{\textrm{d}}{\textrm{d}t} \int _{\mathbb R^2} |r' (t)|^2 e^{-2 Y} dx&\le C \Vert r' e^{- Y} \Vert _{L^2}^2 \Big ( \Vert v (t) \Vert _{L_{p \delta }^\infty }^p + \Vert w (t) \Vert _{L_{p \delta }^\infty }^p \Big ) \Vert e^{- Y} \Vert _{L_{- \delta }^\infty }^p \\&\le C(\omega ) \Vert r' e^{- Y} \Vert _{L^2}^2 \Big ( \Vert v (t) \Vert _{H^s_{\delta _1}}^p + \Vert w (t) \Vert _{H^s_{\delta _1}}^p \Big ). \end{aligned}$$

By the Gronwall inequality, we obtain $r' (t) = 0$, so the uniqueness result follows.

## Footnotes

1 Notice that the estimate (4.19) is very weak since the norm on the l.h.s is much weaker than the one on the r.h.s in both derivatives and weights. In fact, we have

$$\begin{aligned} 1 + \delta \ll \frac{\sqrt{\delta } (1 - \delta )}{\sqrt{2}} + 1 + \delta ^+ \quad \text {and} \quad \frac{\delta }{2} (1 - 3\delta ) \ll 4 \sqrt{\delta } \end{aligned}$$

for $\delta \ll 1$. Nevertheless, this weak estimate will be useful in the sequel (in particular it implies (4.24) below).
